# Author-paper affiliation network architecture influences the methodological quality of systematic reviews and meta-analyses of psoriasis

**DOI:** 10.1371/journal.pone.0175419

**Published:** 2017-04-12

**Authors:** Juan Luis Sanz-Cabanillas, Juan Ruano, Francisco Gomez-Garcia, Patricia Alcalde-Mellado, Jesus Gay-Mimbrera, Macarena Aguilar-Luque, Beatriz Maestre-Lopez, Marcelino Gonzalez-Padilla, Pedro J. Carmona-Fernandez, Antonio Velez Garcia-Nieto, Beatriz Isla-Tejera

**Affiliations:** 1 Department of Dermatology, Reina Sofia University Hospital, Cordoba, Spain; 2 Instituto Maimonides de Investigacion Biomedica de Cordoba (IMIBIC)/Reina Sofia University Hospital/University of Cordoba, Cordoba, Spain; 3 School of Medicine, University of Cordoba, Cordoba, Spain; 4 Department of Pharmacy, Reina Sofia University Hospital, Cordoba, Spain; Universidad Rey Juan Carlos, SPAIN

## Abstract

Moderate-to-severe psoriasis is associated with significant comorbidity, an impaired quality of life, and increased medical costs, including those associated with treatments. Systematic reviews (SRs) and meta-analyses (MAs) of randomized clinical trials are considered two of the best approaches to the summarization of high-quality evidence. However, methodological bias can reduce the validity of conclusions from these types of studies and subsequently impair the quality of decision making. As co-authorship is among the most well-documented forms of research collaboration, the present study aimed to explore whether authors’ collaboration methods might influence the methodological quality of SRs and MAs of psoriasis. Methodological quality was assessed by two raters who extracted information from full articles. After calculating total and per-item Assessment of Multiple Systematic Reviews (AMSTAR) scores, reviews were classified as low (0-4), medium (5-8), or high (9-11) quality. Article metadata and journal-related bibliometric indices were also obtained. A total of 741 authors from 520 different institutions and 32 countries published 220 reviews that were classified as high (17.2%), moderate (55%), or low (27.7%) methodological quality. The high methodological quality subnetwork was larger but had a lower connection density than the low and moderate methodological quality subnetworks; specifically, the former contained relatively fewer nodes (authors and reviews), reviews by authors, and collaborators per author. Furthermore, the high methodological quality subnetwork was highly compartmentalized, with several modules representing few poorly interconnected communities. In conclusion, structural differences in author-paper affiliation network may influence the methodological quality of SRs and MAs on psoriasis. As the author-paper affiliation network structure affects study quality in this research field, authors who maintain an appropriate balance between scientific quality and productivity are more likely to develop higher quality reviews.

## Introduction

Psoriasis is a chronic immune-mediated inflammatory skin disease that affects 2-3% of the population [1]. Moderate-to-severe forms of this disease are associated with significant comorbidity, impaired quality of life, and high direct and indirect costs [2]. More than 30,000 studies (original, reviews, and letters) on this topic have been published in MEDLINE prior 2016 [3]. Over time, the processes of scientific review and collaboration have evolved. Systematic reviews (SRs) and meta-analysis (MAs), which enable the quantitative synthesis of SRs, have become the standard approaches to the summarization and synthesis of primary medical research in response to a clearly formulated research question [4]. However, methodological flaws can reduce the validity of conclusions drawn from these studies, and thus undermining the quality of decision making.

In recent decades, co-authorship has become an objective of scientific collaboration [5, 6]. The number of papers published during the last 35 years has kept pace with the number of scientists working in each field, as demonstrated by several studies that have observed progressive increases in local and international collaborations between authors and institutions [7]. This growth is considered a sign of evolution and improvement among forms of scientific investigation [3].

Recently, Gonzalez-Alcaide *et* *al*. characterized the structures of co-authorship networks in the field of psoriasis research through topologic analyses, community discovery, and studies of evolution during the last decades [3]. However, the authors did not explore whether these network structures increased the quality of scientific evidence. Although collaborations between several authors appear to facilitate productivity, co-authorship does not ensure scientific quality [5]. When papers receive the same weight in bibliometric indices, regardless of authorship status (i.e., single-authored, first-authored, or co-authored with hundreds of others), investigators might less rigorously select increasing numbers of co-authors [8], a practice that may even result in beneficial reciprocal co-authorship [9]. However, unscrupulous teams that extensively practice mutual co-authorship will increase their competitiveness against scientists with more rigorous authorship standards [10].

In short, the increasing number of authors over time does not merely reflect an increase in the required work per paper. Accordingly, the potential effects of social relationships on the success (bibliometric indices) and quality (methodological appropriateness) of scientific articles must be addressed. In this study, we therefore used the AMSTAR tool to determine whether differences in the methodological quality of SRs and MAs exist with regard to the author-paper affiliation network in the field of psoriasis research.

## Materials and methods

### Study types and inclusion criteria

We included SRs or MAs that applied systematic methods to identify, select, and analyze clinical trials (CTs) or observational studies of skin psoriasis published in scientific journals. Historical articles, abstracts of congresses, case reports, surveys, narrative reviews, narrative reports (i.e. reports focused on an understanding of a concept), clinical practice guidelines, consensus documents, MAs conducted without a systematic literature search, literature reviews, integrative reviews, and SRs that did not meet any of the AMSTAR criteria were excluded from our analysis. There were no limitations on the year of publication, language, or study population.

### Search methods

We established and published an a priori protocol in the PROSPERO International Prospective Register of Systematic Reviews (CRD42016041611). A search of SR and MA protocol registries, including PROSPERO and the Cochrane Database of Systematic Reviews, revealed that no similar studies were in progress as of May 2016. SRs and MAs published up to July 4, 2016 were identified in MEDLINE, EMBASE, and the Cochrane Database through a comprehensive systematic Boolean search using MSeH terms (‘psoriasis’/exp or psoriasis) and (‘meta analysis’ or ‘systematic review’). We identified additional eligible studies by searching the reference lists of the included SRs, MAs, and health technology assessment (HTA) reports. We contacted study authors when necessary to identify further information that we may have missed.

### Methods for identification and selection

A few authors independently performed all tasks related to study filtering and selection (FG-G, MG-P, PJG-F, and BI-T) and data extraction (FG-G, JG-M, MG-P, MA-L, PJG-F, and BI-T). Screening was performed in two stages. In the first stage, abstracts downloaded from literature searches were screened, and any study that clearly failed to meet the eligibility criteria was rejected. In the second stage, full papers were retrieved for the remaining candidate study and reviewed to identify all SRs and MAs that met the eligibility criteria. In doubtful or controversial cases, all discrepancies identified during the first stage and throughout the review were resolved via discussion; for select cases, this process involved a different investigator (JR).

### Assessment of methodological quality

Data were analyzed from August 30 to September 15, 2016. A 10-study pilot evaluation was performed prior to evaluation of the selected articles to standardize usage and eliminate inconsistencies. Two investigators (JLS-C, MA-L) independently assessed the methodological quality of each SR using the AMSTAR tool data abstraction forms and 11 AMSTAR criteria, and quality assessment discrepancies were discussed with a third author (JR) until an agreement was reached. Although we did not use the AMSTAR score as an inclusion criterion, we identified and discussed differences in quality between reviews and used the review quality assessment to interpret the results of reviews synthesized in this overview. The 11 AMSTAR criteria were rated as “yes” (criteria were met), “no” (criteria were not met), “cannot answer” (unclear information), or “not applicable” (criteria could not be evaluated because of the design of background studies in the reviews) (Table D in S1 File). For all items except item 4, ratings of “yes” received scores of 1, whereas “no”, “cannot answer” and “not applicable” received scores of 0. For item 4, a rating of “no” (i.e., the review did not exclude unpublished or grey literature) was considered adequate. The highest possible AMSTAR score was 11, and scores were used to classify review quality as follows: 0–4 = low quality, 5–8 = moderate quality, and 9–11 = high quality. Total AMSTAR scores were summarized descriptively as medians and interquartile ranges or as percentages of achievement per item.

### Data extraction

We independently obtained metadata from every article, author, and journal related to studies that fulfilled the inclusion criteria (title, authors’ names, institutions, and countries from InCites™ Journal Citation Reports^®^, Thomson Reuters) and for every author (H-indexes, number of publications, total citations, and numbers of collaborators for studies from Scopus^®^, Elsevier). We additionally standardized discrepancies in the signatures of single authors with same name; these were mainly found when one or more first or last names were included, the first name was spelled out or abbreviated, or typographical errors were present.

### Network analysis

We investigated the architecture of the constructed author-paper affiliation network using the list of included SRs and MAs as seminal nodes. The final network formed a bipartite graph with two node types representing authors and reviews. An affiliation network is the most complete representation of a collaboration study; in addition to such bipartite representations, one can particularly investigate both author-oriented and paper-oriented properties. We analyzed differences in whole network statistics, centrality, connected components, and community structures among three subnetworks of the entire graph: the low, the moderate, and high methodological quality subnetworks.

#### Whole network statistics

Network and node descriptives were analyzed using the R-language iGraph software package (http://igraph.org/r/). *Nodes* represented authors or reviews, and *edges* connected authors with the articles to which they contributed. *Papers* *by* *author* indicated the productivity of each author, while *authors* *per* *paper* accounted for the level of co-authorship around single papers. *Edge* *density* was used as a measure of network effectiveness and defined as the ratio of the number of edges to the number of possible edges. The *average* *distance*, or average shortest path of a graph, corresponded to the summa of all shortest paths between node couples divided by the total number of node couples; this latter parameter reflects graph “compactness”, or the overall tendency of nodes to stay in proximity. *Network* *diameter*, the length of the shortest path between two nodes in the network, was calculated by summing the shortest paths between all pairs of nodes and dividing this value by the total number of pairs. This latter parameter indicates the average number of steps required to move between two members of a network.

#### Centrality

Indicators of centrality identify the most important nodes within a graph. We analyzed node centrality by calculating *node* *degree*, *closeness* (i.e., centrality based on distance to others in the graph), *betweenness* (i.e., centrality based on a broker position that connected others), and *eigenvector* (i.e., centrality proportional to the sum of connection centralities). Kleinberg’s hub and authority scores were also calculated.

#### Connected components

Not every network is completely connected. Some may contain isolates, or groups of nodes connected to each other but not to the rest of the network. Those parts of the network are called components, and the largest component is the *giant* *component*.

#### Community detection

In studies of complex networks, a network is said to have community structure if the nodes of the network can be easily grouped into (potentially overlapping) sets of nodes such that each set features dense internal connections. We explored community detection in our network using the edge betweenness (Newman-Girvan) and propagating label methods. *Connectance*, an extremely intuitive network property, was used to express the extent to which the potential connections between nodes are realized. A graph is highly connected if for every pair of nodes, there is a path between them. Transitivity or clustering coefficient measure the probability that the adjacent vertices of a vertex are connected. The *clustering* *coefficient* of an undirected graph is simply the ratio the number of triangles in the entire network and dividing it by the number of possible triangles in the graph. *Compartmentalization* is the division of a network into relatively independent sub-networks. *Assortativity* is a measure of the likehood for nodes to connect to other nodes with similar degrees. The assortativity coefficient *r* ranges between -1 and 1. Positive assortativity coefficient means than nodes tend to connect to other nodes with similar degree, while negative assortativity coefficient means that nodes tend to connect to other nodes with different degrees. Networks could be divided into dense subnetworks (modules or communities) linked by only sparse connections. Here, *modularity* was used to measure the strength of division of a network into subnetworks; highly modular networks feature dense connections between nodes within modules but sparse connections between nodes in different modules.

### Differences between protocol and overview

Our planned search strategy, recorded in PROSPERO, was compared with the final reported review methods. We did not add, omit, or change the outcomes after our protocol was published. We note that we only restricted our retrieval to English-language reviews because of time limitations placed on project completion.

### Data analysis

We used a range of approaches to present the results of included reviews. We captured article and journal metadata using standardized data extraction templates implemented in *AppSheet*, a custom mobile app based on Google forms(https://www.appsheet.com/). Inter-rater agreement was tested using Cohens’s *kappa* (for squared) using the R-language *irr* package (R Project for Statistical Computing, Vienna, Austria). Kappa values can range from -1.0 to 1.0, with -1.0 indicating perfect disagreement below chance, 0.0 indicating agreement equal to chance, and 1.0 indicating perfect agreement above chance. Generally, a kappa value of ≥0.70 indicates adequate inter-rater agreement; here, a value of ≥0.65 was chosen to indicate sufficient agreement.

### Reproducibility of results

Several R language packages were used to produce graphs and perform statistical analyses. Our analysis can be fully reproduced using several raw data source files and R scripts stored at our github hosting repository (https://github.com/info4cure/coAuthorshipNetworkArchitecturePaper).

### Ethics

No ethical approval was required.

## Results

### Search results

Our database search yielded 1195 potentially relevant titles (699 EMBASE & 160 MEDLINE, 474 EMBASE, 22 MEDLINE, and 4 Cochrane Database). After excluding duplicates and screening abstracts, 304 studies remained eligible for full-text review. Finally, 220 reviews from 92 peer-reviewed journals were subjected to quality assessment (see Figs A and B in S1 File for search strategy and the PRISMA flowchart, and Tables B and C in S1 File for included and excluded studies, respectively).

### General characteristics of reviews

The published reviews had a total of 741 authors from 520 different institutions and 32 countries, with medians of 5 (2–20) and 3 (1–16) participating authors and institutions per review, respectively. The authors’ H-indexes varied widely among studies, with a median of 14 (1–108). Overall, roughly half of the assessed studies were published in dermatology journals (54.4%, 120/220); however, that rate increased to 83.6% (184/220) during the last 5 years.

### Assessment of methodology quality

The AMSTAR statement was used to assess methodology quality after substantial inter-rater agreement was achieved [*kappa* = 0.75, 95% confidence interval (CI), 0.69-0.82]. The median AMSTAR score of all 220 included SRs and MAs was 6 (4–8), and 17.2%, 55%, and 27.8% of reviews were classified as high, moderate, or low methodological quality, respectively. The AMSTAR items with the lowest compliance rates were Q5 (“list of studies provided”, 11.3%), Q10 (“publication bias assessed”, 27.8%), Q4 (“status 186 of publication included”, 39.5%), and Q1 (“a priori design provided”, 41%). The best compliance rate was observed for item Q6 (“characteristics of the included studies were provided”, 89.5%).

### Statistical properties of author-paper affiliation subnetworks

**Fig 1:**
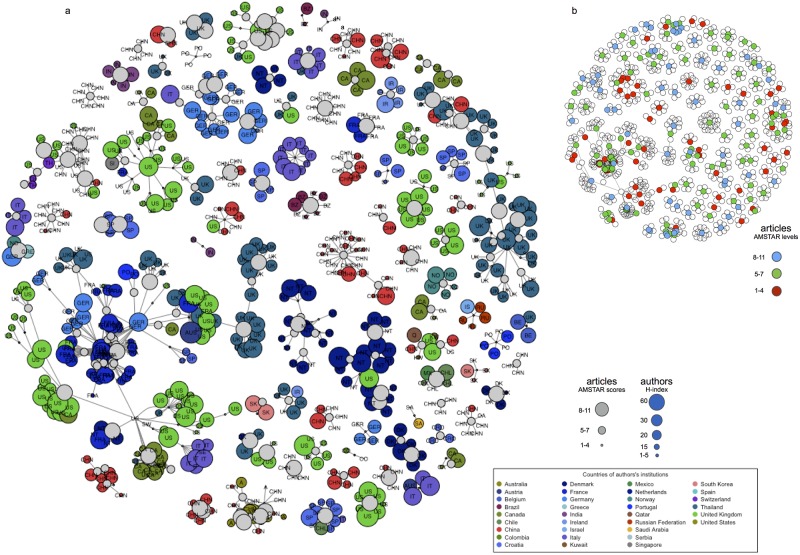
Whole author-paper affiliation network. (a). Nodes that represent authors are colored and labeled based on their institution’s country. Grey nodes represent the reviews on psoriasis which were finally included. Node size is proportional to the author’s H-index or AMSTAR score respectively. Edges connect both types of nodes, thus every author and their collaborators are linked to the shared publication. (b). Same network, although only nodes representing articles are colored based on AMSTAR levels.

Fig 1a depicts the entire co-authorship network, with nodes representing reviews (grey) or authors (colors). Authors’ names have been replaced by the corresponding institutions’ countries to simplify the analysis, and node sizes are proportional to the corresponding review’s AMSTAR score or author’s H-index. The entire network comprised 735 authors and 220 papers linked by 1,349 unique undirected edges (Table 1). An average of 1.8 (1–26) papers per author corresponded to publishing productivity regarding SRs about psoriasis, and each author had an average of 8.9 (2–14) collaborators in his or her network. Fig 1b depicts the same network, although only nodes representing articles are colored based on AMSTAR scores.

**Table 1:** Summary statistics for the co-authorship networks based on methodology quality.

			All	Low quality	Moderate quality	High quality
1. Whole network statistics	Nodes	*Number* *of* *nodes*	955	288	576	216
		*Number* *of* *authors*	735	228	455	181
		*Number* *of* *papers*	220	61	121	35
	Edges	*Number* *of* *edges* (*unique*)	1,349	371	763	215
		*Papers* *by* *author*	1.8 (1-26)	1.6 (1-10)	1.7 (1-16)	1.2 (1-3)
		*Author* *per* *paper*	6.3 (2-20)	6.2 (2-15)	6.3 (2-20)	6.1 (3-16)
		*Average* *collaborators*	8.9 (2-14)	9.3 (2-15)	8.9 (2-20)	7.9 (3-16)
		*Edge* *density*	2.8	2.5	2.6	1.9
		*Average* *distance*	6.3	3.2	2.8	2.7
		*Network* *diameter*	14	8	6	10
2. Centrality		*Activity*: *Degree*	0.024	0.043	0.03	0.065
		*Efficiency*: *Closeness*	0.0004	0.001	0.0003	0.0008
		*Control*: *Betweenness*	0.034	0.020	0.005	0.013
		*Overall*: *Eigenvector*	0.96	0.92	0.95	0.97
3. Community detection		*Connectance*	0.003	0.009	0.004	0.009
		*Clustering* *coefficient*	0	0	0	0
		*Compartmentalization*	0.004	0.015	0.007	0.026
		*Assortativity*	0.44	0.12	0.28	-0.61
		*Number* *of* *communities*	90	37	65	25
		*Modularity*	0.90	0.76	0.89	0.93
		*Giant* *component* *size* (%)	225	60 (20.8%)	69 (12%)	31 (14.3%)

Results of main statistics of whole author-paper affiliation network and included subnetworks corresponding to SRs and MAs on psoriasis of low, moderate, and high methodological quality.

**Fig 2:**
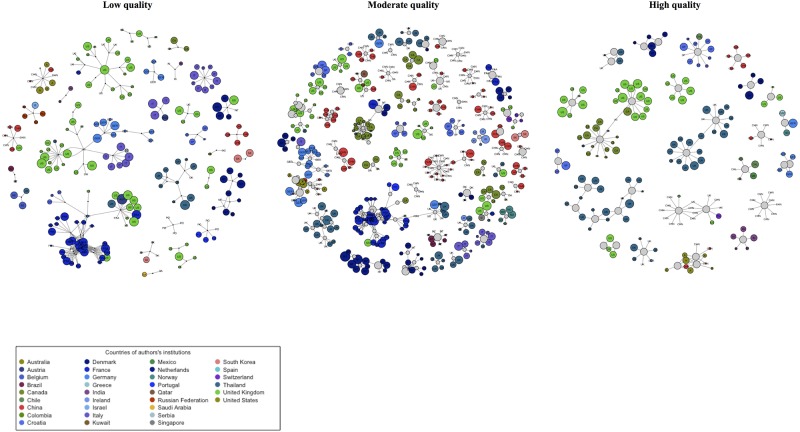
Author-paper affiliation subnetworks based on methodological quality of the reviews. Nodes that represent authors are colored and labeled based on their institution’s country. Grey nodes represent the SRs and MAs on psoriasis which were finally included. Node size is proportional to the author’s H-index or AMSTAR score respectively. Edges connect both types of nodes, thus every author and their collaborators are connected to the shared publication.

**Fig 3:**
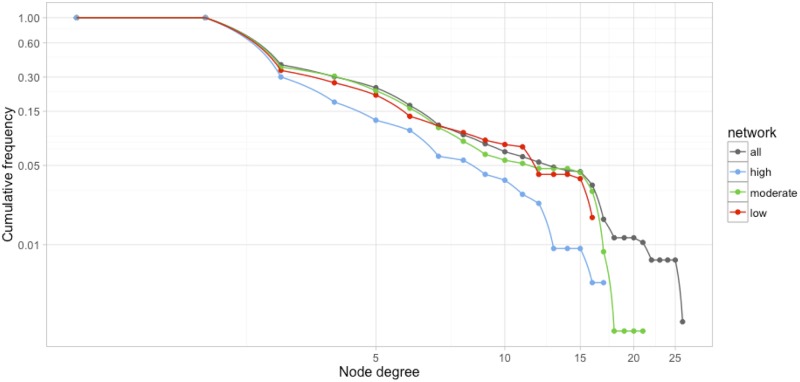
Cumulative frequency of node degree distributions. The cumulative node degree distribution shows the cumulative number of nodes of each degree class. The distribution of degree values over all nodes characterizes the network as a whole.

Fig 2a–2c depicts three subnetworks of SRs and MAs according to methodological quality. When compared with the entire network, the cumulative node degree frequencies of these three subnetworks followed a power-law distribution truncated by exponential distribution (Fig 3). Moderate- and low-quality subnetworks followed similar patterns of node-degree distribution, whereas differences of the high-quality subnetwork were attributed to a node-degree distribution that ran parallel to but below the moderate and low subnetworks and had a more rapid rate of decay. Compared with the other subnetworks, the high-quality subnetwork was larger but had a lower connection density, suggesting a higher network diameter. The high-quality subnetwork also contained relatively fewer nodes (authors and reviews), reviews by authors, and collaborators (Table 1). However, the subnetworks featured similar average numbers of authors per paper. Furthermore, the high-quality subnetwork was highly compartmentalized, with several modules representing a small number of poorly interconnected communities, and negative assortativity. The clustering coefficients of the three subnetworks are zero; that means that the neighbors of any node (papers or authors) are not likely to be connected with each other, which is an expected result based on the definition of bipartite networks where the smallest possible cycle is a 4-cycle, not a triangle. High quality SRs subnetwork showed the lowest number of communities with the highest modularity. This means that nodes are poorly connected, thus authors linked to papers constitute isolates modules or communities.

The analysis of hubs and authorities, k-core decomposition, and network components identified the giant component of every subnetwork (Fig C in S1 File). Giant components are quite small as compared with other kind of networks. In our case, nodes of giant component of small and moderate quality subnetworks represent <20% of the total number of nodes. Even at this reduce scale, the proportion of nodes in the giant component decreases with assortativity. Intuitively, if the giant component is relatively small after all nodes have been included, many isolated components result, and the number of communities and modularity increase. The giant components of the low and the moderate methodological quality subnetworks overlaped 60-70% of authors, who were mainly from French institutions (Table D in S1 File). In contrast, the giant component of the high methodological quality subnetwork comprised a unique group of authors who were affiliated with institutions in the USA, UK, and Canada.

### Differences in author’s productivity vs. scientific quality

**Fig 4:**
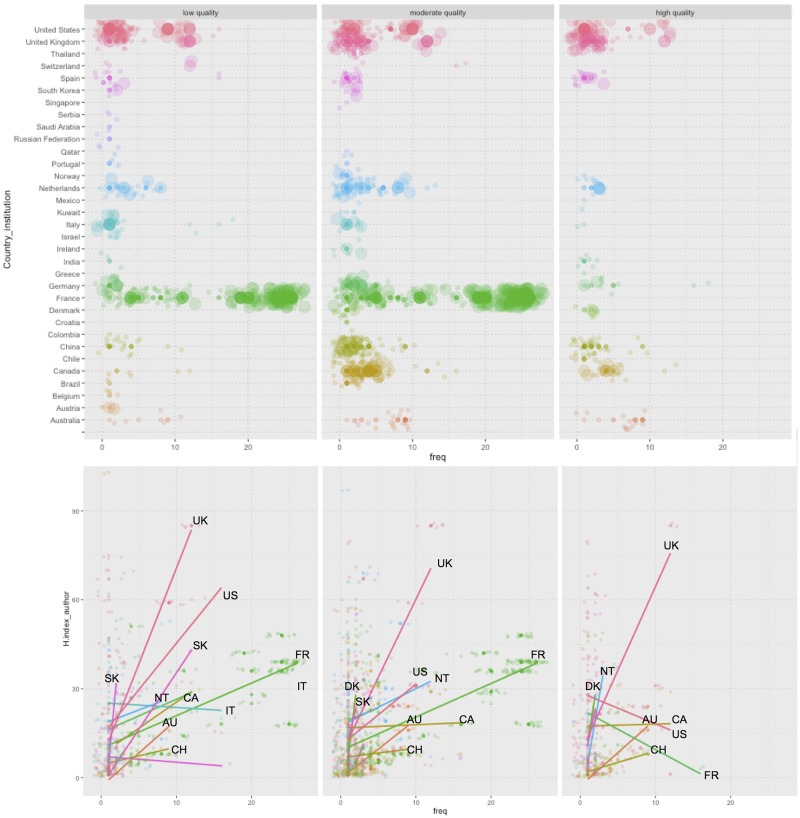
Influence of authors’ scientific quality and productivity on methodological quality of SRs and MAs about psoriasis. Panel (a-c): Bubble plot that represents the number of publications by author. Bubble size is proportional to the author’s H-index. Authors are sorted by their institution’s country. Panel (d-f) represents a scatter plot of author’s H-index vs. number of authored publications. Smoothed fitted lines represent predictions using linear regression for every country. Points and lines are colored based on author institution country.

Fig 4a–4c shows the relationships of SR number and other factors by author while accounting for each author’s H-index and the country of each institution. The largest number of authors with the highest number of SRs was observed in France, which had a particularly high frequency of authors, publications and collaborators for reviews of low and moderate methodological quality. However, this pattern was only observed among reviews of low and moderate methodological quality, and not among high-quality reviews. For the latter, the representation of French authors was lower than that of authors from other countries such as the USA or the UK.

Fig 4d–4f presents three scatter plots of H-index values vs. the number of publications per author. The colors of points and lines correspond to the countries of the various institutions, as shown in the top panels. Smoothed lines were fitted using a linear regression model by country. In panels d-f, the slope of each line represents the relationship between scientific quality and author productivity. As the h-index reflects both the number of publications and the number of citations per publication, these parameters should remain balanced as different authors are considered; accordingly, a balanced relationship between productivity and scientific quality would be expected during an author’s lifetime. Authors with high levels of productivity produced reviews of moderate or low methodological quality, and conversely, authors with a productivity bellow the median published reviews of moderate or high methodological quality (Fig D in S1 File). The number of publications (i.e., SRs and other studies of psoriasis) should increase as the H-index increases. A deviation in this balance would indicate a high degree of specialization in developing systematic reviews, although this would not only hold true for psoriasis. This phenomenon was observed for some authors from the UK, USA, and Canada; in other words, those with high h-index values had low numbers of published high methodological quality reviews on psoriasis. In contrast, although authors with similar H-indexes who were affiliated with French or Chinese institutions had much higher productivity, their reviews were mostly of low or moderate methodological quality.

## Discussion

### Synthesis of the findings

Ours is the first study to compare the effects of different models of collaboration between researchers and the associated quality of their co-authored SRs of psoriasis. Our first conclusion is that SRs with the highest levels of methodological quality were produced by collaborative networks wherein authors with medium-high levels of scientific expertise but little productivity in this area belonged to very modular collaborative environments; furthermore, these environments were weakly connected and dominated by institutions from the UK, USA, or Canada. By contrast, SRs with the lowest levels of methodological quality were produced by more cohesive networks of collaborators that were better connected, appeared within well-defined communities, more frequently published reviews of psoriasis, and included greater numbers of collaborators. The authors in the latter subnetworks tended to belong to several institutions in countries such as France or China. As mentioned earlier, Gonzalez-Alcaide *et* *al*. recently analyzed the evolution of scientific collaboration on psoriasis research and identified active multicenter groups as a key element that ensures the progress of knowledge in this field [3]. These authors explored collaborative patterns among authors and found that topology might influence the psoriasis literature productivity, but did not assess the quality of those products. Montjoye *et* *al*. stated that repeated co-authorship was associated with better scientific performance [11] and argued that weak ties between scientists were unlikely to enhance access to information or to improve performance. Sahu *et* *al*. between authorship and article quality in the field of oceanography [12] and found that multi-authored manuscripts were more likely to be published in high impact factor journals. However, these authors analyzed study quality using journal impact factors, rather than the scientific quality of the articles. By contrast, we compared the quality levels of included studies and searched for differences in the structures of co-authorship subnetworks. Notably, we observed that during the last 15 years, SRs and MAs of psoriasis with the best methodological quality were conducted by small, modular, and unconnected groups of authors with high levels of scientific expertise but low productivity regarding this topic. To measure the effects of research, citation-based measures such as the h-index have been proposed as quantitative and objective proxies of the quality of research, scientists, and institutions [13]. In our study, groups of co-authors with medium or high h-indexes were identified in both low and the high methodological quality subnetworks. Quality was not predominantly affected by bibliometric indices, but rather by the degree of adherence to well-established systematic methodologies. Indeed, possession of the technical skills needed to perform such reviews according to existing guidelines and statements is likely more important than possession of the broader knowledge and experience to conduct clinical or experimental research on psoriasis. In other words, a collaborative environment does not always ensure that the final product will be of high scientific quality. In the specific case of SRs and MAs, it appears that it would be better to collaborate with groups that have more experience in relevant methodology, even if they lack cohesion and high degree of connectivity and do not comprise multiple authors. Human social networks (e.g., a coauthorship network) tend to have positive assortativity, as it was observed in the case of low and moderate quality subnetworks. However, our high quality subnetwork seems to have a profile more similar to the technological networks which tend to have negative assortativity [14]. Gómez-García *et* *al*. have recently observed that most of the SRs and MAs published about psoriasis by The Cochrane Collaboration achieved the highest AMSTAR scores [15]. The Cochrane Collaboration is a global independent network of researchers involved in developing SRs and MAs [16]. Probably, other factors, such as the number of authors with conflict of interest or the source of funding, may influence the differences in assortativity properties that we found between the low/medium and the high quality subnetworks [15]. Long *et* *al*. analyzed studied the roles and associated activities of key players within the research network structure of a translational cancer research network [17] and observed that geographic proximity and previous working relationships both had significant effects on the choice of current collaboration partners [18]. Geographic proximity remains a significant influence on the choice of partners, perhaps indicating a preference for local collaboration. The giant components of our low and moderate quality subnetworks indicated an enrichment of closely linked institutions from France. One potential explanation is that geographic proximity may reflect the physical degree of closeness, similarities in training, and educational competencies or experiences of these co-authors [19]. However, Mayrose *et* *al*. found that although collaborations between remote researchers are relatively rare, the quality of work produced by such associations is significantly higher than that produced by closely linked scientists [20]. This finding supports our observation of a smaller giant component in our high-quality subnetwork of co-authors from the USA, UK, and Canada.

### Strengths and limitations

One strength of our work was the use of a large sample size of publications following an *a* *priori* protocol involving a systematic search, filtering, data extraction, and analysis. The present study, however, also had several limitations that place constraints on its generalizability. First, our study focused on psoriasis, and specifically on SRs and MAs of this topic. We do not know whether our results would be widely applicable to other classes of study or topics of research. Therefore, the next step would involve the generation of stronger overall evidence to support our findings regarding the influence of the author-paper affiliation network structure on variations in scientific productivity and quality in other areas of research. Second, although this analysis was conducted at an individual author level, we simplified our procedure by establishing the country of the institution to which each author belonged as the unit of reference. However, this required an assumption that authors did not move to other institutions or countries of residence during the study period. Third, we did not consider the dynamic effects of other indices such as the H-index or number of publications, as our networks were constructed to account for articles published in different years; accordingly, the values of these parameters were determined using the date of metadata extraction as the reference. However, we believe that all authors underwent similar personal H-index evolution over time; in addition, we compared the ratio of the H-index to the number of publications for normalization, thus allowing an easier comparison. We also must consider that a large discrepancy between a high H-index and a low number of SRs on psoriasis is a very likely scenario among researchers who specialize in this type of review; however, these researchers are also likely to have published other reviews, particularly SRs of other diseases. In fact, we observed this scenario in our high-quality subnetwork. However, it is difficult to establish a single explanation that would encompass other cases, such as authors with low H-indexes and few publications or low H-indexes and many publications. An explanation of such cases would require additional information about the authors to establish partnerships, or variables such as the number of conflicts of interests and sources of funding. Although this type of analysis is more complex, it would be interesting to determine the influence of financial factors on the productivity and quality of these collaborative networks.

### Conclusions and future research

In summary, the methodological quality levels of SRs and MAs of psoriasis appear to be suboptimal. In this field of research, patterns of collaborations between authors and institutions were found to affect quality, and the maintenance of an appropriate balance between the scientific trajectory and productivity is more likely to lead to higher quality reviews. By contrast, environments in which authors have high levels of productivity and a greater degree of collaboration but less experience in conducting SRs and MAs will produce reviews of moderate or low methodological quality.

## Supporting information

S1 File Search strategy for identifying systematic reviews and meta-analyses in EMBASE (Table A), list of included studies (Table B), list of non included studies (Table C), AMSTAR checklist (Table D), PROSPERO register file (Table E), giant components in co-authorship subnetworks (Table F), PRISMA Flowchart for Overview of Systematic Reviews and Meta-analysis on psoriasis included and excluded in the study (Fig A), co-authorship networks modularity (Fig B), community analysis of co-authorship of low, moderate, and high methodological quality reviews derived subnetworks (Fig C), visualization of unique and intersection analysis of authors who have published systematic reviews classified by methodological quality and author productivity (Fig D).
(PDF)
