# Supplementary material for: Author-paper affiliation network architecture influences the methodological quality of systematic reviews and meta-analyses of psoriasis
Source: PLoS One. 2017 Apr 12;12(4):e0175419. doi: 10.1371/journal.pone.0175419 (PMC5389828; doi:10.1371/journal.pone.0175419)

SUPPLEMENTARY INFORMATION

Title: Architecture of author-paper affiliation network influences methodological quality of systematic reviews and meta-analyses on psoriasis

Authors: Juan Luis Sanz-Cabanillas, Juan Ruano, Francisco Gomez-Garcia, Patricia Alcalde Mellado, Jesus Gay-Mimbrera, Macarena Aguilar-Luque, Beatriz Maestre-Lopez, Marcelino Gonzalez-Padilla, Pedro J. Carmona-Fernandez, Antonio Velez Garcia-Nieto, Beatriz Isla-Tejera

Content:

1. Supplementary Tables.

Table A. Search strategy for identifying systematic reviews and meta-analyses in EMBASE.

Table B. List of included studies.

Table C. List of non included studies.

Table D. AMSTAR checklist.

Table E. PROSPERO register file.

Table F. Giant components in co-authorship subnetworks.

2. Supplementary Figures.

Figure A. PRISMA Flowchart for Overview of Systematic Reviews and Meta-analysis on psoriasis included and excluded in the study.

Figure B. Co-authorship networks modularity.

Figure C. Community analysis of co-authorship of low, moderate, and high methodological quality reviews derived subnetworks.

Figure D. Visualization of unique and intersection analysis of authors who have published systematic reviews classified by methodological quality and author productivity.

1. Supplementary Tables.

Table A. Search strategy for identifying systematic reviews and meta-analyses in EMBASE.

# Query	Search query	Number of records retrieved
#1:	'psoriasis'/exp OR 'psoriasis'	64,000
#2:	'systematic review'	156,373
#3:	'meta analysis'	169,723
#4:	#2 OR #3	255,843
#5:	#1 AND #4	1,254
#6:	#5 AND '[1-1-1945]/sd' NOT '[4-7-2016]/sd'	1,195

Table B. List of included studies.

High quality

A.R. M, J. M, M. C, G. D. Topical treatments for chronic plaque psoriasis. Cochrane Database Syst. Rev.2009;CD005028.

Armstrong AW, Harskamp CT, Armstrong EJ. The association between psoriasis and obesity: a systematic review and meta-analysis of observational studies. Nutr. Diabetes 2012;2:e54.

Armstrong AW, Harskamp CT, Armstrong EJ. Psoriasis and metabolic syndrome: A systematic review and meta-analysis of observational studies. J. Am. Acad. Dermatol. 2013. p. 654–62. 4.

Atwan A, Ingram JR, Abbott R, Kelson MJ, Pickles T, Bauer A, et al. Oral fumaric acid esters for psoriasis: Abridged Cochrane systematic review including GRADE assessments. Br. J. Dermatol. 2016; 5.

Candia R, Ruiz A, Torres-Robles R, Chávez-Tapia N, Méndez-Sánchez N, Arrese M. Risk of non-alcoholic fatty liver disease in patients with psoriasis: A systematic review and meta-analysis. J. Eur. Acad. Dermatology Venereol. 2015;29:656–62. 6.

Chen X, Yang M, Cheng Y, Liu GJ, Zhang M. Narrow-band ultraviolet B phototherapy versus broad-band ultraviolet B or psoralen-ultraviolet A photochemotherapy for psoriasis. Cochrane database Syst. Rev.2013;10:CD009481.

Coto-Segura P, Eiris-Salvado N, González-Lara L, Queiro-Silva R, Martinez-Camblor P, Maldonado-Seral C, et al. Psoriasis, psoriatic arthritis and type 2 diabetes mellitus: A systematic review and meta-analysis. Br. J. Dermatol. 2013;169:783–93. 8.

Deng S, May BH, Zhang AL, Lu C, Xue CCL. Plant extracts for the topical management of psoriasis: A systematic review and meta-analysis. Br. J. Dermatol. 2013;169:769–82. 9.

Dommasch E, Abuabara K, Shin D, Nguyen J, Troxel A, Gelfand J. The Risk of Infection and Malignancy with Tumor Necrosis Factor Antagonists in Adult Patients with Psoriatic Disease: A Systematic Review and Meta-analysis of Randomized Controlled Trials. J Am Acad Dermatol. 2011;64:1035–50.

Dowlatshahi EA, Van Der Voort EAM, Arends LR, Nijsten T. Markers of systemic inflammation in psoriasis: A systematic review and meta-analysis. Br. J. Dermatol. 2013;169:266–82.

Dowlatshahi EA, Wakkee M, Arends LR, Nijsten T. The prevalence and odds of depressive symptoms and clinical depression in psoriasis patients: a systematic review and meta-analysis. J. Invest. Dermatol; 2014;134:1542–51.

Jacobs A, Rosumeck S, Nast A. Systematic review on the maintenance of response during systemic antipsoriatic therapy. Br. J. Dermatol. 2015;173:910–21.

Li X, Kong L, Li F, Chen C, Xu R, Wang H, et al. Association between psoriasis and chronic obstructive pulmonary disease: A systematic review and meta-analysis. PLoS One. 2015;10.

Li X, Xiao Q, Li F, Xu R, Fan B, Wu M, et al. Immune Signatures in Patients with Psoriasis Vulgaris of Blood-Heat Syndrome: A Systematic Review and Meta-Analysis. Evidence-based Complement. Altern. Med. [Internet]. 2016;2016:1–11.

Lin VW, Ringold S, Devine EB. Comparison of Ustekinumab With Other Biological Agents for the Treatment of Moderate to Severe Plaque Psoriasis: A Bayesian Network Meta-analysis. Arch. Dermatol. [Internet]. 2012;148:1403–10.

Malhotra A, Shafiq N, Rajagopalan S, Dogra S, Malhotra S. Thiazolidinediones for plaque psoriasis: a systematic review and meta-analysis. Evid. Based. Med. [Internet]. 2012;17:171–6. Available from: http://www.ncbi.nlm.nih.gov/pubmed/22522793.

Maneiro JR, Salgado E, Gomez-Reino JJ. Immunogenicity of monoclonal antibodies against tumor necrosis factor used in chronic immune-mediated Inflammatory conditions: systematic review and meta-analysis. JAMA Intern. Med. [Internet]. 2013;173:1416–28.

Marsland a M, Chalmers RJG, Hollis S, Leonardi-Bee J, Griffiths CEM. Interventions for chronic palmoplantar pustulosis. Cochrane Database Syst. Rev. 2006;CD001433.

Mason A, Mason J, Cork M, Hancock H, Dooley G. Topical treatments for chronic plaque psoriasis: An abridged Cochrane Systematic Review. J. Am. Acad. Dermatol.2013;69:799–807.

Maybury CM, Jabbar-Lopez ZK, Wong T, Dhillon AP, Barker JN, Smith CH. Methotrexate and liver fibrosis in people with psoriasis: A systematic review of observational studies. Br. J. Dermatol. 2014;171:17–29. 21.

Maybury CM, Samarasekera E, Douiri A, Barker JN, Smith CH. Diagnostic accuracy of noninvasive markers of

liver fibrosis in patients with psoriasis taking methotrexate: A systematic review and meta-analysis. Br. J. Dermatol. 2014;170:1237–47.

Meng Y, Dongmei L, Yanbin P, Jinju F, Meile T, Binzhu L, et al. Systematic review and meta-analysis of ustekinumab for moderate to severe psoriasis. Clin. Exp. Dermatol. 2014;39:696–707.

Miller IM, Ellervik C, Yazdanyar S, Jemec GBE. Meta-analysis of psoriasis, cardiovascular disease, and associated risk factors. J. Am. Acad. Dermatol. 2013;69:1014–24.

Nast A, Jacobs A, Rosumeck S, Werner RN. Efficacy and Safety of Systemic Long-Term Treatments for Moderate-to-Severe Psoriasis: A Systematic Review and Meta-Analysis. J. Invest. Dermatol. 2015;135:2641–8.

Pickett K, Frampton G, Loveman E. Education to improve quality of life of people with chronic inflammatory skin conditions: a systematic review of the evidence. Br. J. Dermatol. 2016;174:1228–41.

Pickett K, Loveman E, Kalita N, Frampton GK, Jones J. Educational interventions to improve quality of life in people with chronic inflammatory skin diseases: Systematic reviews of clinical effectiveness and cost-effectiveness. Health Technol. Assess. 2015;19:1–95.

Roubille C, Richer V, Starnino T, McCourt C, McFarlane A, Fleming P, et al. The effects of tumour necrosis factor inhibitors, methotrexate, non-steroidal anti-inflammatory drugs and corticosteroids on cardiovascular events in rheumatoid arthritis, psoriasis and psoriatic arthritis: a systematic review and meta-analysis. Ann. Rheum. Dis. 2015;74:480–9.

Ryan C, Leonardi CL, Krueger JG, Kimball AB, Gordon KB, Langley RG, et al. Association Between Biologic Therapies for Chronic Plaque Psoriasis and cardiovascular events: a meta-analysis of randomized controlled trials. JAMA 2014;306:864–71.

Samarasekera EJ, Neilson JM, Warren RB, Parnham J, Smith CH. Incidence of cardiovascular disease in individuals with psoriasis: a systematic review and meta-analysis. J. Invest. Dermatol. 2013;133:2340–6.

Shelton E, Laharie D, Scott FI, Mamtani R, Lewis JD, Colombel JF, et al. Cancer Recurrence Following Immune-Suppressive Therapies in Patients With Immune-Mediated Diseases: A Systematic Review and Meta-analysis. Gastroenterology 2016;151:97–109.e4.

Tzellos T, Kyrgidis A, Zouboulis CC. Re-evaluation of the risk for major adverse cardiovascular events in

patients treated with anti-IL-12/23 biological agents for chronic plaque psoriasis: A meta-analysis of randomized controlled trials. J. Eur. Acad. Dermatology Venereol. 2013;27:622–7.

Warren RB, Brown BC, Griffiths CEM. Topical treatments for scalp psoriasis. Drugs 2008;68:2293–302.

Woolacott N, Hawkins N, Mason A, Kainth A, Khadjesari Z, Vergel YB, et al. Etanercept and efalizumab for the treatment of psoriasis: a systematic review. Health Technol. Assess. 2006.

Zhang CS, Yang L, Zhang AL, May BH, Yu JJ, Guo X, et al. Is Oral Chinese Herbal Medicine Beneficial for Psoriasis Vulgaris? A Meta-Analysis of Comparisons with Acitretin. J. Altern. Complement. Med. 2016;22:174–88.

Zhang CS, Yu JJ, Parker S, Zhang AL, May B, Lu C, et al. Oral Chinese herbal medicine combined with pharmacotherapy for psoriasis vulgaris: A systematic review. Int. J. Dermatol. 2014;53:1305–18.

Zhao YE, Ma JX, Hu L, Xiao SX, Zhao YL. Meta-analysis of the association between psoriasis and human leucocyte antigen-B. Br. J. Dermatol. 2013;169:417–27.

Moderate quality

Almutawa F, Alnomair N, Wang Y, Hamzavi I, Lim HW. Systematic review of UV-based therapy for psoriasis. Am. J. Clin. Dermatol. 2013;14:87–109.

Almutawa F, Thalib L, Hekman D, Sun Q, Hamzavi I, Lim HW. Efficacy of localized phototherapy and photodynamic therapy for psoriasis: A systematic review and meta-analysis. Photodermatol. Photoimmunol. Photomed. 2015;31:5–14.

Archier E, Devaux S, Castela E, Gallini A, Aubin F, Le Ma??tre M, et al. Efficacy of Psoralen UV-A therapy vs. Narrowband UV-B therapy in chronic plaque psoriasis: A systematic literature review. J. Eur. Acad. Dermatology Venereol. 2012;26:11–21.

Archier E, Devaux S, Castela E, Gallini A, Aubin F, Le Ma??tre M, et al. Carcinogenic risks of Psoralen UV-A therapy and Narrowband UV-B therapy in chronic plaque psoriasis: A systematic literature review. J. Eur. Acad. Dermatology Venereol. 2012;26:22–31.

Armstrong AW, Harskamp CT, Dhillon JS, Armstrong EJ. Psoriasis and smoking: A systematic review and meta-analysis. Br. J. Dermatol. 2014;170:304–14.

Armstrong AW, Harskamp CT, Armstrong EJ. The association between psoriasis and hypertension: a systematic review and meta-analysis of observational studies. J. Hypertens. [Internet]. 2013;31:433-42-3.

Armstrong AW, Harskamp CT, Armstrong EJ. Psoriasis and the Risk of Diabetes Mellitus. JAMA Dermatology 2013;149:84.

Armstrong EJ, Harskamp CT, Armstrong AW. Psoriasis and major adverse cardiovascular events: a systematic review and meta-analysis of observational studies. J. Am. Heart Assoc. 2013;2.

Ashcroft DM, Li Wan Po A, Williams HC, Griffiths CE. Combination regimens of topical calcipotriene in chronic plaque psoriasis: systematic review of efficacy and tolerability. Arch. Dermatol. 2000;136:1536–43.

Ashcroft DM, Li A, Po W, Williams HC, Griffiths CEM. Systematic review of comparative efficacy and tolerability of calcipotriol in treating chronic plaque psoriasis. Br. Med. J. 2000;320:963–7.

Bansback N, Sizto S, Sun H, Feldman S, Willian MK, Anis A. Efficacy of systemic treatments for moderate to severe plaque psoriasis: Systematic review and meta-analysis. Dermatology. 2009;219:209–18.

Bottomley JM, Taylor RS, Ryttov J. The effectiveness of two-compound formulation calcipotriol and betamethasone dipropionate gel in the treatment of moderately severe scalp psoriasis: a systematic review of direct and indirect evidence. Curr. Med. Res. Opin. [Internet]. 2011;27:251–68.

Brimhall AK, King LN, Licciardone JC, Jacobe H, Menter A. Safety and efficacy of alefacept, efalizumab, etanercept and infliximab in treating moderate to severe plaque psoriasis: A meta-analysis of randomized controlled trials. Br. J. Dermatol. 2008;159:274–85.

Burden-Teh E, Thomas KS, Ratib S, Grindlay D, Adaji E, Murphy R. The epidemiology of childhood psoriasis: a scoping review. Br. J. Dermatol. 2016;174:1242–57.

Busard C, Zweegers J, Limpens J, Langendam M, Spuls PI. Combined use of systemic agents for psoriasis: a systematic review. JAMA dermatology. 2014;150:1213–20.

Castela E, Archier E, Devaux S, Gallini A, Aractingi S, Cribier B, et al. Topical corticosteroids in plaque psoriasis:

A systematic review of efficacy and treatment modalities. J. Eur. Acad. Dermatology Venereol. 2012;26:36–46.

Chen H-Q, Li X, Tang R. Effects of Narrow Band Ultraviolet B on Serum Levels of Vascular Endothelial Growth Factor and Interleukin-8 in Patients with Psoriasis. Am. J. Ther. 2016;23:e655–62.

Chen Y, Qian T, Zhang D, Yan H, Hao F. Clinical efficacy and safety of anti-IL-17 agents for the treatment of patients with psoriasis. Immunotherapy. 2015;7:1023–37.

Cobo-Ibáñez T, Villaverde V, Seoane-Mato D, Muñoz-Fernández S, Guerra M, del Campo PD, et al. Multidisciplinary dermatology–rheumatology management for patients with moderate-to-severe psoriasis and psoriatic arthritis: a systematic review. Rheumatol. Int.; 2016;36:221–9.

Conway R, Low C, Coughlan RJ, O'Donnell MJ, Carey JJ. Methotrexate use and risk of lung disease in psoriasis, psoriatic arthritis, and inflammatory bowel disease: systematic literature review and meta-analysis of randomised controlled trials. Bmj 2015;350:h1269.

Correr CJ, Rotta I, Teles TDS, Godoy RR, Riveros BS, Garcia MM, et al. Efficacy and safety of biologics in the treatment of moderate to severe psoriasis: a comprehensive meta-analysis of randomized controlled trials. Cad. Saude Publica. 2013;29:s17–31.

Coyle M, Deng J, Zhang AL, Yu J, Guo X, Xue CC, et al. Acupuncture therapies for psoriasis vulgaris: a systematic review of randomized controlled trials. Forschende Komplementärmedizin (2006) 2015;22:102–9.

Dannepond C, Maruani A, Machet L, Ternant D, Paintaud G, Samimi M. Serum infliximab concentrations in psoriatic patients treated with infliximab: A systematic review. Acta Derm. Venereol. 2015;95:401–6.

de Jager MEA, de Jong EMGJ, van de Kerkhof PCM, Seyger MMB. Efficacy and safety of treatments for childhood psoriasis: A systematic literature review. J. Am. Acad. Dermatol. ; 2010;62:1013–30.

Deng S, May BH, Zhang AL, Lu C, Xue CCL. Topical herbal medicine combined with pharmacotherapy for psoriasis: A systematic review and meta-analysis. Arch. Dermatol. Res. 2013;305:179–89.

Deng S, May BH, Zhang AL, Lu C, Xue CCL. Topical herbal formulae in the management of psoriasis: Systematic review with meta-analysis of clinical studies and investigation of the pharmacological actions of the main herbs. Phyther. Res. 2014;28:480–97.

Depont F, Berenbaum F, Filippi J, Le Maitre M, Nataf H, Paul C, et al. Interventions to Improve Adherence in Patients with Immune-Mediated Inflammatory Disorders: A Systematic Review. PLoS One 2015;10:e0145076.

Fang N, Jiang M, Fan Y. Association Between Psoriasis and Subclinical Atherosclerosis: A Meta-Analysis. Medicine (Baltimore).2016;95:e3576.

Ference EH. Combination Treatments for Psoriasis. Arch. Dermatol. 2012;148:511.

Fleming P, Roubille C, Richer V, Starnino T, McCourt C, McFarlane A, et al. Effect of biologics on depressive symptoms in patients with psoriasis: A systematic review. J. Eur. Acad. Dermatology Venereol. 2015;29:1063–70.

Fleming P, Kraft J, Gulliver WP, Lynde C. The Relationship of Obesity With the Severity of Psoriasis: A Systematic Review. J. Cutan. Med. Surg.2015;19:450–6.

Garces S, Demengeot J, Benito-Garcia E. The immunogenicity of anti-TNF therapy in immune-mediated inflammatory diseases: a systematic review of the literature with a meta-analysis. Ann Rheum Dist]. 2012;annrheumdis-2012-202220-.

Generali E, Scire CA, Favalli EG, Selmi C. Biomarkers in psoriatic arthritis: a systematic literature review. Expert Rev. Clin. Immunol. 2016;8409:1–10.

Gupta AK, Daigle D, Lyons DCA. Network meta-analysis of treatments for chronic plaque psoriasis in Canada. J. Cutan. Med. Surg. 2014;18:371–8.

Gupta MA, Simpson FC, Gupta AK. Psoriasis and sleep disorders: A systematic review. Sleep Med. Rev. 2016;29:63–75.

Hamilton MP, Ntais D, Griffiths CEM, Davies LM. Psoriasis treatment and management - A systematic review of full economic evaluations. Br. J. Dermatol. 2015;172:574–83.

Han Y, Liu T, Lu L. Apolipoprotein E Gene Polymorphism in Psoriasis: A Meta-analysis. Arch. Med. Res. [Internet]. Elsevier Inc; 2013;44:46–53.

Hendriks AGM, Keijsers RRMC, De Jong EMGJ, Seyger MMB, Van De Kerkhof PCM. Efficacy and safety of combinations of first-line topical treatments in chronic plaque psoriasis: A systematic literature review. J. Eur.

Acad. Dermatology Venereol. 2013;27:931–51.

Henry AL, Kyle SD, Bhandari S, Chisholm A, Griffiths CEM, Bundy C. Measurement, Classification and Evaluation of Sleep Disturbance in Psoriasis: A Systematic Review. PLoS One. 2016;11:1–18.

Herman SM, Shin MH, Holbrook A, Rosenthal D. The role of antimalarials in the exacerbation of psoriasis: A systematic review. Am. J. Clin. Dermatol. 2006;7:249–57.

Heydendael VMR, Spuls PI, Ten Berge IJM, Opmeer BC, Bos JD, De Rie MA. Cyclosporin trough levels: Is monitoring necessary during short-term treatment in psoriasis? A systematic review and clinical data on trough levels. Br. J. Dermatol. 2002;147:122–9.

Horreau C, Pouplard C, Brenaut E, Barnetche T, Misery L, Cribier B, et al. Cardiovascular morbidity and mortality in psoriasis and psoriatic arthritis: A systematic literature review. J. Eur. Acad. Dermatology Venereol. 2013;27:12–29.

Hsu L, Snodgrass BT, Armstrong AW. Antidrug antibodies in psoriasis: A systematic review. Br. J. Dermatol. 2014;170:261–73.

Huang H, Shen E, Tang S, Tan X, Guo X, Wang Q, et al. Increased serum resistin levels correlate with psoriasis: a meta-analysis. Lipids Health Dis. 2015;14:44. A

Jia Y, Qin HJ, Zhang JX, Liu XL, Li LJ. Association of the tumour necrosis factor-α polymorphisms rs361525 and rs1800629 with susceptibility to psoriasis: A meta-analysis. Clin. Exp. Dermatol. 2013;38:836–44.

Klaassen KMG, Dulak MG, Van De Kerkhof PCM, Pasch MC. The prevalence of onychomycosis in psoriatic patients: A systematic review. J. Eur. Acad. Dermatology Venereol. 2014;28:533–41.

Lamel SA, Myer KA, Younes N, Zhou JA, Maibach H, Maibach HI. Placebo response in relation to clinical trial design: A systematic review and meta-analysis of randomized controlled trials for determining biologic efficacy in psoriasis treatment. Arch. Dermatol. Res. 2012;304:707–17.

Langham S, Langham J, Goertz H-P, Ratcliffe M. Large-scale, prospective, observational studies in patients with psoriasis and psoriatic arthritis: A systematic and critical review. BMC Med. Res. Methodol. BioMed Central Ltd; 2011;11:32.

Larsen MH, Hagen KB, Krogstad A-L, Aas E, Wahl AK. Limited evidence of the effects of patient education and self-management interventions in psoriasis patients: a systematic review. Patient Educ. Couns. 2014;94:158–69.

Lee YH, Song GG. Associations between interleukin-23R and interleukin-12B polymorphisms and psoriasis susceptibility: a meta-analysis. Immunol. Invest. 2013;42:726–36.

Li N, Li Y-Q, Li H-Y, Guo W, Bai Y-P. Efficacy of externally applied Chinese herbal drugs in treating psoriasis: a systematic review. Chin. J. Integr. Med. 2012;18:222–9.

Li X, Kong L, Li F, Chen C, Xu R, Wang H, et al. Association between psoriasis and chronic obstructive pulmonary disease: A systematic review and meta-analysis. PLoS One. 2015;10.

Li X, Miao X, Wang H, Wang Y, Li F, Yang Q, et al. Association of Serum Uric Acid Levels in Psoriasis: A Systematic Review and Meta-Analysis. Medicine (Baltimore). [Internet]. 2016;95:e3676.

Liang J, Zhang S, Luo Q, Li W, Tian X, Zhang F, et al. Lack of association between cytotoxic T-lymphocyte antigen-4+49A/G polymorphism and psoriasis and vitiligo: A meta-analysis of case-control studies. Gene 2015;568:196–202.

Lucka TC, Pathirana D, Sammain A, Bachmann F, Rosumeck S, Erdmann R, et al. Efficacy of systemic therapies for moderate-to-severe psoriasis: A systematic review and meta-analysis of long-term treatment. J. Eur. Acad. Dermatology Venereol. 2012;26:1331–44.

Ma C, Harskamp CT, Armstrong EJ, Armstrong AW. The association between psoriasis and dyslipidaemia: A systematic review. Br. J. Dermatol. 2013;168:486–95.

Mason J, Mason AR, Cork MJ. Topical preparations for the treatment of psoriasis: A systematic review. Br. J. Dermatol. 2002;146:351–64.

Mauskopf J, Samuel M, McBride D, Mallya UG, Feldman SR. Treatment sequencing after failure of the first biologic in cost-effectiveness models of psoriasis: A systematic review of published models and clinical practice guidelines. Pharmacoeconomics. 2014;32:395–409.

Maza A, Montaudi H, Sbidian E, Gallini A, Aractingi S, Aubin F, et al. Oral cyclosporin in psoriasis: A systematic review on treatment modalities, risk of kidney toxicity and evidence for use in non-plaque psoriasis. J. Eur.

Acad. Dermatology Venereol. 2011;25:19–27.

Miller IM, Skaaby T, Ellervik C, Jemec GB. Quantifying Cardiovascular Disease Risk Factors in Psoriasis patients - a Meta-analysis. Br J Dermatol. 2013;1180–7.

Miroddi M, Navarra M, Calapai F, Mancari F, Giofrè SV, Gangemi S, et al. Review of Clinical Pharmacology of Aloe vera L . in the Treatment of Psoriasis. 2015;655:648–55.

Miroddi M, Navarra M, Calapai F, Mancari F, Giofrè SV, Gangemi S, et al. Review of clinical pharmacology of Aloe vera L. in the treatment of psoriasis. Phyther. Res. 2015;29:648–55.

Montaudi H, Sbidian E, Paul C, Maza A, Gallini A, Aractingi S, et al. Methotrexate in psoriasis: A systematic review of treatment modalities, incidence, risk factors and monitoring of liver toxicity. J. Eur. Acad. Dermatology Venereol. 2011;25:12–8.

Naldi L, Rzany B. Chronic plaque psoriasis Skin disorders. 2004;2275–302.

Nast A, Rosumeck S, Seidenschnur K. Biosimilars: a systematic review of published and ongoing clinical trials of antipsoriatics in chronic inflammatory diseases. J. Dtsch. Dermatol. Ges. [Internet]. 2015;13:294–300.

Pasker-De Jong PCM, Wielink G, Van Der Valk PGM, Van Der Wilt G-J. Treatment with UV-B for psoriasis and nonmelanoma skin cancer: A systematic review of the literature. Arch. Dermatol. [Internet]. 1999;135:834–40.

Patel R V., Shelling ML, Prodanovich S, Federman DG, Kirsner RS. Psoriasis and vascular disease-risk factors and outcomes: A systematic review of the literature. J. Gen. Intern. Med. 2011;26:1036–49.

Paul C, Gallini A, Archier E, Castela E, Devaux S, Aractingi S, et al. Evidence-based recommendations on topical treatment and phototherapy of psoriasis: Systematic review and expert opinion of a panel of dermatologists. J. Eur. Acad. Dermatology Venereol. 2012;26:1–10.

Posso-De Los Rios CJ, Pope E, Lara-Corrales I. A systematic review of systemic medications for pustular psoriasis in pediatrics. Pediatr. Dermatol. 2014;31:430–9.

Pouplard C, Brenaut E, Horreau C, Barnetche T, Misery L, Richard MA, et al. Risk of cancer in psoriasis: A systematic review and meta-analysis of epidemiological studies. J. Eur. Acad. Dermatology Venereol. 2013;27:36–46.

Prey S, Paul C. Effect of folic or folinic acid supplementation on methotrexate-associated safety and efficacy in inflammatory disease: A systematic review. Br. J. Dermatol. 2009;160:622–8.

Prey S, Paul C, Bronsard V, Puzenat E, Gourraud PA, Aractingi S, et al. Assessment of risk of psoriatic arthritis in patients with plaque psoriasis: A systematic review of the literature. J. Eur. Acad. Dermatology Venereol. 2010;24:31–5.

Puig L, Lõpez A, Vilarrasa E, García I. Efficacy of biologics in the treatment of moderate-to-severe plaque psoriasis: A systematic review and meta-analysis of randomized controlled trials with different time points. J. Eur. Acad. Dermatology Venereol. 2014;28:1633–53.

Qi JH, Qi JH, Shi N, Chen YJ, Nie G. Association between MTHFR 677C/T polymorphism and psoriasis risk: A meta-analysis. Genet. Mol. Res. 2015;14:3869–76.

Qi M, Huang X, Zhou L, Zhang J. Four polymorphisms of VEGF (+405C>G, -460T>C, -2578C>A, and -1154G>A) in susceptibility to psoriasis: a meta-analysis. DNA Cell Biol. [Internet]. 2014;33:234–44.

Rachakonda TD, Dhillon JS, Florek AG, Armstrong AW. Effect of tonsillectomy on psoriasis: A systematic review. J. Am. Acad. Dermatol.2015;72:261–75.

Reich K, Burden AD, Eaton JN, Hawkins NS. Efficacy of biologics in the treatment of moderate to severe psoriasis: A network meta-analysis of randomized controlled trials. Br. J. Dermatol. 2012;166:179–88.

Richard MA, Barnetche T, Rouzaud M, Sevrain M, Villani AP, Aractingi S, et al. Evidence-based recommendations on the role of dermatologists in the diagnosis and management of psoriatic arthritis: Systematic review and expert opinion. J. Eur. Acad. Dermatology Venereol. 2014;28:3–12.

Richer V, Roubille C, Fleming P, Starnino T, McCourt C, McFarlane A, et al. Psoriasis and Smoking: A Systematic Literature Review and Meta-Analysis With Qualitative Analysis of Effect of Smoking on Psoriasis Severity. J. Cutan. Med. Surg.2015;20:221–7.

Roubille C, Richer V, Starnino T, McCourt C, McFarlane A, Fleming P, et al. Evidence-based recommendations for the management of comorbidities in rheumatoid arthritis, psoriasis, and psoriatic arthritis: Expert opinion of the canadian dermatology-rheumatology comorbidity initiative. J. Rheumatol. 2015;42:1767–80.

Rouzaud M, Sevrain M, Villani a P, Barnetche T, Paul C, Richard M, et al. Is there a psoriasis skin phenotype associated with psoriatic arthritis? Systematic literature review. J. Eur. Acad. Dermatol. Venereol. 2014;28 Suppl 5:17–26.

Samarasekera EJ, Sawyer L, Wonderling D, Tucker R, Smith CH. Topical therapies for the treatment of plaque psoriasis: Systematic review and network meta-analyses. Br. J. Dermatol. 2013;168:954–67.

Sbidian E, Maza A, Montaudi?? H, Gallini A, Aractingi S, Aubin F, et al. Efficacy and safety of oral retinoids in different psoriasis subtypes: A systematic literature review. J. Eur. Acad. Dermatology Venereol. 2011;25:28–33.

Schmitt J, Rosumeck S, Thomaschewski G, Sporbeck B, Haufe E, Nast A. Efficacy and safety of systemic treatments for moderate-to-severe psoriasis: Meta-analysis of randomized controlled trials. Br. J. Dermatol. 2014;170:274–303.

Schmitt J, Zhang Z, Wozel G, Meurer M, Kirch W. Efficacy and tolerability of biologic and nonbiologic systemic treatments for moderate-to-severe psoriasis: Meta-analysis of randomized controlled trials. Br. J. Dermatol. 2008;159:513–26.

Sevrain M, Richard MA, Barnetche T, Rouzaud M, Villani AP, Paul C, et al. Treatment for palmoplantar pustular psoriasis: Systematic literature review, evidence-based recommendations and expert opinion. J. Eur. Acad. Dermatology Venereol. 2014;28:13–6.

Signorovitch JE, Betts KA, Yan YS, Lereun C, Sundaram M, Wu EQ, et al. Comparative efficacy of biological treatments for moderate-to-severe psoriasis: A network meta-analysis adjusting for cross-trial differences in reference arm response. Br. J. Dermatol. 2015;172:504–12.

Souto A, Maneiro JR, Salgado E, Carmona L, Gomez-Reino JJ. Risk of tuberculosis in patients with chronic immune-mediated inflammatory diseases treated with biologics and tofacitinib: a systematic review and meta-analysis of randomized controlled trials and long-term extension studies. Rheumatology (Oxford). 2014;53:1872–85.

Spuls PI, Witkamp L, Bossuyt PM, Bos JD. A systematic review of five systemic treatments for severe psoriasis. Br. J. Dermatol. 1997;137:943–9.

Stefanic M, Rucevic I, Barisic-drusko V. Medical genetics Meta-analysis of vitamin D receptor polymorphisms

and psoriasis risk. Int. J. Dermatol. 2013;52:705–10.

Sullivan JR, Preda VA. Treatments for severe psoriasis. Aust. Prescr. 2009;32:14–8.

Svendsen MT, Andersen F, Hansen J, Johannessen H, Andersen KE. Medical adherence to topical corticosteroid preparations prescribed for psoriasis: A systematic review. J. Dermatolog. Treat. [Internet]. 2016;6634:1–8.

Tan JY, Li S, Yang K, Ma B, Chen W, Zha C, et al. Ustekinumab, a human interleukin-12/23 monoclonal antibody, in patients with psoriasis: A meta-analysis. J. Dermatolog. Treat. [Internet]. 2011;22:323–36.

Thorlund K, Druyts E, Aviña-Zubieta JA, Mills EJ. Anti-tumor necrosis factor (TNF) drugs for the treatment of psoriatic arthritis: An indirect comparison meta-analysis. Biol. Targets Ther. 2012;6:417–27.

Thorneloe RJ, Bundy C, Griffiths CEM, Ashcroft DM, Cordingley L. Adherence to medication in patients with psoriasis: A systematic literature review. Br. J. Dermatol. 2013;168:20–31.

Tott JEE, van der Feltz WT, Bode LGM, van Belkum A, van Zuuren EJ, Pasmans SGMA. A systematic review and meta-analysis on Staphylococcus aureus carriage in psoriasis, acne and rosacea. Eur. J. Clin. Microbiol. Infect. Dis. 2016;35:1069–77.

Ungprasert P, Sanguankeo A, Upala S, Suksaranjit P. Psoriasis and risk of venous thromboembolism: a systematic review and meta-analysis. QJM. 2014;107:793–7.

Ungprasert P, Srivali N, Kittanamongkolchai W. Risk of Parkinson's Disease Among Patients with Psoriasis: A Systematic Review and Meta-analysis. Indian J. Dermatol. [Internet]. 2016;61:152–6.

Upala S, Sanguankeo A. Effect of lifestyle weight loss intervention on disease severity in patients with psoriasis: a systematic review and meta-analysis. Int. J. Obes. (Lond). [Internet]. Nature Publishing Group; 2015;39:1197–202.

Van Geel MJ, Mul K, De Jager MEA, Van De Kerkhof PCM, De Jong EMGJ, Seyger MMB. Systemic treatments in paediatric psoriasis: A systematic evidence-based update. J. Eur. Acad. Dermatology Venereol. 2015;29:425–37.

Vangeli E, Bakhshi S, Baker A, Fisher A, Bucknor D, Mrowietz U, et al. A Systematic Review of Factors

Associated with Non-Adherence to Treatment for Immune-Mediated Inflammatory Diseases. Adv. Ther. 2015;32:983–1028.

illani AP, Rouzaud M, Sevrain M, Barnetche T, Paul C, Richard MA, et al. Prevalence of undiagnosed psoriatic arthritis among psoriasis patients: Systematic review and meta-analysis. J. Am. Acad. Dermatol. 2015;73:242–8.

Wang J, Zhan Q, Zhang L. A systematic review on the efficacy and safety of Infliximab in patients with psoriasis. Hum. Vaccines Immunother. [Internet]. 2016;12:431–7.

Wang X, Guo Z, Zhu Z, Bao Y. Epicardial fat tissue in patients with psoriasis: a systematic review and meta-analysis. Lipids Heal. [Internet]. Lipids in Health and Disease; 2016;15:103.

West J, Ogston S, Foerster J. Safety and efficacy of methotrexate in psoriasis: A meta-analysis of published trials. PLoS One. 2016;11:1–14.

Wilson A, Basharat P, Levstik M, Barra L. Autoimmune Diseases and Therapeutic Approaches Open Access Transient Elastography for Detecting Liver Fibrosis in Methotrexate-Treated Patients with Inflammatory Disorders : A Systematic Review. 2014;1:1–10.

Winthrop KL, Novosad S a, Baddley JW, Calabrese L, Chiller T, Polgreen P, et al. Opportunistic infections and biologic therapies in immune-mediated inflammatory diseases: consensus recommendations for infection reporting during clinical trials and postmarketing surveillance. Ann. Rheum. Dis. [Internet]. 2015;1–10.

Wu Y, Wang B, Liu JL, Gao XH, Chen HD, Li YH. Association of -619C/T polymorphism in CDSN gene and psoriasis risk: A meta-analysis. Genet. Mol. Res. 2011;10:3632–40.

Xia T, Diao J, Huang H, Li J, Sun L, Li H, et al. Evaluation of the Association Between CD143 Gene Polymorphism and Psoriasis. Cell Biochem. Biophys. 2014;70:1617–23.

Xiao J-P, Wang X-R, Zhang S, Wang H-Y, Ye L, Pan H-F, et al. Association between rs6887695 and 3′-untranslated region polymorphisms within the interleukin-12B gene and susceptibility to autoimmune diseases in Asian and European population: A meta-analysis. Autoimmunity [Internet]. 2016;49:277–84.

Xu T, Zhang YH. Association of psoriasis with stroke and myocardial infarction: Meta-analysis of cohort studies. Br. J. Dermatol. 2012;167:1345–50.

Yan R, Jiang S, Wu Y, Gao X-H, Chen H-D. Topical calcipotriol/betamethasone dipropionate for psoriasis vulgaris: A systematic review. Indian J. Dermatol. Venereol. Leprol. [Internet]. 2016;82:135–44.

Yang L, Zhang CS, May B, Yu J, Guo X, Zhang AL, et al. Efficacy of combining oral Chinese herbal medicine and NB-UVB in treating psoriasis vulgaris: a systematic review and meta-analysis. Chin. Med. [Internet]. BioMed Central; 2015;10:27.

Yang Y, Brazier J, Longworth L. EQ-5D in skin conditions: an assessment of validity and responsiveness. Eur. J. Health Econ. [Internet]. Springer Berlin Heidelberg; 2015;16:927–39.

Yang Z-S, Lin N-N, Li L, Li Y. The Effect of TNF Inhibitors on Cardiovascular Events in Psoriasis and Psoriatic Arthritis: an Updated Meta-Analysis. Clin. Rev. Allergy Immunol. [Internet]. 2016;1–8.

Yu JJ, Zhang CS, Zhang AL, May B, Xue CC, Lu C. Add-on effect of chinese herbal medicine bath to phototherapy for psoriasis vulgaris: A systematic review. Evidence-based Complement. Altern. Med. 2013;2013.

Zhang W, Islam N, Ma C, Anis AH. Systematic Review of Cost-Effectiveness Analyses of Treatments for Psoriasis. Pharmacoeconomics. 2015;33:327–40.

Zhao YE, Hu L, Ma JX, Xiao SX, Zhao YL. Investigation of the association between psoriasis and human leucocyte antigens A by means of meta-analysis. J. Eur. Acad. Dermatology Venereol. 2014;28:355–69.

Zhou D, Chen W, Li X, Deng B, Xu W, Qu J, et al. Evidence-based practice guideline of Chinese herbal medicine for psoriasis vulgaris (Bai Bi). Eur. J. Integr. Med. [Internet]. Elsevier GmbH.; 2014;6:135–46.

Zhu KJ, Zhang C, Li M, Zhu CY, Shi G, Fan YM. Leptin levels in patients with psoriasis: A meta-analysis. Clin. Exp. Dermatol. 2013;38:478–83.

Zhu KJ, Zhu CY, Fan YM. Alcohol consumption and psoriatic risk: A meta-analysis of case-control studies. J. Dermatol. 2012;39:770–3.

Zhu K-J, Shi G, Zhang C, Li M, Zhu C-Y, Fan Y-M. Adiponectin levels in patients with psoriasis: A meta-analysis. J. Dermatol. [Internet]. 2013;40:438–42. Available from: http://doi.wiley.com/10.1111/1346-8138.12121

Low quality

Ahn CS, Gustafson CJ, Sandoval LF, Davis SA, Feldman SR. Cost effectiveness of biologic therapies for plaque psoriasis. Am. J. Clin. Dermatol. 2013;14:315–26.

Archier E, Devaux S, Castela E, Gallini A, Aubin F, Le Ma??tre M, et al. Ocular damage in patients with psoriasis treated by Psoralen UV-A therapy or Narrow band UVB therapy: A systematic literature review. J. Eur. Acad. Dermatology Venereol. 2012;26:32–5.

Augustin M, Mrowietz U, Bonnekoh B, Rosenbach T, Thaçi D, Reusch M, et al. Topical long-term therapy of psoriasis with vitamin D_3 analogues, corticosteroids and their two compound formulations: position paper on evidence and use in daily practice. J. Dtsch. Dermatol. Ges. [Internet]. 2014;12:667–82.

Boehncke W-H, Alvarez Martinez D, Solomon J, Gottlieb AB. Safety and efficacy of therapies for skin symptoms of psoriasis in patients with psoriatic arthritis: a systematic review. J. Rheumatol. [Internet]. 2014;41:2301–5. A

Brenaut E, Horreau C, Pouplard C, Barnetche T, Paul C, Richard MA, et al. Alcohol consumption and psoriasis: A systematic literature review. J. Eur. Acad. Dermatology Venereol. 2013;27:30–5.

Brezinski EA, Armstrong AW. Off-label biologic regimens in psoriasis: A systematic review of efficacy and safety of dose escalation, reduction, and interrupted biologic therapy. PLoS One. 2012;7:1–10.

Bronsard V, Paul C, Prey S, Puzenat E, Gourraud PA, Aractingi S, et al. What are the best outcome measures for assessing quality of life in plaque type psoriasis? A systematic review of the literature. J. Eur. Acad. Dermatology Venereol. 2010;24:17–22.

C.M. O, R.J. C, T. O, C.E. G. Antistreptococcal interventions for guttate and chronic plaque psoriasis. Cochrane Database Syst. Rev. 2000;CD001976.

Cantini F, Boccia S, Goletti D, Iannone F, Leoncini E, Panic N, et al. HBV reactivation in patients treated with antitumor necrosis factor-alpha (TNF- ??) agents for rheumatic and dermatologic conditions: A systematic review and meta-analysis. Int. J. Rheumatol 2014;2014.

Castela E, Archier E, Devaux S, Gallini A, Aractingi S, Cribier B, et al. Topical corticosteroids in plaque psoriasis:

A systematic review of risk of adrenal axis suppression and skin atrophy. J. Eur. Acad. Dermatology Venereol. 2012;26:47–51.

Chalmers RJG, O'Sullivan T, Owen CM, Griffiths CEM. A systematic review of treatments for guttate psoriasis. Br. J. Dermatol. 2001;145:891–4.

Collamer AN, Battafarano DF. Psoriatic Skin Lesions Induced by Tumor Necrosis Factor Antagonist Therapy: Clinical Features and Possible Immunopathogenesis. Semin. Arthritis Rheum. [Internet]. Elsevier Inc.; 2010;40:233–40.

Consuelo V David, Sandy Chira, Samantha J Eells, Manasi Ladrigan, Art Papier, Loren G Miller, Noah Craft. A systematic review of adverse effects associated with topical treatments for psoriasis. Dermatol Online J. 2003 Feb;9(1):2.

Devaux S, Castela A, Archier E, Gallini A, Joly P, Misery L, et al. Topical vitamin D analogues alone or in association with topical steroids for psoriasis: a systematic review. J. Eur. Acad. Dermatology Venereol. JEADV [Internet]. 2012;26 Suppl 3:52–60.

Devaux S, Castela A, Archier E, Gallini A, Joly P, Misery L, et al. Adherence to topical treatment in psoriasis: A systematic literature review. J. Eur. Acad. Dermatology Venereol. 2012;26:61–7.

Erceg A, De Jong EMJG, Van De Kerkhof PCM, Seyger MMB. The efficacy of pulsed dye laser treatment for inflammatory skin diseases: A systematic review. J. Am. Acad. Dermatol. [Internet]. Elsevier Inc; 2013;69:609–615.e8.

Feldman SR, Burudpakdee C, Gala S, Nanavaty M, Mallya UG. The economic burden of psoriasis: a systematic literature review. Expert Rev Pharmacoecon Outcomes Res. 2014;14:685–705.

Feldman SR, Yentzer BA. Topical clobetasol propionate in the treatment of psoriasis: A review of newer formulations. Am. J. Clin. Dermatol. 2009;10:397–406.

Ferreira BIRC, Da Costa Abreu JLP, Dos Reis JPG, Da Costa Figueiredo AM. Psoriasis and associated psychiatric disorders: A systematic review on etiopathogenesis and clinical correlation. J. Clin. Aesthet. Dermatol. 2016;9:36–43.

Franco-Aguirre JQ, Cardona-Arias JA. Characterization of health-related quality of life studies in people with

psoriasis: Systematic review 2003-2013. Rev. Colomb. Reumatol. 2014;21:35–41.

Galván-Banqueri M, Marín Gil R, Santos Ramos B, Bautista Paloma FJ. Biological treatments for moderate-to-severe psoriasis: Indirect comparison. J. Clin. Pharm. Ther. 2013;38:121–30.

Garduno J, Bhosle MJ, Balkrishnan R, Feldman SR. Measures used in specifying psoriasis lesion(s), global disease and quality of life: a systematic review. J. Dermatolog. Treat. [Internet]. 2007;18:223–42.

Hendriks AGM, Keijsers RRMC, De Jong EMGJ, Seyger MMB, Van De Kerkhof PCM. Combinations of classical time-honoured topicals in plaque psoriasis: A systematic review. J. Eur. Acad. Dermatology Venereol. 2013;27:399–410.

Jacobi A, Mayer A, Augustin M. Keratolytics and Emollients and Their Role in the Therapy of Psoriasis: a Systematic Review. Dermatol. Ther. (Heidelb). 2015;5:1–18.

Kim IH, West CE, Kwatra SG, Feldman SR, O'Neill JL. Comparative efficacy of biologics in psoriasis: A review. Am. J. Clin. Dermatol. 2012;13:365–74.

Kitchen H, Cordingley L, Young H, Griffiths CEM, Bundy C. Patient-reported outcome measures in psoriasis: The good, the bad and the missing! Br. J. Dermatol. 2015;172:1210–21.

Kurizky PS, Maria L. Sexual dysfunction in patients with psoriasis and psoriatic arthritis – a systematic review. 2012;52.

L.F. S, B. W, S.R. F. Clinical potential of brodalumab in the management of psoriasis: The evidence to date. Psoriasis Targets Ther. [Internet]. 2015;5:35–41.

Lee YH, Song GG. Vascular endothelial growth factor gene polymorphisms and psoriasis susceptibility: a meta-analysis. Genet. Mol. Res. [Internet]. 2015;14:14396–405.

Mattei PL, Corey KC, Kimball AB. Psoriasis Area Severity Index (PASI) and the Dermatology Life Quality Index (DLQI): The correlation between disease severity and psychological burden in patients treated with biological therapies. J. Eur. Acad. Dermatology Venereol. 2014;28:333–7.

Meeuwis KAP, de Hullu JA, Massuger LFAG, van de Kerkhof PCM, van Rossum MM. Genital psoriasis: A systematic literature review on this hidden skin disease. Acta Derm. Venereol. 2011;91:5–11.

Mezentsev A V., Bruskin SA, Soboleva AG, Sobolev V V., Piruzian ES. Pharmacological control of receptor of ddvanced glycation end-products and its biological effects in psoriasis. Int. J. Biomed. Sci. 2013;9:112–22.

Molina-Leyva A, Jiménez-Moleõn JJ, Naranjo-Sintes R, Ruiz-Carrascosa JC. Sexual dysfunction in psoriasis: A systematic review. J. Eur. Acad. Dermatology Venereol. 2015;29:649–55.

Mosca S, Gargiulo P, Balato N, Di Costanzo L, Parente A, Paolillo S, et al. Ischemic cardiovascular involvement in psoriasis: A systematic review. Int. J. Cardiol. [Internet]. Elsevier Ireland Ltd; 2015;178:191–9.

Parisi R, Symmons DPM, Griffiths CEM, Ashcroft DM. Global Epidemiology of Psoriasis: A Systematic Review of Incidence and Prevalence. J. Invest. Dermatol. [Internet].; 2012;133:377–85.

Posso-De Los Rios CJ, Pope E, Lara-Corrales I. A systematic review of systemic medications for pustular psoriasis in pediatrics. Pediatr. Dermatol. 2014;31:430–9.

Prey S, Paul C, Bronsard V, Puzenat E, Gourraud P-A, Aractingi S, et al. Cardiovascular risk factors in patients with plaque psoriasis: a systematic review of epidemiological studies. J. Eur. Acad. Dermatology Venereol. JEADV [Internet]. 2010;24 Suppl 2:23–30.

Richard MA, Barnetche T, Horreau C, Brenaut E, Pouplard C, Aractingi S, et al. Psoriasis, cardiovascular events, cancer risk and alcohol use: Evidence-based recommendations based on systematic review and expert opinion. J. Eur. Acad. Dermatology Venereol. 2013;27:2–11.

Robinson A, Kardos M, Kimball AB. Physician Global Assessment (PGA) and Psoriasis Area and Severity Index (PASI): Why do both? A systematic analysis of randomized controlled trials of biologic agents for moderate to severe plaque psoriasis. J. Am. Acad. Dermatol. [Internet]. Elsevier Inc; 2012;66:369–75.

Ryoo JY, Yang HJ, Ji E, Yoo BK. Meta-analysis of the Efficacy and Safety of Secukinumab for the Treatment of Plaque Psoriasis. Ann. Pharmacother. 2016;50:341–51.

Sandoval LF, Pierce A, Feldman SR. Systemic therapies for psoriasis: An evidence-based update. Am. J. Clin. Dermatol. 2014;15:165–80.

Shaharyar S, Warraich H, McEvoy JW, Oni E, Ali SS, Karim A, et al. Subclinical cardiovascular disease in plaque psoriasis: Association or causal link?? Atherosclerosis [Internet]. Elsevier Ltd; 2014;232:72–8.

Shen C, Gao J, Yin X, Sheng Y, Sun L, Cui Y, et al. Association of the late cornified envelope-3 genes with psoriasis and psoriatic arthritis: A systematic review. J. Genet. Genomics. 2015;42:49–56.

Shenoy C, Shenoy MM, Rao GK. Dyslipidemia in dermatological disorders. N. Am. J. Med. Sci. 2015;7:421–8.

Simonart T, Heenen M, Lejeune O. Epidermal kinetic alterations required to generate the psoriatic phenotype: A reappraisal. Cell Prolif. 2010;43:321–5.

Smith N, Weymann A, Tausk FA, Gelfand JM. Complementary and alternative medicine for psoriasis: A qualitative review of the clinical trial literature. J. Am. Acad. Dermatol.2009;61:841–56.

Spuls PI, Lecluse LLA, Poulsen M-LNF, Bos JD, Stern RS, Nijsten T. How good are clinical severity and outcome measures for psoriasis?: quantitative evaluation in a systematic review. J. Invest. Dermatol.2010;130:933–43.

Strohal R, Chimenti S, Vena GA, Girolomoni G. Etanercept provides an effective, safe and flexible short- and long-term treatment regimen for moderate-to-severe psoriasis: a systematic review of current evidence. J. Dermatolog. Treat. [Internet]. 2013;24:199–208.

Teixeira A, Teixeira M, Almeida V, Torres T, Sousa Lobo JM, Almeida IF. Methodologies for medication adherence evaluation: Focus on psoriasis topical treatment. J. Dermatol. Sci.; 2016;82:63–8.

Umar N, Yamamoto S, Loerbroks A, Terris D. Elicitation and use of patients' preferences in the treatment of psoriasis: A systematic review. Acta Derm. Venereol. 2012;92:341–6. 53.

Villani AP, Rouzaud M, Sevrain M, Barnetche T, Paul C, Richard MA, et al. Symptoms dermatologists should look for in daily practice to improve detection of psoriatic arthritis in psoriasis patients: An expert group consensus. J. Eur. Acad. Dermatology Venereol. 2014;28:27–32.

Winthrop KL, Novosad S a, Baddley JW, Calabrese L, Chiller T, Polgreen P, et al. Opportunistic infections and biologic therapies in immune-mediated inflammatory diseases: consensus recommendations for infection reporting during clinical trials and postmarketing surveillance. Ann. Rheum. Dis. 2015;1–10.

Yamauchi PS, Bissonnette R, Teixeira HD, Valdecantos WC. Systematic review of efficacy of anti–tumor necrosis factor (TNF) therapy in patients with psoriasis previously treated with a different anti–TNF agent. J. Am.

Acad. Dermatol; 2016;75:612–618.e6.

Zhang CS, May B, Yan Y, Yu JJ, Yao D, Chang S, et al. Terms referring to psoriasis vulgaris in the classical Chinese medicine literature: a systematic analysis. Complement. Ther. Med.2016;25:55–60.

Zhu KJ, Zhu CY, Shi G, Fan YM. Meta-analysis of IL12B polymorphisms (rs3212227, rs6887695) with psoriasis and psoriatic arthritis. Rheumatol. Int. 2013;33:1785–90.

Table C. List of non included studies.

2016

Elyoussfi S.,Thomas B.J.,Ciurtin C Tailored treatment options for patients with psoriatic arthritis and psoriasis: review of established and new biologic and small molecule therapies.Rheumatology International (2016) **36:** (603-612).

Hanley T.,Handford M.,Lavery D et al.Assessment and monitoring of biologic drug adverse events in patients with psoriasis.Psoriasis: Targets and Therapy (2016) 6 (41-54).

Farahnik B.,Beroukhim K.,Zhu T.H. et al.Ixekizumab for the Treatment of Psoriasis: A Review of Phase III Trials. Dermatology and Therapy (2016) 6 (25-37).

Zhu T.H,Nakamura M.,Abrouk M et al.Demyelinating disorders secondary to TNF-inhibitor therapy for the treatment of psoriasis: A review. Journal of Dermatological Treatment (2016) (1-8).

Ling T.C,Clayton T.H.,Crawley J. et al. British Association of Dermatologists and British Photodermatology Group guidelines for the safe and effective use of psoralen-ultraviolet A therapy 2015. British Journal of Dermatology (2016) **174:** (24-55).

Chiricozzi A.,Suarez-Farias M.,Fuentes-Duculan J et al. Increased expression of interleukin-17 pathway genes in nonlesional skin of moderate-to-severe psoriasis vulgaris. British Journal of Dermatology (2016) 174:(136-145).

Hinde S., Wade R., Palmer S., Woolacott N., Spackman E. Apremilast for the Treatment of Moderate to Severe Plaque Psoriasis: A Critique of the Evidence. PharmacoEconomics (2016) **34**: (587-596).

Dowlatshahi E.A., Wakkee M. Rising interest in the field of paediatric psoriasis. British Journal of Dermatology (2016) **174**: (1180-1181).

Harden J.L, Lewis S.M., Lish S.R., Suarez-Fariñas M., Gareau D., Lentini T., Johnson-Huang L.M., Krueger J.G., Lowes M.A. The tryptophan metabolism enzyme L-kynureninase is a novel inflammatory factor in psoriasis and other inflammatory diseases. Journal of Allergy and Clinical Immunology (2016) **137**:(1830-1840).

Farahnik B., Beroukhim K., Abrouk M., Nakamura M., Zhu T.H., Singh R, Lee K., Bhutani T., Koo J. Brodalumab for the Treatment of Psoriasis: A Review of Phase III Trial. Dermatology and Therapy (2016) **6**: (111-124).

Brambilla L.,Brena M.,Tourlaki. Textiles in dermatology: Our experience and literature review. Giornale Italiano di Dermatologia e Venereologia (2016) **151**:(266-274).

Romero-Jimenez R.M, Escudero-Vilaplana V., Baniandres Rodriguez O, Garcia-Gonzalez X., Sanjurjo Saez M. Efficiency of biological therapies in patients with moderate to severe psoriasis: Impact of a pharmacotherapeutic protocol. Journal of Dermatological Treatment (2016) **27**: (198-202).

Jackson C., Maibach H. Ethnic and socioeconomic disparities in dermatology. Journal of Dermatological Treatment (2016) **27**:(290-291).

Izzo A.A.,Hoon-Kim S.,Radhakrishnan R.,Williamson E.M.A Critical Approach to Evaluating Clinical Efficacy, Adverse Events and Drug Interactions of Herbal Remedies. Phytotherapy Research (2016) **30**: (691-700).

Walia H.K.,Mehra R.Overview of common sleep disorders and intersection with dermatologic conditions.International Journal of Molecular Sciences (2016) **17**: Article Number: 654.

Tencer T.,Clancy Z.Zhang F.Cost per responder of apremilast and etanercept in patients with moderate to severe plaque psoriasis using results from liberate.Journal of the American Academy of Dermatology (2016) **74**:SUPPL. 1 (AB242).

Mughal F.,Barker J.,Cawston H.,Damera V.,Bewley A.,Morris J.,Shaw T.,Tencer T.,Zhang F. Cost-effectiveness of apremilast in moderate to severe psoriasis in the UK.Journal of the American Academy of Dermatology (2016) **74**:SUPPL. 1 (AB243).

Gomez-Garcia, F. Epstein D.,Ruano J.Network metaanalysis of new biologic agents targeting IL-23/Th17 pathway for moderate to severe plaque psoriasis.Journal of the American Academy of Dermatology (2016) **74**:SUPPL. 1 (AB261).

Petto H.,Wilhelm S.,Mallbris L,Dutronc Y,Leonardi C.,Warren R.   A statistical tool to convert published PASI 75, PASI 90 and PASI 100 response rates into absolute PASI values.Journal of the American Academy of Dermatology (2016) **74**: SUPPL. 1 (AB234).

Ma K.,Zhang H.,Baloch Z. Pathogenetic and therapeutic applications of tumor necrosis factor-Î± (TNF-Î±) in major depressive disorder: A systematic review.International Journal of Molecular Sciences (2016) **17**:Article Number: 733.

Haraoui B.Limitations in the full reporting of systematic literature review.Journal of Rheumatology (2016) **43**: (994).

Orbai A.-M.,Ogdie A. Patient-Reported Outcomes in Psoriatic Arthritis.Rheumatic Disease Clinics of North America (2016) **42**: (265-283).

Radtke M.A.,Spehr C.,Reich K.,Rustenbach S.J,Feuerhahn J,Augustin M.Treatment Satisfaction in Psoriasis: Development and Use of the PsoSat Patient Questionnaire in a Cross-Sectional Study.Dermatology (2016).

Karczewski J.,Dobrowolska A.,Rychlewska-HaÅ„czewska A.,Adamski Z.New insights into the role of T cells in pathogenesis of psoriasis and psoriatic arthritis.Autoimmunity (2016) (1-16).

Nyssen O.P.,Taylor S.J.C.,Wong G.,Steed E.,Bourke L.,Lord J.,Ross C.A.,Hayman S.,Field V.Higgins A.,Greenhalgh T.,Meads C.Does therapeutic writing help people with long-term conditions? Systematic review, realist synthesis and economic considerations. Health Technology Assessment (2016) **20**: (1-367).

Bull L.,Rayment M.HIV-indicator-condition-driven HIV testing: Clinically effective but still rarely implemented.Clinical Medicine, Journal of the Royal College of Physicians of London (2016) **16**:(175-179)

Carrascosa J.M., De La Cueva P., Ara M., Puig L., Bordas X., Carretero G., FerrÃ¡ndiz L., SÃnchez-Carazo J.L., Dauden E., Lopez-Estebaranz J.L., Vidal D., Herranz P., Jorquera E., Coto-Segura P., Ribera M. Methotrexate in Moderate to Severe Psoriasis: Review of the Literature and Expert Recommendations. Actas Dermo-Sifiliograficas (2016) **107:**(194-206).

Wong I.T.Y., Shojania K., Dutz J., Tsao N.W. Clinical and economic review of secukinumab for moderate-to-severe plaque psoriasis.Expert Review of Pharmacoeconomics and Outcomes Research (2016) **16**:(153-166).

Islam P.S.,Chang C.,Selmi C.,Generali E.,Huntley A.,Teuber S.S,Gershwin M.E. Medical Complications of Tattoos: A Comprehensive Review.Clinical Reviews in Allergy and Immunology (2016) (1-14).

Bahrani E.,Nunneley C.E.,Hsu S.,Kass J.S.Cutaneous Adverse Effects of Neurologic Medications.CNS Drugs (2016) **30**: (245-267).

Kalra S.,Gupta Y. Endocrine and metabolic effects of glucagon like peptide 1 receptor agonists (GLP1RA).Journal of the Pakistan Medical Association (2016) **66:**(357-359).

Ramiro S.,Smolen J.S.,LandewÃ R.,Van Der Heijde D.,Dougados M.,Emery P.,De Wit M.,Cutolo M.Oliver S.,Gossec L. Pharmacological treatment of psoriatic arthritis: A systematic literature review for the 2015 update of the EULAR recommendations for the management of psoriatic arthritis. Annals of the Rheumatic Diseases (2016) **75**:(490-498).

Rosenblat J.D.,McIntyre R.S.Bipolar Disorder and Inflammation.Psychiatric Clinics of North America (2016) **39:** (125-137).

Salavastru C.M. Approach to the Evaluation and Management of Drug Eruptions
Current Dermatology Reports (2016) **5**: (49-57).

Gossec L.,Smolen J.S.,Ramiro S et al. European League Against Rheumatism (EULAR) recommendations for the management of psoriatic arthritis with pharmacological therapies: 2015 update. Annals of the Rheumatic Diseases (2016) **75**:(499-510).

Generali E.,ScirÃ¨ C.A,Cantarini L.,Selmi C.Sex differences in the treatment of Psoriatic Arthritis: A systematic literature review.Israel Medical Association Journal (2016) **18**:(203-208).

Mantovani A.,Gisondi P,Lonardo A.,Targher G.Relationship between non-alcoholic fatty liver disease and psoriasis: A novel Hepato-Dermal axis?. International Journal of Molecular Sciences (2016) **17**:2.

Atzmony L.,Reiter O.,Hodak E.,Gdalevich M.,Mimouni D.Treatments for Cutaneous Lichen Planus: A Systematic Review and Meta-Analysis.American Journal of Clinical Dermatology (2016) **17**: (11-22).

Checchio T.,Ito K.,Mandema J et al.A model based meta-analysis (MBMA) for comparative efficacy of psoriasis treatments: Dose-response model for psoriasis area and severity index (PASI) responses.Clinical Pharmacology and Therapeutics (2016) **99** SUPPL. 1 (S44-S45).

Ahadieh S.,Ito K.,Wolk R.Valdez H.,Tallman A.,Krishnaswami S.,Gupta P. Model-based meta-analysis (MBMA) for evaluating time course of clinical response across psoriasis treatments.Clinical Pharmacology and Therapeutics (2016) **99** SUPPL. 1 (S52).

Taylor W.J. St.clair E.W.Editorial: Shifting the Goal Posts: Treatment Recommendations for Ankylosing Spondylitis and the Newly Defined Condition of Nonradiographic Axial Spondyloarthritis.Arthritis and Rheumatology (2016) **68:** (265-269).

Letter from the Editor.Human Vaccines and Immunotherapeutics (2016) **12**:(255).

Lv Z.,Fan J.,Zhang X.,Huang Q.,Han J.,Wu F.,Hu G.,Guo M.,Jin Y.Integrative genomic analysis of interleukin-36RN and its prognostic value in cancer.Molecular Medicine Reports (2016) **13**: (1404-1412).

Korman A.M.,Hill D.,Alikhan A.,Feldman S.R.Impact and management of depression in psoriasis patients.Expert Opinion on Pharmacotherapy (2016) **17**:(147-152).

Landriscina A.,Friedman A.J.Integrating lifestyle-focused approaches into psoriasis care: Improving patient outcomes?. Psoriasis: Targets and Therapy (2016) **6** (1-5).

Rothstein B.,Gottlieb A.Secukinumab for treating plaque psoriasis. Expert Opinion on Biological Therapy (2016) **16:** (119-128).

Wiseman S.J.,Ralston S.H.,Wardlaw J.M. Cerebrovascular disease in rheumatic diseases a systematic review

and meta-analysis.Stroke (2016) 47:4 (943-950).

Chen Y.,Sun J.,Yang Y.Huang Y.,Liu G.Malignancy risk of anti-tumor necrosis factor alpha blockers: an overview of systematic reviews and meta-analyses.Clinical Rheumatology (2016) **35**: (1-18).

Chen S.-W.,Zhong X.-S.,Jiang L.-N.,Zheng X.-Y.,Xiong Y.-Q.,Ma S.-J.,Qiu M.,Huo S.-T.,Ge J.Chen Q. Maternal autoimmune diseases and the risk of autism spectrum disorders in offspring: A systematic review and meta-analysis. Behavioural Brain Research (2016**) 296** (61-69).

Puig L., Lopez-Ferrer A.,Vilarrasa E.,Garcia I.,Fernandez-Del Olmo R. Model for assessing the efficiency of biologic drugs in the treatment of moderate to severe psoriasis for one year in clinical practice in Spain. Actas Dermo-Sifiliograficas (2016) **107**: (34-43).

Longden E., Read J. Assessing and reporting the adverse effects of antipsychotic medication: A systematic review of clinical studies, and prospective, retrospective, and cross-sectional research. Clinical Neuropharmacology (2016) **39**: (29-39).

Hoogedoorn L.,Gerritsen M.J.P.,Wolberink E.A.W.,Peppelman M.,van de Kerkhof P.C.M.,van Erp P.E.J. A four-phase strategy for the implementation of reflectance confocal microscopy in dermatology. Journal of the European Academy of Dermatology and Venereology (2016).

Maranda E.L.,Nguyen A.H.,Lim V.M.,Hafeez F.,Jimenez J.J.Laser and light therapies for the treatment of nail psoriasis.Journal of the European Academy of Dermatology and Venereology (2016).

Vaughn A.R.,Branum A.,Sivamani R.K. Effects of Turmeric (Curcuma longa) on Skin Health: A Systematic Review of the Clinical Evidence.Phytotherapy Research (2016).

DiMarco G,Hill D.,Feldman S.R.Review of patient registries in dermatologJournal of the American Academy of Dermatology (2016).

Carstens E.Many parallels between itch and pain researchEuropean Journal of Pain (United Kingdom) (2016) 20: (5-7).

Christakos S.,Dhawan P.,Verstuyf A,Verlinden L.,Carmeliet G. Vitamin D: Metabolism, molecular mechanism of action, and pleiotropic effects.Physiological Reviews (2016) **96**: (365-408).

Vsn M., Mothe R.K., Hyderboini R.K. et al. Systematic review and meta-analysis of briakinumab, a fully human interleukin 12/23 monoclonal antibody , for the treatment of moderate to severe chronic plaque psoriasis. Value in Health (2016) **19:** (A123).

Armstrong A., Betts K., Li J et al. Numbers needed to treat and costs per responder for novel treatments of moderate to severe psoriasis. Journal of the American Academy of Dermatology (2016) 74:5 SUPPL. 1 (AB261).

Ali F., Cueva A., Atwan A. Can systematic reviews help with choosing a suitable health-related quality of life measure in interventional studies of psoriasis?. International Journal of Clinical Pharmacy (2016) **38:**(565).

Shelton E., Laharie D., Scott F.I. Cancer Recurrence Following Immune-Suppressive Therapies in Patients With Immune-Mediated Diseases: A Systematic Review and Meta-analysis. Gastroenterology (2016).

2015

Sokolova A.,Lee A.,D Smith S.The Safety and Efficacy of Narrow Band Ultraviolet B Treatment in Dermatology: A Review.American Journal of Clinical Dermatology (2015) 16:(501-531).

Pereira R.,Lago P.,Faria R. et al.Safety of Anti-TNF Therapies in Immune-Mediated Inflammatory Diseases: Focus on Infections and Malignancy.Drug Development Research (2015) 76:(419-427).

Sorenson E.,Koo J. Evidence-based adverse effects of biologic agents in the treatment of moderate-to-severe psoriasis: Providing clarity to an opaque topic.Journal of Dermatological Treatment (2015**) 26:(**493-501).

Rogalski C.Calcipotriol/betamethasone for the treatment of psoriasis: Efficacy, safety, and patient acceptability.Psoriasis: Targets and Therapy (2015) 5 (97-107).

Stuart P.E.,Nair R.P.,Tsoi L.C. et al. Genome-wide Association Analysis of Psoriatic Arthritis and Cutaneous Psoriasis Reveals Differences in Their Genetic Architecture. American Journal of Human Genetics (2015) 97:(816-836)

Bowes J.,Loehr S.,Budu-Aggrey A et al.PTPN22 is associated with susceptibility to psoriatic arthritis but not psoriasis: Evidence for a further PSA-specific risk locus.Annals of the Rheumatic Diseases (2015) **74**:(1882-1885).

The international psoriasis council exome chip project: Exome arrays reveal known and novel coding variant associations.Journal of Investigative Dermatology (2015) 135 SUPPL. 2 (S57).

Song G.G.,Seo Y.H.,Kim J.-H et al.Association between TNF-Î± (-308 A/G,-238 A/G,-857 C/T polymorphisms and responsiveness to TNF-Î± blockers in spondyloarthropathy, psoriasis and Crohn's disease: A meta-analysis.Pharmacogenomics (2015) 16:(1427-1437).

Tsoi L.C.,Spain S.L.,Ellinghaus E. et al.Enhanced meta-analysis and replication studies identify five new psoriasis susceptibility loci.Nature Communications (2015) 6 Article Number: 7001.

Nititham J.,Taylor K.E.,Gupta R et al. Meta-analysis of the TNIP1 region in psoriasis identifies two independent association signals.Journal of Investigative Dermatology (2015) 135 SUPPL. 1 (S76).

Yin X.,Low H.,Seielstad M et al.Trans-ethnic genome-wide meta-analysis identifies multiple novel associations and reveals ethnic heterogeneity of psoriasis susceptibility.Journal of Investigative Dermatology (2015) 135 SUPPL. 1 (S74).

Swindell W.R.,Sarkar M.K.,Stuart P.E. et al.Psoriasis drug development and GWAS interpretation through in silico analysis of transcription factor binding sites.Clinical and Translational Medicine (2015) 4:1 Article Number: 13.

Song G.G.,Bae S.-C.,Kim J.-H et al.The angiotensin-converting enzyme insertion/deletion polymorphism and susceptibility to rheumatoid arthritis, vitiligo and psoriasis**:** A meta-analysis.JRAAS - Journal of the Renin-Angiotensin-Aldosterone System (2015) 16:(195-202).

Nititham J.,Taylor K.E.,Gupta R. et al.Meta-analysis of the TNFAIP3 region in psoriasis reveals a risk haplotype that is distinct from other autoimmune diseases. Genes and Immunity (2015) 16:2 (120-126). Date of Publication: 1 Mar 2015

Association between FOXP3 polymorphisms and susceptibility to autoimmune diseases: A meta-analysis**.**Lee M.-G.,Bae S.-C.Lee Y.H. et al. Autoimmunity (2015) 48:(445-452).

Meroni P.L.,Valentini G.,Ayala F et al New strategies to address the pharmacodynamics and pharmacokinetics of tumor necrosis factor (TNF) inhibitors A systematic analysisAutoimmunity Reviews (2015) **14:** (812-829).

Chen H.,Zhang T.,Gong B.,Cao X.Association between VEGF -634G/C polymorphism and susceptibility to autoimmune diseases: A meta-analysis.Gene (2015) 558:(181-186).

Jacobs A., Rosumeck S. Systematic review and meta-analysis of ustekinumab for moderate to severe psoriasis: Comment. Clinical and Experimental Dermatology (2015) **40**:(810-811).

Tada Y. What is the risk of inadvertent exposure to topical retinoids during first trimester pregnancy?. British Journal of Dermatology (2015) **173**: (1117-1118).

Puig L. On clinical thresholds, clinical equivalents and indirect comparisons of biological treatments for moderate-to-severe psoriasis. Journal of Clinical Pharmacy and Therapeutics (2015) **40:** (131-134).

Paternoster L., Standl M., Waage J. et al. Multi-ancestry genome-wide association study of 21,000 cases and 95,000 controls identifies new risk loci for atopic dermatitis. Nature Genetics (2015**) 47**: (1449-1456).

Sorensen J.A., Clemmensen K.K., Nixon R.L., Diepgen T.L., Agner T. Tobacco smoking and hand eczema - Is there an association?. Contact Dermatitis (2015) **73**: (326-335).

Honardoost M.A., Naghavian R., Ahmadinejad F et al. Integrative computational mRNA-miRNA interaction analyses of the autoimmune-deregulated miRNAs and well-known Th17 differentiation regulators: An attempt to discover new potential miRNAs involved in Th17 differentiation. Gene (2015) **572**: (153-162).

Lotti T., Hercogova J., Fabrizi G. Advances in the treatment options for vitiligo: Activated low-dose cytokines-based therapy. Expert Opinion on Pharmacotherapy (2015) **16**: (2485-2496).

Lee R.A., Eisen D.B. Treatment of hidradenitis suppurativa with biologic medications.Journal of the American Academy of Dermatology (2015) **73**: Supplement 1 (S82-S88).

Arora S.K., Dewan P., Gupta P. Microbiome: Paediatriciansâ perspective. Indian Journal of Medical Research (2015) **142:**NOVEMBER (515-524).

Fathi R., Armstrong A.W. The Role of Biologic Therapies in Dermatology. Medical Clinics of North America (2015) **99**: (1183-1194).

Simpson R.C. Cardiovascular disease risk factors in patients with hidradenitis suppurativa. British Journal of Dermatology (2015) **173**:(1118-1119).

A. Barbieri M., Capri S., Oskar B. Cost-utility analysis of apremilast for the treatment of moderate to severe plaque psoriasis in the Italian setting. Value in Health (2015) **18:** (A419-A420).

Mughal F., Cawston H., Kinahan D., Morris J., Tencer T., Zhang F. Cost-effectiveness of apremilast in moderate to severe psoriasis in Scotland. Value in Health (2015) **18**:7 (A420).

Carrascosa J.M., Vanaclocha F., Caloto T., Echave M., Oyaguez I.,Tencer T. Cost-utility analysis of apremilast for the treatment of moderate to severe psoriasis in Spain. Value in Health (2015) **18**: (A420).

VÃ¤Ã¤tÃ¤inen S., Soini E.J, Valgardsson V.S., MÃlkÃnen T. Cost-effectiveness of ustekinumab in the treatment of psoriasis in Finland. Value in Health (2015) **18**: (A670).

Gupta M., Mahajan V.K., Mehta K.S et al. Peroxisome proliferator-activated receptors (PPARs) and PPAR agonists: the â€˜futureâ in dermatology therapeutics?. Archives of Dermatological Research (2015) **307:** (767-780).

Mughal F., Cawston H., Cure S., Morris J., Tencer T. Zhang F. Cost-effectiveness of apremilast in psoriatic arthritis in Scotland. Value in Health (2015) **18:** (A644).

Capri S., Barbieri M., Oskar B. Cost-utility analysis of apremilast for the treatment of psoriatic arthritis in the Italian setting. Value in Health (2015) **18**:(A646).

Tzanetakos C, Vassilopoulos D., Kourlaba G., Christou P.,Maniadakis N. Cost-utility analysis of certolizumab pegol for the treatment of active psoriatic arthritis in Greece. Value in Health (2015) **18:** (A646-A647).

Nguyen C.M, Liao W. Genomic imprinting in psoriasis and atopic dermatitis: A review.Journal of Dermatological Science (2015) **80:** (89-93).

Capogrosso Sansone A., Mantarro S., Tuccori M et al. Safety Profile of Certolizumab Pegol in Patients with Immune-Mediated Inflammatory Diseases: A Systematic Review and Meta-Analysis.Drug Safety (2015) **38:** (869-888).

Bandino J.P., Hang A. ,Norton S.A. The Infectious and Noninfectious Dermatological Consequences of Flooding: A Field Manual for the Responding Provider. American Journal of Clinical Dermatology (2015) **16: (**399-424).

Ungprasert P., Srivali N., Upala S ET AL. Risk of venous thromboembolism in patients with ankylosing spondylitis: A systematic review and meta-analysis.American Journal of Respiratory and Critical Care Medicine (2015) 19.

Yamamoto T. Is tonsillectomy a therapeutic option for plaque-type psoriasis?. Journal of the American Academy of Dermatology (2015) **73**: (e153).

Iskandarani G., Ehsan Khan E, Lau-Walker M.O. Risk factors associated with atrial fibrillation in adults diagnosed under the age of 60-a systematic review. European Heart Journal: Acute Cardiovascular Care (2015) 4 SUPPL. 1 (78).

Yamamoto T. Optimal management of dactylitis in patients with psoriatic arthritis. Open Access Rheumatology: Research and Reviews (2015) **7** (55-62).

Murru A.,Popovic D, Pacchiarotti I., et al. Management of Adverse Effects of Mood Stabilizers. Current Psychiatry Reports (2015) **17**: Article Number: 66.

Edwards S.K., Bates C.M., Lewis F. et al. 2014 UK national guideline on the management of vulval conditions.International Journal of STD and AIDS (2015) **26:** (611-624).

Steven S., MÃnzel T., Daiber A. Exploiting the pleiotropic antioxidant effects of established drugs in cardiovascular disease. International Journal of Molecular Sciences (2015) **16:** (18185-18223).

Lee H.M., Liapakis A., Lim J.K. Diagnosis, management, and prevention of hepatitis B reactivation.Current Hepatitis Reports (2015) **14:** (184-194).

Wu S., Ding Y, Wu F, et al. Family history of autoimmune diseases is associated with an increased risk of autism in children: A systematic review and meta-analysis. Neuroscience and Biobehavioral Reviews (2015) **55** (322-332).

Friedman B., English J.C, Ferris L.K. Indoor Tanning, Skin Cancer and the Young Female Patient: A Review of the Literature. Journal of Pediatric and Adolescent Gynecology (2015) **28:** (275-283).

Blauvelt A., Armstrong A.W, Krueger G.G. Essential truths for the care and management of moderate-to-severe

psoriasis.Journal of Drugs in Dermatology (2015) **14**:(805-812).

Burden-Teh E., Lam M.L., Taibjee S.M, et al. How are we using systemic drugs to treat psoriasis in children? An insight into current clinical U.K. practice. British Journal of Dermatology (2015) **173:**(614-618).

Poddubnyy D., Van Tubergen A., LandewÃ R et al. Development of an ASAS-endorsed recommendation for the early referral of patients with a suspicion of axial spondyloarthritis. Annals of the Rheumatic Diseases (2015) **74**:(1483-1487)

Kim J.,Nadella P.,Kim D.J et al.Histological stratification of thick and thin plaque psoriasis explores molecular phenotypes with clinical implications. PLoS ONE (2015) **10:**Article Number: e0132454. D

Krasuska M., Millings A., Lavda A et al. Psychosocial well-being and quality of life in rosacea: A systematic literature review. British Journal of Dermatology (2015) **173** SUPPL. 1 (206).

Wong D., Cook R., Lee K.-A et al. Reliability of radiographic assessment of psoriatic arthritis mutilans. Journal of Rheumatology (2015) **42:** (1287).

Palmer J.B., Kanter S., Druyts E et al. Clinical factors that modify the effects of biologics on quality of life: A case study in psoriatic arthritis. Annals of the Rheumatic Diseases (2015) 74 SUPPL. 2 (1170).

Higgins S., Wesley N.O. Topical Retinoids and Cosmeceuticals: Where Is the Scientific Evidence to Recommend Products to Patients?. Current Dermatology Reports (2015) **4:** (56-62).

Palmer J.B., Kanter S., Druyts E et al. Clinical factors that modify treatment effects of biologics in psoriatic arthritis: A systematic review and meta-regression.Annals of the Rheumatic Diseases (2015) **74** (1169-1170).

Gieler U., Gieler T., Schut C. et al. Quality of Life and Comorbidities in Urticaria: What Is Known?. Current Dermatology Reports (2015) **4**: (77-82)

Yao R., Du Y.Y., Zhang Y.Z et al. Association between G-217A polymorphism in the AGT gene and essential hypertension: A meta-analysis. Genetics and Molecular Research (2015) **14**:(5527-5534).

Vena G.A., Cassano N, Bellia G. et al .Psoriasis in pregnancy: Challenges and solutions. Psoriasis: Targets and Therapy (2015) 5 (83-95).

Kingsley G., H.Scott D.L. Assessing the effectiveness of synthetic and biologic disease-modifying antirheumatic drugs in psoriatic arthritis - A systematic review. Psoriasis: Targets and Therapy (2015) 5 (71-81).

Tencer T., Clancy Z., Damera V et al. Economic evaluation of apremilast in the treatment of moderate to severe psoriasis in the United States.Journal of the American Academy of Dermatology (2015) 72: SUPPL. 1 (AB97).

Lee A, Gregory V, Gu Q. Cost-effectiveness of secukinumab compared to current treatments for the treatment of moderate to severe plaque psoriasis in Canada.Value in Health (2015) **18: (**A182).

Feldman S.R.,Tencer T., Clancy Z et al. Cost per responder of apremilast versus etanercept, adalimumab, and ustekinumab in patients with moderate to severe psoriasis. Journal of the American Academy of Dermatology (2015) **72**: SUPPL. 1 (AB229).

Gupta P., Mandema J.W., Ahadieh S. et al. A model-based metaanalysis for dose-response comparison of psoriasis treatments. Journal of the American Academy of Dermatology (2015) **72:** SUPPL. 1 (AB221).

Alberdi T.,Baldwin B. Development of cutaneous T-cell lymphoma during adalimumab monotherapy: A case

report. Journal of the American Academy of Dermatology (2015**) 72**: SUPPL. 1 (AB157).

Nair R.P.,Tsoi L.C.,Ghosh M et al. Genome wide association study of psoriasis in India.Journal of Investigative Dermatology (2015) **135** SUPPL. 1 (S75).

Foster S., Zhu B., Al Sawah S. Cost per additional responder associated with biologic use in psoriasis. Value in Health (2015) **18**: (A297).

Goetghebeur M.M.,Wagner M.,Nikodem M et al. Combining MCDA with advanced statistics to tackle challenges of data and judgment uncertainty: Case study of safety assessments. Value in Health (2015) 18: (A12-A13)

Bowes J.,Loehr S.,Budu-Aggrey APTPN22 is associated with susceptibility to psoriatic arthritis but not psoriasis: Evidence for a further PsA-specific risk locus. Annals of the Rheumatic Diseases (2015).

Duarte G.V.,Porto-Silva L.De Paim ,De Oliveira M.F.Epidemiology and treatment of psoriasis: A Brazilian perspective.Psoriasis: Targets and Therapy (2015) **5** (55-64).

Tsoi L.C., Elder J.T., Abecasis G.R. Graphical algorithm for integration of genetic and biological data: proof of principle using psoriasis as a model. Bioinformatics (Oxford, England) (2015) 31: (1243-1249).

Puig L. PASI90 response: The new standard in therapeutic efficacy for psoriasis. Journal of the European Academy of Dermatology and Venereology (2015) 29:4 (645-648).

Dulai P.S, Mosli M., Khanna R et al. Vedolizumab for the treatment of moderately to severely active ulcerative colitis. Pharmacotherapy (2015) **35:** (412-423

Chandra A., Ray A., Senapati S., Chatterjee R. Genetic and epigenetic basis of psoriasis pathogenesis.Molecular Immunology (2015) **64**: (313-323).

Marrie R.A., Reider N., Cohen J et al. A systematic review of the incidence and prevalence of autoimmune disease in multiple sclerosis. Multiple Sclerosis Journal (2015) 21: (282-293).

Marrie R.A. Cohen J., Stuve O et al. A systematic review of the incidence and prevalence of comorbidity in multiple sclerosis: Overview. Multiple Sclerosis Journal (2015) **21:** (263-281).

Feuerstein J.D., Cullen G., Cheifetz A.S. Immune-mediated reactions to anti-tumor necrosis factors in inflammatory bowel disease. Inflammatory Bowel Diseases (2015) 21:5 (1176-1186).

Radack K.P., Farhangian M.E., Anderson K.L., Feldman S.R.A. Review of the Use of Tanning Beds as a Dermatological Treatment.Dermatology and Therapy (2015) **5:** (37-51).

Sawyer L.M., Wonderling D., Jackson K. et al. Biological Therapies for the Treatment of Severe Psoriasis in Patients with Previous Exposure to Biological Therapy: A Cost-Effectiveness Analysis.PharmacoEconomics (2015**) 33:(**163-177).

Atzeni F., Masala I.F., Salaffi F. et al. Pain in systemic inflammatory rheumatic diseases.Best Practice and Research: Clinical Rheumatology (2015) **29**:(42-52) Article Number: 1131.

Alunno A., Carubbi F., Cafaro G et al. Targeting the IL-23/IL-17 axis for the treatment of psoriasis and psoriatic arthritis. Expert Opinion on Biological Therapy (2015) **15**: (1727-1737).

Abbott R., Whear R., Nikolaou V et al. Tumour necrosis factor-Î± inhibitor therapy in chronic physical illness: A systematic review and meta-analysis of the effect on depression and anxiety. Journal of Psychosomatic

Research (2015) **79:** (175-184).

Wu D., Guo Y.-Y., Xu N.-N et al. Efficacy of anti-tumor necrosis factor therapy for extra-articular manifestations in patients with ankylosing spondylitis: A meta-analysis.BMC Musculoskeletal Disorders (2015) **16:** Article Number: 19.

Siu S.,Haraoui B.,Bissonnette R et al Meta-analysis of tumor necrosis factor inhibitors and glucocorticoids on bone density in rheumatoid arthritis and ankylosing spondylitis trials.Arthritis Care and Research (2015) **67:** (754-764).

Ahn C.S., Dothard E.H., Garner M.L et al. To test or not to test? An updated evidence-based assessment of the value of screening and monitoring tests when using systemic biologic agents to treat psoriasis and psoriatic arthritis Presented in poster form at the 73rd Annual Meeting of the American Academy of Dermatology, San Francisco, CA, March 20-24, 2015.Journal of the American Academy of Dermatology (2015) **73:**(420-428.e1).

Conway R., Low C., Coughlan R.J et al. Risk of liver injury among methotrexate users: A meta-analysis of randomised controlled trials. Seminars in Arthritis and Rheumatism (2015) **45:** (156-162).

Correia B, Torres T. Obesity: A key component of psoriasis. Acta Biomedica (2015**) 86:(**121-129).

Broersen L.H.A., Pereira A.M., JÃ¸rgensen J.O.L., Dekkers O.M. Adrenal insufficiency in corticosteroids use: Systematic review and meta-analysis. Journal of Clinical Endocrinology and Metabolism (2015) **100:** (2171-2180).

Van Laarhoven A.I.M.,Van Der Sman-Mauriks I.M.,Donders A.R.T et al. Placebo effects on itch: A meta-analysis of clinical trials of patients with dermatological conditions. Journal of Investigative Dermatology (2015) 135:5 (1234-1243).

Dawwas M.F., Aithal G.P. The quest for an evidence-based approach to surveillance for methotrexate-related hepatotoxicity: Promise and perils. British Journal of Dermatology (2015) **172:**(1684-1685).

Juncadella A.C., Alame A.M., Sands L.R et al. Perianal crohn disease: A review. Postgraduate Medicine (2015) **127:(**266-272).

Yiu Z.Z.N.,Warren R.B.,Mrowietz U.,Griffiths C.E.M.Safety of conventional systemic therapies for psoriasis on reproductive potential and outcomes. Journal of Dermatological Treatment (2015) **26:** (329-334).

Drugs for psoriatic arthritis. Medical Letter on Drugs and Therapeutics (2015) **57:** (88-92).

Hoffman M.B., Farhangian M., Feldman S.R. Psoriasis during pregnancy: Characteristics and important management recommendations.Expert Review of Clinical Immunology (2015) 11: (709-720).

Stolwijk C., Van Tubergen A., Castillo-Ortiz J.D., Boonen A. Prevalence of extra-articular manifestations in patients with ankylosing spondylitis: A systematic review and meta-analysis.Annals of the Rheumatic Diseases (2015) **74:** (65-73).

Azimi E., Lerner E.A., Elmariah S.B. Altered manifestations of skin disease at sites affected by neurological deficit. British Journal of Dermatology (2015) **172**: (988-993).

Manriquez J., Andino-Navarrete R., Cataldo-Cerda K., Harz-Fresno I. Bibliometric characteristics of systematic reviews in dermatology: A cross-sectional study through Web of Science and Scopus. Dermatologica Sinica (2015) **33:** (154-156).

Wu J.J., Choi Y.M. Time to consider psoriasis an autoimmune disorder?. Journal of Drugs in Dermatology

(2015) **14:**(112).

Eder L.,Gladman D.D.Atherosclerosis in psoriatic disease: latest evidence and clinical implications.Therapeutic Advances in Musculoskeletal Disease (2015) **7:** (187-195).

Spinicci M., Mencarini J.,Goletti D. et al. Discordance between the QuantiFERON-TB Gold In-Tube and Tuberculin Skin Test: Need for a further step?. European Respiratory Journal (2015) **46:** (1506-1509).

Tajaddini M.H., Keikha M., Razzazzadeh A, Kelishadi R. A systematic review on the association of serum selenium and metabolic syndrome.Journal of Research in Medical Sciences (2015**) 20:** (782-789).

CÄƒruntu C., Negrei C., GhiÅ£Äƒ M.A et al. Capsaicin, a hot topic in skin pharmacology and physiology. Farmacia (2015) **63:** (487-491).

Boehncke W.-H., Anliker M.D., Conrad C et al. The dermatologists' role in managing psoriatic arthritis: Results of a swiss delphi exercise intended to improve collaboration with rheumatologists. Dermatology (2015) **230**:(75-81).

KÃtter I. The challenging treatment of Behcets disease. Expert Opinion on Orphan Drugs (2015) 3: (1101-1110).

Sritheran D., Leung Y.Y. Making the next steps in psoriatic arthritis management: current status and future directions. Therapeutic Advances in Musculoskeletal Disease (2015) **7:** (173-186).

Mahajan B.B., Kaur S. Interferons. Indian Journal of Dermatology, Venereology and Leprology (2015) 81:(51-55).

Torales-Cardeña A., Martínez-Torres I., Rodríguez-Martínez S et al. Cross talk between proliferative, angiogenic, and cellular mechanisms orchestred by HIF-1 in Psoriasis. Mediators of Inflammation (2015) 2015 Article Number: 607363.

Anglada-Martinez H., Riu-Viladoms G., Martin-Conde M et al. Does mHealth increase adherence to medication? Results of a systematic review.International Journal of Clinical Practice (2015) **69:** (9-32).

Smith M.D. Review: Methotrexate does not increase risk for lung disease in psoriasis, psoriatic arthritis, or IBD. Annals of Internal Medicine (2015) **163:(**JC7).

Armstrong A.W., Florek A.G.Response to: Is tonsillectomy a therapeutic option for plaque-type psoriasis?. Journal of the American Academy of Dermatology (2015) **73:** (e155).

Gupta A.K., Daigle D., Foley K.A. The prevalence of culture-confirmed toenail onychomycosis in at-risk patient populations. Journal of the European Academy of Dermatology and Venereology (2015) **29:**(1039-1044).

Onuora S. Genetics: Meta-analysis reveals novel overlap in genetic aetiologies of paediatric autoimmune disorders. Nature Reviews Rheumatology (2015) **11:**(561).

Carrascosa J.M., Belinchon I., De-La-Cueva P et al. Expert recommendations on treating psoriasis in special circumstances. Actas Dermo-Sifiliograficas (2015) **106:** (292-309).

Goff K., Boyers L.N., Karimkhani C et al. Disease Burden Measures: a Review.Current Dermatology Reports (2015) **4:**(30-35).

Ogdie A., Weiss P. The Epidemiology of Psoriatic Arthritis Rheumatic Disease Clinics of North America (2015) **41:**545-568).

Christensen R., Singh J.A, Wells G.A. et al. Do evidence-based recommendations need to reveal the evidence?

Minimal criteria supporting an evidence claim.Journal of Rheumatology (2015) **42:** (1737-1739).

Weisman M.H. Psoriatic Arthritis.Rheumatic Disease Clinics of North America (2015) 41:4 (xi).

Baurecht H., Hotze M., Brand S et al. Genome-wide comparative analysis of atopic dermatitis and psoriasis gives insight into opposing genetic mechanisms. American Journal of Human Genetics (2015) **96:** (104-120).

Murrell D.F., Marinovic B., Caux F et al. Definitions and outcome measures for mucous membrane pemphigoid: Recommendations of an international panel of expertsJournal of the American Academy of Dermatology (2015) **72:** (168-174).

Haddad A., Johnson S.R, Somaily M et al. Psoriatic arthritis mutilans: Clinical and radiographic criteria. A systematic review. Journal of Rheumatology (2015) **42:** (1432-1438).

Jadon D.R., Nightingale A.L., McHugh N.J et al. Serum soluble bone turnover biomarkers in psoriatic arthritis and psoriatic spondyloarthropathy Journal of Rheumatology (2015) **42:** (21-30).

Kroon F.P., van der Burg L.R., Ramiro S et al. Non-steroidal anti-inflammatory drugs (NSAIDs) for axial spondyloarthritis (ankylosing spondylitis and non-radiographic axial spondyloarthritis). The Cochrane database of systematic reviews (2015) 7 (CD010952).

Zhao C.Y., Murrell D.F. Outcome measures for autoimmune blistering disease.Journal of Dermatology (2015) **42: (**31-36).

Balak D.M.W. Fumaric acid esters in the management of psoriasis. Psoriasis: Targets and Therapy (2015) 5 (9-23).

Ungprasert P., Cheungpasitporn W., Thongprayoon C et al. Association between chronic obstructive pulmonary disease and psoriasis: **A** systematic review and meta-analysis. American Journal of Respiratory and Critical Care Medicine (2015) 191 MeetingAbstracts.

Ali F.M.,Cueva A.,Vyas J.,Piguet V et al. Quality of life measurement in therapeutic trials of psoriasis: A systematic review**.** Journal of Investigative Dermatology 47.

Manalo I.F.,Gilbert K.E.,Wu J.J. Subcutaneous methotrexate for symptomatic control of severe recalcitrant psoriasis: Safety, efficacy, and patient acceptability. Psoriasis: Targets and Therapy (2015) 5 (65-70).

Florek A.,Rachakonda T,Dhillon J et al.A systematic review of tonsillectomy as a treatment for guttate or plaque psoriasis.Journal of the American Academy of Dermatology (2015) **72:**SUPPL. 1 (AB223).

S.H.,Gao Q.,Wu J.C et al.Effect of immunosuppressive therapy on interferon gamma release assay (IGRA) in immune-mediated inflammatory diseases: A meta-analysis of 17 cohort studie.Gastroenterology (2015) **148:** SUPPL. 1 (S433)

Brezinski E.A.,Dhillon J.S.,Armstrong A.W.Economic burden of psoriasis in the United States a systematic review.JAMA Dermatology (2015) **151:(**651-658).

Van Bezooijen J.S.,Prens E.P.,Pradeepti M.S et al.Combining biologics with methotrexate in psoriasis: A systematic review. British Journal of Dermatology (2015) **172:**(1676-1680).

Gilbert K.E.,Manalo I.F.,Wu J.J.Efficacy and safety of etanercept and adalimumab with and without a loading dose for psoriasis: A systematic review. Journal of the American Academy of Dermatology (2015) **73:(**329-331).

Wang L.,Yang H.,Li N et al.Acupuncture for psoriasis: Protocol for a systematic review. BMJ Open (2015) 5:6 Article Number: e007526.

Kalliker Frers R.A., Bisoendial R.J., Montoya S.F et al. Psoriasis and cardiovascular risk: Immune-mediated crosstalk between metabolic, vascular and autoimmune inflammation. IJC Metabolic and Endocrine (2015) 6 (43-54).

Vanderpuye-Orgle J."",Zhao Y.,Lu J et al.Evaluating the economic burden of psoriasis in the United States. Journal of the American Academy of Dermatology (2015) 72:6 (961-967.e5).

2014

Jadali Z.,Eslami M.B.   T cell immune responses in psoriasis.Iranian Journal of Allergy, Asthma and Immunology (2014) **13:(**220-230).

Zschocke I.,Mrowietz U.,Lotzin A et al. Assessing adherence factors in patients under topical treatment: Development of the Topical Therapy Adherence Questionnaire (TTAQ). Archives of Dermatological Research (2014) **306:** (287-297).

Richard M.-A.,Paul C.Risk factors, diagnosis and management of psoriatic arthritis: Systematic literature reviews and expert opinion of a panel of dermatologists.Journal of the European Academy of Dermatology and Venereology (2014) **28**:SUPPL. 5 (1-2).

Berki D., Burden D., Choon S.-E et al. The p.Asp176His CARD14 variant is associated with generalized pustular psoriasis presenting with concurrent psoriasis vulgaris. British Journal of Dermatology (2014) **171:** (e147).

Suarez-Farinas M.,Belasco J.,Sullivan T.,Arbeit R.,Krueger J.G et al.Treatment of psoriasis patients with IMO-3100 shows improvement in gene expression patterns of meta-analysis derived-3 transcriptome and IL-17 pathway.Arthritis and Rheumatism (2013) 65 SUPPL. 10 (S495).

Zhuang L.,Ma W.,Cai D et al.Associations between tumor necrosis factor-Î± polymorphisms and risk of psoriasis: A meta-analysis.PLoS ONE (2013) 8:12 Article Number: e68827.

Gu W.-J.,Weng C.-L.,Zhao Y.-T et al.Psoriasis and risk of cardiovascular disease: A meta-analysis of cohort studies.International Journal of Cardiology (2013) 168:5 (4992-4996).

Mezentsev A.V.,Bruskin S.A.,Soboleva A.G et al.Pharmacological control of receptor of ddvanced glycation end-products and its biological effects in psoriasis.International Journal of Biomedical Science (2013) **9:(**112-122).

Zhu J.Q.,Li J.,Qu H.D et al. Tumor necrosis factor-alpha gene promoter region single nucleotide polymorphism change the susceptibility to psoriasis vulgaris and psoriatic arthritis**:** A meta-analysis. International Journal of Rheumatic Diseases (2013) 16 SUPPL. 1 (39).

Femia A.N.,Vleugels R.A.,Callen J.P.Cutaneous dermatomyositis: An updated review of treatment options and internal associations. American Journal of Clinical Dermatology (2013) **14:** (291-313).

Mandema J.,Peterson M.,Ahadieh S et al.Evaluation of the impact of body weight on the efficacy of biologic therapies for the treatment of psoriasis: A dose-response meta-analysis. Journal of the European Academy of Dermatology and Venereology (2013) 27 SUPPL. 4 (11-12).

Nast A.,Schmitt J.Physician global assessment (PGA) and psoriasis area and severity index (PASI): Why do

both? A systematic analysis of randomized controlled trials of biologic agents for moderate to severe plaque psoriasis.Journal of the American Academy of Dermatology (2013) 68:(1040-1041).

J.L.,Zhang S.Q.,Zeng H.M. ApaI, BsmI, FokI and TaqI polymorphisms in the vitamin D receptor (VDR) gene and the risk of psoriasis: A meta-analysis. Journal of the European Academy of Dermatology and Venereology (2013) 27:6 (739-746).

Zhu J.,Qu H.,Chen X et al. Single Nucleotide Polymorphisms in the Tumor Necrosis Factor-Alpha Gene Promoter Region Alter the Risk of Psoriasis Vulgaris and Psoriatic Arthritis: A Meta-Analysis.PLoS ONE (2013) 8:5 Article Number: e64376.

loci.Nair R.P.,Tsoi L.C.,Stuart P.E. et al.Meta-analysis of psoriasis and psoriatic arthritis identifies three new susceptibility Journal of Investigative Dermatology (2013) 133 SUPPL. 1 (S136).

A network meta-analysis of randomized, controlled trials of ustekinumab and adalimumab for moderate-to-severe psoriasis.Odom D.,Brogan A.,Talbird S.E et al.Value in Health (2013) 16:3 (A112).

Gustafson C.J.,Watkins C.,Hix E et al Combination therapy in psoriasis: An evidence-based review.American Journal of Clinical Dermatology (2013) **14:(**9-25).

Pietrzak A.,BartosiÅ„ska J.,Chodorowska G et al.Cardiovascular aspects of psoriasis: An updated review.International Journal of Dermatology (2013) **52:**(153-162).

Capon F., Hussain S.,Berki D et al. IL36RN mutations define a severe, early-onset subtype of generalized pustular psoriasis. British Journal of Dermatology (2014) **171:(**e113-e114).

Gulliver W., Gulliver S., Randell S. Serum ferritin levels as an indicator of anaemia in patients with moderate-to-severe psoriasis compared with the general public.British Journal of Dermatology (2014) **171:** (e161).

Roper D., Al-nuaimi Y., Oza H et al. A systems biological model of chronic plaque psoriasis captures dynamics of known treatments. British Journal of Dermatology (2014) **171:**(e146).

Nair R., Stuart P., Tsoi L et al. Genome-wide association analysis of psoriatic arthritis. British Journal of Dermatology (2014) **171:**(e111).

Simpson M.Identification of known and novel coding variant associations utilising exome arrays: The international psoriasis council exome chip project.British Journal of Dermatology (2014) **171:** (e112-e113)

Strohal R.,Kirby B,Puig L.Psoriasis beyond the skin: An expert group consensus on the management of psoriatic arthritis and common co-morbidities in patients with moderate-to-severe psoriasis .Journal of the European Academy of Dermatology and Venereology (2014) **28:**(1661-1669).

Wang Y.-D.,Chen H.,Liu H.-Q Correlation between ovarian neoplasm and serum levels of osteopontin: a meta-analysis.Tumor Biology (2014) **35:(**11799-11808).

Burudpakdee C.,Khan Z.M.,Gala S.Impact of patient programs on adherence in inflammation and immunology: A global systematic review and meta-analysis of published evidence.Value in Health (2014) **17:**(A534).

Tsoi L,Spain S.,Ellinghaus E et al.Enhanced meta-analysis and replication studies identify five psoriasis susceptibility loci.British Journal of Dermatology (2014) 171:6 (e112).

Chi C.-C.,Wang S.-H.Cost-efficacy of biologic therapies for moderate to severe psoriasis from the perspective of the Taiwanese health care system. Pharmacoepidemiology and Drug Safety (2014) 23 SUPPL. 1 (5).

Siu S.,Haraoui B.,Roubille C. et al.Effect of disease modifying drugs on bone mineral density in patients with rheumatoid arthritis, psoriatic arthritis, psoriasis, and ankylosing spondylitis: A meta-analysis.Annals of the Rheumatic Diseases (2014) 73 SUPPL. 2.

Chen M.-L.,Huang J.,Xie Z.-F. et al.Associations between the major histocompatibility complex class i chain-related gene A transmembrane (MICA-TM) polymorphism and susceptibility to psoriasis and psoriatic arthritis: A meta-analysis.Rheumatology International (2014) 34: (297).

Chi C.-C.,Wang S.-H. Efficacy and cost-efficacy of biologic therapies for moderate to severe psoriasis: A meta-analysis and cost-efficacy analysis using the intention-to-treat principle.BioMed Research International (2014) 2014 Article Number: 862851.

Xia J.,Zhang W.A meta-Analysis revealed insights into the sources, conservation and impact of microRNA 5â€²-isoforms in four model species.Nucleic Acids Research (2014) **42:**(1427-1441).

Dogra S.,Yadav S.Acitretin in psoriasis: An evolving scenarioInternational Journal of Dermatology (2014) **53:** (525-538).

Jensen J.D.,Delcambre M.R.,Nguyen G et al.Biologic Therapy with or Without Topical Treatment in Psoriasis: What Does the Current Evidence Say?American Journal of Clinical Dermatology (2014) **15:** (379-385).

Liu Y.,Gong J.-P",Li W.-F.Therapeutic effect and safety of ustekinumab for plaque psoriasis: A meta-analysis.Chinese Medical Sciences Journal (2014) **29:**(131-138).

G.G., KimJ.-H.,Lee Y.H. Associations between the major histocompatibility complex class i chain-related gene A transmembrane (MICA-TM) polymorphism and susceptibility to psoriasis and psoriatic arthritis: A meta-analysis. Rheumatology International (2014) **34:**(117-123).

Cardona-Arias J.A.,Franco-Aguirre J.Q.Effect of the psoriasis on the health-related quality of life: Meta-analyses 2003-2013**.**Revista Argentina de Dermatologia (2014) 95:2.

Bhatia B.K.,Millsop J.W.,et al Debbaneh M.Diet and psoriasis, part II: Celiac disease and role of a gluten-free diet. Journal of the American Academy of Dermatology (2014) **71:(**350-358).

C-reactive protein in psoriasis: A review of the literature.Beygi S,Lajevardi V.,Abedini R.Journal of the European Academy of Dermatology and Venereology (2014) 28:(700-711).

Armstrong A.W.,Tuong W.,Love T.J et al.Treatments for nail psoriasis: A systematic review by the GRAPPA nail psoriasis work group.Journal of Rheumatology (2014) 41: (2306-2314).

Tangwongsiri D.,Leartsakulpanitch J.Cost utility analysis of ustekinumab for the treatment of moderate to severe chronic plaque psoriasis in Thailand.Value in Health (2014) **17:** (A782-A783).

Ogdie A.,Schwartzman S.,Eder L et al .Comprehensive treatment of psoriatic arthritis: Managing comorbidities and extraarticular manifestations. Journal of Publication: 1 Nov 2014

Bouwmeester W,Van Beurden-Tan C,Bennison C.,Heeg B. The proportional odds model is more efficient than the multinomial logistic model for network meta-analyses of ordered outcomes.Value in Health (2014) **17:** (A566).

Conway R.,Low C.,Coughlan R.Methotrexate and lung disease-a meta-analysis of randomized controlled trials. Irish Journal of Medical Science (2014) **183**: SUPPL. 1 (S516-S517).

Orbai A.-M.,Weitz J.,Siegel E.L et al.Systematic review of treatment effectiveness and outcome measures for enthesitis in psoriatic arthritis. Journal of Rheumatology (2014) **41:** (2290-2294).

Rose S.,Toloza S.,Bautista-Molano W et al.Comprehensive treatment of dactylitis in psoriatic arthritis. Journal of Rheumatology (2014) **41:** (2295-2300)

Nanau R.M.,Cohen L.B.,Neuman M.G .Risk of infections of biological therapies with accent on inflammatory bowel disease. Journal of Pharmacy and Pharmaceutical Sciences (2014) **17:** (485-528).

Capogrosso-Sansone A.,Mantarro S.,Blandizzi C et al.Update of certolizumab pegol safety profile: A systematic review and meta-analysis.Drug Safety (2014) **37:**(844-845).

Goel N.,Charnce K.Arthritis and Rheumatology (2014) 66 SUPPL. 10 (S662). Date of Publication: October 2014

Almaghlouth I.,Thava A.,Haroon N.,Inman R.D. Differing patterns of axial spondyloarthritis in females and males.Arthritis and Rheumatology (2014) 66 SUPPL. 10 (S263-S264).

Tencer T.,Clancy Z.,Cawston H et al .Economic evaluation of sequencing strategies in the treatment of psoriatic arthritis in the united states. Arthritis and Rheumatology (2014) 66 SUPPL. 10 (S705).

LÃ¸nnberg A.S.,Zachariae C.,Skov L.Targeting of interleukin-17 in the treatment of psoriasis.Clinical, Cosmetic and Investigational Dermatology (2014) 7 (251-259).

Dregan A.,Charlton J.,Chowienczyk P et al.Chronic inflammatory disorders and risk of type 2 diabetes mellitus, coronary heart disease, and stroke : A population-based cohort study. Circulation (2014) **130:** (837-844).

Baurecht H,Hotze M.,Elder J.T et al .Genome-wide comparative analysis of atopic dermatitis and psoriasis gives insight into shared and opposing genetic risk mechanisms. Journal of Investigative Dermatology (2014) 134 SUPPL. 2 (S1).

Mounach A.,El Maghraoui A. Efficacy and safety of adalimumab in ankylosing spondylitis. Open Access Rheumatology: Research and Reviews (2014) 6 (83-90).

Nanau R.M.,Neuman M.G.Safety of anti-tumor necrosis factor therapies in arthritis patient.Journal of Pharmacy and Pharmaceutical Sciences (2014) **17**: (324-361).

Farrell E.,Whistance R.,Lloyd A et al.Shared decision-making in plaque psoriasis: Development of an Option Grid to assist in decisions to commence oral therapy. British Journal of Dermatology (2014) 171 SUPPL. 1 (7).

Siu S.,Haraoui B.,Keeling S et al.The effects of tumor necrosis factor inhibitors and corticosteroids on bone mineral density in patients with rheumatoid arthritis and ankylosing spondylitis: A meta-analysis of randomized controlled trials.Journal of Rheumatology (2014) 41:(1491).

De Vecchis R.,Palmisani L.,Pucciarelli A et al .Protective effects of methotrexate against ischemic cardiovascular disorders in patients treated for rheumatoid arthritis or psoriasis: Novel therapeutic insights coming from a meta-analysis of the literature data.Giornale Italiano di Cardiologia (2014) 15:SUPPL. 2 (e54).

Queiro R.,RodrÃguez S.,Acasuso B et al.An onset of psoriasis after 40 years and a low education level may predict the development of diabetes mellitus in psoriatic arthritis. Annals of the Rheumatic Diseases (2014) 73 SUPPL. 2.

Panchal S.,Flint J,Van De Venne M et al.A systematic analysis of the safety of prescribing of anti-rheumatic, immunosuppressive and biologic drugs in men trying to conceive. Annals of the Rheumatic Diseases (2014) 73 SUPPL. 2.

Jani M.,Massey J.,Wedderburn L et al.Genetic risk factors in idiopathic inflammatory myopathies are shared with other autoimmune disorders in european populations.Annals of the Rheumatic Diseases (2014) 73 SUPPL. 2.

Australasian College of Dermatologists 47th Annual ScientificMeetin.Australasian Journal of Dermatology (2014) 55 SUPPL. 1.

Roques C.F. Balneotherapy actual medical benefit. Data of evidence for the last twenty years.Annals of Physical and Rehabilitation Medicine (2014) 57 SUPPL. 1 (e159).

Azimi E.,Lerner E.A.,Elmariah S.Altered manifestations of skin disease in patients with nerve damage.Journal of Investigative Dermatology (2014) 134 SUPPL. 1 (S19).

Bigby M.Understanding and evaluating systematic reviews and meta-analyses Indian Journal of Dermatology (2014) 59:(134-139).

Hotze M.,Baurecht H.,Rodriguez E et al .Genome-wide comparative analysis of atopic eczema and psoriasis gives insight into disease mechanisms.Experimental Dermatology (2014) 23:3 (e21).

Bang C.N.,Okin P.M. Statin treatment, new-onset diabetes, and other adverse effects: A systematic review.Current Cardiology Reports (2014) 16:3.

Lallas A.,Giacomel J.,Argenziano G.Dermoscopy in general dermatology: Practical tips for the clinician.British Journal of Dermatology (2014) **170:** (514-526).

Krishnareddy S.,Swaminath A. When combination therapy isn't working: Emerging therapies for the management of inflammatory bowel disease. World Journal of Gastroenterology (2014) **20:** (1139-1146).

Frez M.L.F. Asawanonda P., Gunasekara C et al. Recommendations for a patient-centered approach to the assessment and treatment of scalp psoriasis: A consensus statement from the Asia Scalp Psoriasis Study Group. Journal of Dermatological Treatment (2014) 25:1 (38-45).

de Eusebio E., Armario-Hita J.C., de Miquel V.A. Treatment of Psoriasis: Focus on Clinic-based Management with Infliximab American Journal of Clinical Dermatology (2014) 15:1 (5-16).

Lynch M.,Kirby B.,Warren R.B. Treating moderate to severe psoriasis - Best use of biologics. Expert Review of Clinical Immunology (2014) 10:2 (269-279).

Churton S.,Brown L.Shin T.M.,Korman N.J.Does treatment of psoriasis reduce the risk of cardiovascular disease?.Drugs (2014) 74:2 (169-182).

Semble A.L.,Davis S.A.,Feldman S.R. Safety and tolerability of tumor necrosis factor-Î± inhibitors in psoriasis: A narrative review.American Journal of Clinical Dermatology (2014) **15: (**37-43).

Dommasch E.D.,Troxel A.B.,Gelfand J.M.Counterpoint: A tale of two meta-analyses revisited.Journal of the American Academy of Dermatology (2014) 70: (381-383).

Longworth L.,Yang Y.,Young et al.Use of generic and condition-specific measures of health-related quality of life in NICE decision-making: A systematic review, statistical modelling and survey.Health Technology Assessment (2014) 18:(1-224).

Tzellos T.,Kyrgidis A.,Trigoni A et al. Point: Major adverse cardiovascular events and anti-IL 12/23 agents.Journal of the American Academy of Dermatology (2014) 70:2 (380-381).

Hashkes P.J., Becker M.L., Cabral D.A et al. Methotrexate: New uses for an old drug. Journal of Pediatrics (2014) **164:**(231-236).

Cawson M.R.,Mitchell S.A.,Knight C et al. Systematic review, network meta-analysis and economic evaluation of biological therapy for the management of active psoriatic arthritis.BMC Musculoskeletal Disorders (2014) 15: Article Number: 26.

Lubrano E.,Spadaro A Pharmacoeconomic burden in the treatment of psoriatic arthritis: From systematic reviews to real clinical practice studies. BMC Musculoskeletal Disorders (2014) 15:Article Number: 25.

Alexander W.American academy of dermatology and American college of cardiologyP and T (2014) 39:5 (370-374).

Coates L.C.,Kavanaugh A.,Ritchlin C.T.Systematic review of treatments for psoriatic arthritis: 2014 update for the GRAPPA. Journal of Rheumatology (2014) 41:(2273-2276).

Sozzani S.,Abbracchio M.P.,Annese V et al. Chronic inflammatory diseases:Do immunological patterns drive the choice of biotechnology drugs? A critical review.Autoimmunity (2014) 47:5 (287-306

Armuzzi A.,Lionetti P.,Blandizzi C.Anti-TNF Agents as Therapeutic Choice in Immune-Mediated Inflammatory Diseases: Focus on Adalimumab.International Journal of Immunopathology and Pharmacology (2014) 27 Supplement 1 (11-32).

Ahlehoff O.,Gislason G,HanseCardiovascular aspects of psoriasis: An updated review.P.R. International Journal of Dermatology (2014) **53:** (e337-e337).

Kragballe K.,Van De Kerkhof P.C.,Gordon K.B.Unmet needs in the treatment of psoriasis.European Journal of Dermatology (2014) **24:** (523-532).

AniÄ‡ B.,Padjen I.,Mayer M.Clinical features of the SAPHO syndrome and their role in choosing the therapeutic approach: Report of four patients and review of the literature.Acta Dermatovenerologica Croatica (2014**) 22:** (180-188).

Lis K.,KuzawiÅ„ska O.,BaÅ‚kowiec-Iskra E.Tumor necrosis factor inhibitors - State of knowledge.Archives of Medical Science (2014) **10:** (1175-1185).

Zarur F.P.,dâ€™Almeida L.F.V.,Mafort M.S.P et al.Two cases of renal cell cancer during immunobiologic therapy for psoriasis.Anais Brasileiros de Dermatologia (2014) **89:** (1017-1018).

Wang S.-H.,Chi C.-C.,Hu S.Cost-efficacy of biologic therapies for moderate to severe psoriasis from the perspective of the taiwanese healthcare system. International Journal of Dermatology (2014) **53:(**1151-1156)

Messori A.,Fadda V.,Maratea D.Biological drugs for the treatment of moderate-to-severe psoriasis by subcutaneous route: Determining statistical equivalence according to evidence-based methods.Clinical Drug Investigation (2014) **34:** (593-598).

Gillespie T.Research evidence for reducing cardiovascular risk with biologic therapies in patients who have psoriasis.Journal of the Dermatology Nurses' Association (2014**) 6:**(142-147).

Purohit V.S.,Lamba M.,Gupta P.Pharmacometrics in dermatology.AAPS Advances in the Pharmaceutical Sciences Series (2014**) 14** (499-516).

C.,Richetta A.G.Costs of therapy with biologics in the treatment of moderate to severe plaque psoriasis in the

context of the Italian health-care system.Terranova L.,Mattozzi .Giornale Italiano di Dermatologia e Venereologia (2014) 149:(131-143).

Toussirot Ã‰.,Aubin F.,Dumoulin G. Relationships between adipose tissue and psoriasis, with or without arthritis.Frontiers in Immunology (2014) 5:AUG Article Number: Article 368.

Karczewski J.,PoniedziaÅ‚ek B.,Rzymski P. Factors affecting response to biologic treatment in psoriasis.Dermatologic Therapy (2014) **27:** (323-330).

Vaclavkova A.,Chimenti S.,Arenberger P et al. Oral ponesimod in patients with chronic plaque psoriasis: A randomised, double-blind, placebo-controlled phase 2 trial.The Lancet (2014) **384:** (2036-2045).

Higher risk of tuberculosis reactivation when anti-TNF is combined with immunosuppressive agents: A systematic review of randomized controlled trials.Lorenzetti R.Zullo A.,Ridola L.Annals of Medicine (2014) **46:** (547-554).

Boehncke W.H.,Qureshi A.,Merola J.F et al. Diagnosing and treating psoriatic arthritis: An update.British Journal of Dermatology (2014**) 170:** (772-786).

Birnbaum J,Bingham C.O.Non-length-dependent and length-dependent small-fiber neuropathies associated with tumor necrosis factor (TNF)-inhibitor therapy in patients with rheumatoid arthritis: Expanding the spectrum of neurological disease associated with TNF-inhibitors. Seminars in Arthritis and Rheumatism (2014) **43**: (638-647).

Puig L Biologic therapies for moderate to severe psoriasis are not interchangeable.Actas Dermo-Sifiliograficas (2014) **105:** (483-486).

Hunn B.H.M.,Martin W.G.,Simpson Jr. S et al. Idiopathic granulomatous hypophysitis: A systematic review of 82 cases in the literature. Pituitary (2014) **17**:357-65.

Chen Y.,Xin T.,Cheng A.S.K.Evaluating the effectiveness of psychological and/or educational interventions in psoriasis: A narrative review.Journal of Dermatology (2014) **41:** (775-778).

Lapadula G.,Marchesoni A.,Armuzzi Aet al.Adalimumab in the Treatment of Immune-Mediated Diseases.International Journal of Immunopathology and Pharmacology (2014) **27** Supplement 1 (33-48).

Coates L.C,Ritchlin C.T.,Kavanaugh A.F. GRAPPA treatment recommendations: An update from the GRAPPA 2013 annual meeting.Journal of Rheumatology (2014) **41:**(1237-1239).

Lee J.,Son C.,Lee J.A et al.Do herbal medicines negatively affect liver tests? A systematic review of Korean clinical studies on safety. European Journal of Integrative Medicine (2014**) 6: (**441-450)

Albert U.De Cori D.,Blengino G et al. Lithium treatment and potential long-term side effects: A systematic review of the literature. Rivista di Psichiatria (2014) **49**:(12-21).

Cinotti E.,Fouilloux B.,Perrot J.L et al. Confocal microscopy for healthy and pathological nail. Journal of the European Academy of Dermatology and Venereology (2014) **28:**(853-858).

Frendl D.M.,Ware J.E. Patient-reported functional health and well-being outcomes with drug therapy: A systematic review of randomized trials using the SF-36 health survey. Medical Care (2014) **52:** (439-445).

Karimkhani C.,Boyers L.N.,Prescott L et al.Global burden of skin disease as reflected in Cochrane Database of Systematic Reviews. JAMA Dermatology (2014) **150:**(945-951).

Yang Y., Zhang K., Zhou R. Meta-analysis of pre-miRNA polymorphisms association with susceptibility to autoimmune diseases. Immunological Investigations (2014**) 43:** (13-27).

De Wit M, Campbell W., FitzGerald Oet al.Patient participation in psoriasis and psoriatic arthritis outcome research: A report from the GRAPPA 2013 annual meeting. Journal of Rheumatology (2014) **41**: (1206-1211).

Hashkes P.J. ,Becker M.L. ,Cabral D.A et al. Methotrexate: New uses for an old drug.Journal of Pediatrics (2014) **164:**(231-236).

Vary J.C.,O'Connor K.M. Common dermatologic conditions. Medical Clinics of North America (2014**) 98**: (445-485).

Parkins G.,Wylie G. Guideline vs. practice in procollagen-3-aminopeptide monitoring. British Journal of Dermatology (2014) **171:** (1599-1600).

Selmi C.Unique topics and issues in rheumatology and clinical immunology.Clinical Reviews in Allergy and Immunology (2014) **47:**(1-5).

Cresce N.D.,Davis S.A.,Huang W.Wet al.The quality of life impact of acne and rosacea compared to other major medical conditions. Journal of Drugs in Dermatology (2014) **13:**(692-697).

Vassallo C.,Derlino F.,Brazzelli V et al Acute generalized exanthematous pustulosis: Report of five cases and systematic review of clinical and histopathological findings.Italiano di Dermatologia e Venereologia (2014) **149:** (281-290).

Hay R.J., Johns N.E., Williams H.C et al. The global burden of skin disease in 2010: An analysis of the prevalence and impact of skin conditions. Journal of Investigative Dermatology (2014) **134:** (1527-1534).

Liang Y.,Pan H.-F.,Ye D.-Q.Therapeutic potential of STAT4 in autoimmunity. Expert Opinion on Therapeutic Targets (2014) **18:** (945-960).

Katta R., Desai S.P. Diet and dermatology: The role of dietary intervention in skin disease. Journal of Clinical and Aesthetic Dermatology (2014) **7:** (46-51).

Iyer A, Elsone L., Appleton R et al. A review of the current literature and a guide to the early diagnosis of autoimmune disorders associated with neuromyelitis optica Autoimmunity (2014) **47:** (154-161).

Heyes C.,Tait C.,Toholka R et al. Non-infectious skin disease in Indigenous Australians. Australasian Journal of Dermatology (2014) **55**:(176-184).

MarÃ A., Morla A., Melero M et al. Diffuse sclerosing osteomyelitis (DSO) of the mandible in SAPHO syndrome: A novel approach with anti-TNF therapy. Systematic review. Journal of Cranio-Maxillofacial Surgery (2014**) 42: (**1990-1996).

Wootla B.,Denic A.,Rodriguez M.Polyclonal and monoclonal antibodies in clinic Methods in Molecular Biology (2014) 1060 (79-110).

Bruner V.,Atteno M.,SpanÃ² A Biological therapies for spondyloarthritis. Therapeutic Advances in Musculoskeletal Disease (2014**) 6: (**92-101).

Chen Y.,Lyga J.Brain-skin connection: Stress, inflammation and skin aging. Inflammation and Allergy - Drug Targets (2014) **13:** (177-190).

Gorouhi F., Maibach H. Evidence or experience: That is the question. American Journal of Clinical Dermatology (2014) **15:** (147-148).

Hsieh J., Kadavath S., Efthimiou P. Can traumatic injury trigger psoriatic arthritis? A review of the literature. Clinical Rheumatology (2014) **33:(**601-608).

Gremese E.,Tolusso B.,Gigante M.R et al.Obesity as a risk and severity factor in rheumatic diseases (autoimmune chronic inflammatory diseases.Frontiers in Immunology (2014) **5:**NOV Article Number: 576

Marotte H., Cimaz R. Etanercept - TNF receptor and IgG1 Fc fusion protein: Is it different from other TNF blockers?.Expert Opinion on Biological Therapy (2014) 14:5 (569-572).

Hu C., Wasfi Y., Zhuang Y et al. Information contributed by meta-analysis in exposure-response modeling: Application to phase 2 dose selection of guselkumab in patients with moderate-to-severe psoriasis.Journal of Pharmacokinetics and Pharmacodynamics (2014) **41:** (239-250).

De Castro Maymone M.B., Gan S.D., Bigby M. Evaluating the strength of clinical recommendations in the medical literature: GRADE, SORT, and AGREE. Journal of Investigative Dermatology (2014) **134:** (e25).

Murdaca G., Gulli R.,SpanÃ² F.TNF-Î± gene polymorphisms: Association with disease susceptibility and response to anti-TNF-Î± treatment in psoriatic arthritis. Journal of Investigative Dermatology (2014) **134:** (2503-2509).

P Tillett W., Adebajo A., Brooke M et al. Patient involvement in outcome measures for Psoriatic arthritis.Current Rheumatology Reports (2014) **16:** Article Number: 418.

Sun H., Xu B, Meng Q. et al. PSORS1C1/CDSN is associated with ankylosing spondylitis. Joint Bone Spine (2014) **81:**(268-272).

Wat H., Dytoc M. Off-label uses of topical vitamin D in dermatology: A systematic review.Journal of Cutaneous Medicine and Surgery (2014) **18:** (91-108).

Mimoso C., Lee D.-D., Zavadil J. et al. Analysis and meta-analysis of transcriptional profiling in human epidermis.Methods in Molecular Biology (2014) 1195 (61-97).

Felquer M.L.A., Coates L.C., Soriano E.R et al. Drug therapies for peripheral joint disease in psoriatic arthritis: A systematic review. Journal of Rheumatology (2014) **41**: (2277-2285).

Tian G.,Liang J.-N.,Wang Z.-Y. et al.Emerging role of leptin in rheumatoid arthritis. Clinical and Experimental Immunology (2014) **177:**(557-570).

Makara-StudzinÅka M.,Partyka I.,Ziemecki P et al .The occurrence of emotional problems in somatic diseases based on psychodermatology.Achives of Psychiatry and Psychotherapy (2014) **16:** (23-28).

Hamilton M.,Ntais D.,Griffiths C et al Value for money in treating psoriasis: **A systematic review** of full economic evaluations of psoriasis therapies..British Journal of Dermatology (2014) **171:**(e158).

Kitchen H.,Cordingley L.,Young H et al.Identifying patient-reported outcome measures for the clinical management of psoriasis.British Journal of Dermatology (2014) **171:** (e129).

Obradors M.,Figueras M.,Paz S et al.Costs of psoriasis in Europe**.** A systematic review of the literature.Value in Health (2014) 17:7 (A606).

Obradors M.,Figueras M.,Paz S et al. Factors conditioning health related quality of life in patients with psoriasis in europe: A systematic review of the literature. Value in Health (2014) **17:**(A612).

Souto A.,Maneiro J.R.,Salgado E. Et al.Risk of tuberculosis in patients with chronic immune-mediated inflammatory diseases treated with biologics and tofacitinib: a systematic review and meta-analysis of randomized controlled trials and long-term extension studies.Rheumatology (Oxford, England) (2014) **53:(**1872-1885).

Roubille C.,Richer V.,Starnino T. et al.The effects of TNF inhibitors, methotrexate, NSAIDS and corticosteroids on cardiovascular events in rheumatoid arthritis, psoriasis and psoriatic arthritis: A systematic review and meta-analysis. Annals of the Rheumatic Diseases (2014) 73 SUPPL. 2.

Low C., Conway R., Coughlan R.J. et al.Methotrexate use is not associated with an increased risk of lung disease: A meta-analysis of randomised controlled trials. Annals of the Rheumatic Diseases (2014) 73 SUPPL. 2.

McFarlane A.,Roubille C.,Richer V et al. Cardiovascular outcomes in patients with rheumatoid arthritis, psoriasis and psoriatic arthritis: A systematic review and meta-analyses. Annals of the Rheumatic Diseases (2014) 73 SUPPL. 2.

Smiechowski B.,Chen M.,Vieira M.C.Comparability of trial populations in network meta-analyses assessing biologic treatments in moderate to severe plaque psoriasis.Value in Health (2014) 17:3 (A224).

Karimkhani C.,Boyers L.N.,Dunnick C.A et al Representation of the top three most disabling skin diseases in the cochrane database of systematic reviews.Journal of Investigative Dermatology (2014) 134 SUPPL. 1 (S50).

Langley R.G.,Signorovitch J.,Wang K. et al.Number needed to treat and cost per responder for biologic therapies for the treatment of moderate to severe psoriasis. Journal of the American Academy of Dermatology (2014) 70:5 SUPPL. 1 (AB2).

Ceglowska U.,Wlodarczyk A.,Slomka M.Clinical effectiveness of fumaric acid esters (Fumaderm) in psoriasis: A systematic review of literature. Value in Health (2014) **17:** (A605)

2013

Richard M.-A.,Paul C.Cardiovascular morbidity, risk of cancer and alcohol abuse in psoriasis: Systematic literature reviews and expert opinion.Journal of the European Academy of Dermatology and Venereology (2013) 27:SUPPL.3 (1).

Dauden E.,Castaneda,Suarez C. et al.Clinical practice guideline for an integrated approach to comorbidity in patients with psoriasis.Journal of the European Academy of Dermatology and Venereology (2013) **27:(**1387-1404).

Brodszky V.Mo M.,Gulacsi L. et al Indirect comparison of the effect of biologics in patients with psoriasis. A meta-analysis of randomized, double blind clinical trials in bayesian framework. Value in Health (2013) **16:** (A501-A502).

Feldman S.R.,Burudpakdee C.,Gala S.,Mallya U.Systematic literature review of economic burden of chronic plaque psoriasis.Value in Health (2013) **16**:7 (A504).

Ncube B,Ndhlala A.R.,Okem A. et alHypoxis (Hypoxidaceae) in African traditional medicine. Journal of

Ethnopharmacology (2013) **150**:(818-827).

Suwandy F.,Van Der Meijden W.I.Smoking and the skin.Nederlands Tijdschrift voor Dermatologie en Venereologie (2013) **23:**(666-671).

Tzellos T.,Kyrgidis A.,Trigoni A.,Zouboulis C.C.Association of anti-IL-12/23 biologic agents ustekinumab and briakinumab with major adverse cardiovascular events. Journal of the European Academy of Dermatology and Venereology (2013) **27:**(1586-1587).

Brazzelli V., Grasso V., Borroni G. Imatinib, dasatinib and nilotinib: A review of adverse cutaneous reactions with emphasis on our clinical experience. Journal of the European Academy of Dermatology and Venereology (2013) **27:**(1471-1480).

Westerberg D.P.,Voyack M.J.O nychomycosis: Current trends in diagnosis and treatment.American Family Physician (2013) 88: (762-770).

Rashidi A, Fisher S.I. Therapy-related acute promyelocytic leukemia: A systematic review.Medical Oncology (2013) **30**:Article Number: 625.

Mulekar S.V., Isedeh P. Surgical interventions for vitiligo: An evidence-based review. British Journal of Dermatology (2013) **169**:SUPPL. 3 (57-66).

T Savage L.J., McGonagle D.G .The Role of Biological and Small Molecule Therapy in the Management of Psoriatic Arthritis.Biologics in Therapy (2013) 3: (61-81).

Bakewell C.J., Olivieri I., Aydin S.Z et al. Ultrasound and magnetic resonance imaging in the evaluation of psoriatic dactylitis: Status and perspectives.Journal of Rheumatology (2013) **40:** (1951-1957).

Manczinger M., KemÃny L. Novel factors in the pathogenesis of psoriasis and potential drug candidates are found with systems biology approach.PLoS ONE (2013) 8:11 Article Number: e80751.

Tian S., Suárez-Fariñas M. Multi-TGDR: A regularization method for multi-class classification in microarray experiments.PLoS ONE (2013) **8:**11 Article Number: e78302.

Remmers E.F., Kirino Y., Bertsias G et al Pediatric .PW03-011 New BehcÂ¸et's loci and gene-gene interactions. Rheumatology (2013) 11 SUPPL. 1.

Ioannidis J.P.A., Karassa F.B., Druyts E et al. Biologic agents in rheumatology: Unmet issues after 200 trials and $200 billion sales. Nature Reviews Rheumatology (2013) 9: (665-673).

Dols A.,Sienaert P.,Van Gerven H et al.The prevalence and management of side effects of lithium and anticonvulsants as mood stabilizers in bipolar disorder from a clinical perspective: A review. International Clinical Psychopharmacology (2013) **28:(**287-296).

Kupetsky E.A.,Keller M..Psoriasis vulgaris: An evidence-based guide for primary care. Journal of the American Board of Family Medicine (2013) **26:(**787-801).

Pan M.,Heinecke G.,Bernardo S et al.Urea: A comprehensive review of the clinical literature. Dermatology Online Journal (2013) **19:**11.

Asseburg C.,Valgardsson S.,Soini E.J.Cost-effectiveness of sequences of biologic treatments for moderate-to-severe psoriasis in Finland. Value in Health (2013) **16:** (A507-A508).

Valencia A., Hernandez A., Puig A. A cost-effectiveness model comparing sub-cutaneous biologic treatment for severe plaque psoriasis in Mexico. Value in Health (2013) **16:** (A726).

Riveros B.S.,Rotta I.,Garcia M et al.Multi-criteria benefit-risk assessment of biological agents in the treatment of moderate to severe psoriais: A stochastical approach. Value in Health (2013) **16**:(A727).

Betts K.A., Sundaram M., Mughal F et al. Cost-effectiveness of biologic therapies for the treatment of moderate to severe psoriasis in the United Kingdom. Value in Health (2013**) 16:** (A505-A506).

Ariza J.G.,NuÃ±ez C. A cost-effectiveness and budget impact analysis of different biologic treatments for psoriasis in Colombia. Value in Health (2013) **16:** (A696).

Lemos L.L.P., Reis C.A.L., Barbosa M.M et al. Treating psoriatic arthritis with biological disease modifying antirheumatic drugs: Systematic review and meta-analysis to evaluate efficacy and safety.Value in Health (2013) **16**: (A715).

Tseng H.W.,Lin H.S.,Lam H.C.Co-morbidities in psoriasis: A hospital-based case-control study. Journal of the European Academy of Dermatology and Venereology (2013) **27:**(1417-1425).

Poddubnyy D,Rudwaleit M.Adalimumab for the treatment of ankylosing spondylitis and nonradiographic axial spondyloarthritis - A five-year update. Expert Opinion on Biological Therapy (2013) 13:11 (1599-1611).

The Cochrane Database of Systematic Reviews - Issue 10 2013. Journal of Evidence-Based Medicine (2013**) 6: (**305-306).

AlGhamdi K.,Kumar A.,Moussa N. The role of vitamin D in melanogenesis with an emphasis on vitiligo.Indian Journal of Dermatology, Venereology and Leprology (2013) **79**:(750-758).

Gokhale S., Jalapu A., Mallya U., Mpofu S. Assessment of cost-effectiveness models for biologics in the management of psoriatic arthritis. Value in Health (2013**) 16**: (A717).

Lu Y., Kane S., Chen H et al The role of 39 psoriasis risk variants on age of psoriasis onset. ISRN Dermatology (2013) 2013 Article Number: 203941.

Barron A.J., Zaman N., Cole G.D. et al. Systematic review of genuine versus spurious side-effects of beta-blockers in heart failure using placebo control: Recommendations for patient information. International Journal of Cardiology (2013) **168:**(3572-3579).

Gaeta M., Castelvecchio S., Ricci C et al. Role of psoriasis as independent predictor of cardiovascular disease: A meta-regression analysis. International Journal of Cardiology (2013) **168:**(2282-2288).

Vemer P., Rutten-Van Malken M.P.M.H. The road not taken: Transferability issues in multinational trials. PharmacoEconomics (2013) **31:** (863-876)

Sandmann F.G., Franken M.G., Steenhoek A et al. Do reassessments reduce the uncertainty of decision making? Reviewing reimbursement reports and economic evaluations of three expensive drugs over time. Health Policy (2013) **112:** (285-296).

Haddad A., Somaily M., Johnson S.R et al. Psoriatic arthritis mutilans: Clinical and radiographic definitions. A systematic review. Arthritis and Rheumatism (2013) 65 SUPPL. 10 (S152).

Hunt L., Emery P. Etanercept in the treatment of rheumatoid arthritis. Expert Opinion on Biological Therapy (2013) 13:(1441-1450).

Tempark T., Lueangarun S., Chatproedprai S et al.Flood-related skin diseases: A literature review. International Journal of Dermatology (2013) **52:** (1168-1176).

Machet L., Samimi M., Delage M et al. Systematic review of the efficacy and adverse events associated with infliximab treatment of hidradenitis suppurativa in patients with coexistent inflammatory diseases. Journal of the American Academy of Dermatology (2013) **69:(**649-650).

KiliÃ§ E., KiliÃ G., AkgÃl O. The reported adverse effects related to biological agents used for the treatment of rheumatic diseases in Turkey.T urkish Journal of Rheumatology (2013) **28:**(149-162).

Castiblanco J., Arcos-Burgos M. ,Anaya J.-M. What is next after the genes for autoimmunity?BMC Medicine (2013) 11:1 Article Number: 197

Stolwijk C., van Tubergen A., Castillo-Ortiz J.D et al. Prevalence of extra-articular manifestations in patients with ankylosing spondylitis: A systematic review and meta-analysis. Annals of the Rheumatic Diseases (2013).

Saraceno R., Bavetta M., Zangrilli A et al. Adalimumab in the treatment of plaque-type psoriasis and psoriatic arthritis. Expert Opinion on Biological Therapy (2013) **13:** (1325-1334).

Makhija K., Karunakaran S. The role of inflammatory cytokines on the aetiopathogenesis of depression. Australian and New Zealand Journal of Psychiatry (2013) **47:** (828-839).

Huynh D., Kavanaugh A. Psoriatic arthritis: Current therapy and future directions. Expert Opinion on Pharmacotherapy (2013) **14:**(1755-1764).

Gajinov Z.T.,MatiÄ M.B., Duran V.D et al .Drug-related pityriasis rubra pilaris with acantholysis. Vojnosanitetski Pregled (2013) **70:** (871-873).

Mouthon L., Fermand J.-P., Gottenberg J.-E. Management of secondary immune deficiencies: What is the role of immunoglobulins?-Current Opinion in Allergy and Clinical Immunology (2013) **13:**SUPPL. 2 (S56-S67).

Neef H.C., Riebschleger M.P., Adler J et al. Meta-analysis: Rapid infliximab infusions are safe. Alimentary Pharmacology and Therapeutics (2013) **38:** (365-376)

Mrowietz U.,Domm S.Systemic steroids in the treatment of psoriasis: What is fact, what is fiction. Journal of the European Academy of Dermatology and Venereology (2013) **27:**(1022-1025).

Gan E.Y., Chong W.-S., Tey H.L. Therapeutic strategies in psoriasis patients with psoriatic arthritis: Focus on new agents. BioDrugs (2013) 27: (359-373).

Nast A, Sporbeck B., Rosumeck S et al. Which antipsoriatic drug has the fastest onset of action?-systematic review on the rapidity of the onset of action. Journal of Investigative Dermatology (2013) **133:** (1963-1970).

Famenini S., Wu J.J. Infliximab-induced psoriasis in treatment of Crohn's disease-associated ankylosing spondylitis: Case report and review of 142 cases. Journal of Drugs in Dermatology (2013) **12:** (939-943).

Segaert S,RÃ¸pke M. The biological rationale for use of vitamin D analogs in combination with corticosteroids for the topical treatment of plaque psoriasis. Journal of Drugs in Dermatology (2013) **12:** (e129-e137).

Ahn C.S., Gustafson C.J., Sandoval L.F et al. Cost effectiveness of biologic therapies for plaque psoriasis. American Journal of Clinical Dermatology (2013) **14:** (315-326).

Amerio P., Amoruso G., Bardazzi F et al. Detection and management of latent tuberculosis infections before biologic therapy for psoriasis. Journal of Dermatological Treatment (2013) **24:**(305-311).

Yao Q. Nucleotide-binding oligomerization domain containing 2: Structure, function, and diseases. Seminars in Arthritis and Rheumatism (2013) **43**:(125-130).

Sivamani R.K., Goodarzi H., Garcia M.S et al. Biologic therapies in the treatment of psoriasis: A comprehensive evidence-based basic science and clinical review and a practical guide to tuberculosis monitoring. Clinical Reviews in Allergy and Immunology (2013) **44:** (121-140).

Krnjevic Pezic G.,Tomic Babic L.,Ceovic R et al Report of the prevalence of metabolic syndrome in Croatian psoriasis patients.Journal of the European Academy of Dermatology and Venereology (2013) 27 SUPPL. 4 (33-34).

Dodds M.G., Salinger D.H., Mandema J et al Clinical trial simulation to inform phase 2: Comparison of concentrated vs. distributed First-in-patient study designs in Psoriasis. CPT: Pharmacometrics and Systems Pharmacology (2013) 2:7 Article Number: e58.

Paudyal V., Watson M.C., Sach T et al. Are pharmacy-based minor ailment schemes a substitute for other service providers? A systematic review. British Journal of General Practice (2013) **63:**(e472-e481).

Waters J.P., Pober J.S., Bradley J.R. Tumour necrosis factor and cancer. Journal of Pathology (2013) **230:** (241-248)

Laftah Z, Arkir Z, Agius E et al. Infliximab for hidradenitis suppurativa should we be measuring antibody levels?. British Journal of Dermatology (2013) 169 SUPPL. 1 (5-6).

Anjum N, Lee L.H., Haworth A et al. Dermatology on-call service: Is it really necessary? British Journal of Dermatology (2013) 169 SUPPL. 1 (34-35).

Lang U.E., Borgwardt S. Molecular mechanisms of depression: Perspectives on new treatment strategies. Cellular Physiology and Biochemistry (2013) 31:(761-777).

Denadai R.,Teixeira F.V., Steinwurz F et al. Induction or exacerbation of psoriatic lesions during anti-TNF-Î± therapy for inflammatory bowel disease: A systematic literature review based on 222 cases. Journal of Crohn's and Colitis (2013) **7:**(517-524).

Karim R., Sykakis E., Lightman S et al. Interventions for the treatment of uveitic macular edema: A systematic review and meta-analysis. Clinical Ophthalmology (2013) 7 (1109-1144).

Foltz I.N., Karow M., Wasserman S.M. Evolution and emergence of therapeutic monoclonal antibodies what cardiologists need to know. Circulation (2013) **127:** (2222-2230).

Lopez A., Billioud V., Peyrin-Biroulet C et al. Adherence to anti-TNF therapy in inflammatory bowel diseases: A systematic review. Inflammatory Bowel Diseases (2013) **19:**(1528-1533).

Schiff M. Subcutaneous abatacept for the treatment of rheumatoid arthritis. Rheumatology (United Kingdom) (2013) **52:**(986-997).

GrÃber U., Spitz J., Reichrath J et al. Vitamin D: Update 2013 - From rickets prophylaxis to general preventive healthcare. Dermato-Endocrinology (2013) **5:**(331-347).

Van Den Bosch F. How to treat psoriatic arthritis?.Annals of the Rheumatic Disease (2013) 71 SUPPL. 3.

Pride H.B.,Tollefson M.,Silverman R.What's new in pediatric dermatology?: Part II. Treatment. Journal of the American Academy of Dermatology (2013) **68**: (899.e1-899.e11).

Griffith J.W., Luster A.D. Targeting cells in motion: Migrating toward improved therapies. European Journal of Immunology (2013) 43:6 (1430-1435).

Stolwijk C., Van Tubergen A., Castillo-Ortiz J. et al. Prevalence of extra-articular manifestations in patients with ankylosing spondylitis: A systematic review and meta-regression analysis. Annals of the Rheumatic Diseases (2013) 72 SUPPL. 3.

Makri O.E., Georgalas I, Georgakopoulos C.D. Drug-induced macular edema. Drugs (2013) **73:**(789-802).

Gomez-Reino J.J. What's new: Psoriatic arthritis. Annals of the Rheumatic Diseases (2013) 72 SUPPL. 3.

Brenaut E., Barnetche T., Misery L. Alcohol consumption: Is it really a risk factor for psoriasis?. Journal of Dermatology (2013) 40:6 (508).

Ingham M., Ellis L., Bolge S. Number needed to treat, cost per responder and budget impact of TNF inhibitors in the treatment of psoriatic arthritis. Annals of the Rheumatic Disease (2013) 71 SUPPL. 3.

Stuart M.E., Shrite S.A., Gandra S.R. Systematic safety review of five biologic antirheumatic drugs. Annals of the Rheumatic Disease (2013) 71 SUPPL. 3.

Georgakopoulou E.A., Andreadis D., Arvanitidis E et al. Biologic agents and oral diseases - An update on clinical applications. Acta Dermatovenerologica Croatica (2013) **21:**(24-34).

Bettoli V., Zauli S., Virgili A. Retinoids in the chemoprevention of non-melanoma skin cancers: Why, when and how.Journal of Dermatological Treatment (2013) 24: (235-237).

Catena-Dell'Osso M. ,Rotella F., Dell'Osso A et al. Inflammation, Serotonin and Major Depression. Current Drug Targets (2013) **14:(**571-577).

Sawyer L, Samarasekera E.J., Wonderling D et al. Topical therapies for the treatment of localized plaque psoriasis in primary care: A cost-effectiveness analysis. British Journal of Dermatology (2013) **168:** (1095-1105).

Weigle N., Mcbane S.Psoriasis. American Family Physician (2013) **87:**(626-633).

Gamble R.,Gilchrest B.,Dellavalle R. The Journal of Investigative Dermatology's experience in its first six months on Facebook. Journal of Investigative Dermatology (2013) 133 SUPPL. 1 (S100).

Rosado-Buzzo A,Mould-Quevedo J.F.,Tang B.,Gutierrez-Ardila M.V. et al Burden of disease associated with psoriasis in Colombia.Value in Health (2013) 16:(A116).

Dobson R.,Giovannoni G.Autoimmune disease in people with multiple sclerosis and their relatives: A systematic review and meta-analysis.Journal of Neurology (2013) **260:**(1272-1285).

Asztalos M.L.,Heller M.M.,Lee E.S.,Koo J. The impact of emollients on phototherapy: A review. Journal of the American Academy of Dermatology (2013) **68:**(817-824).

Sikirica S.Accounting for partial responders in cost-effectiveness modeling of biologics for psoriasis.Value in Health (2013) 16:3 (A27-A28).

Salinger D.H.,Mandema J.W.,Newmark R.D.,Gibbs M.A. Model-based meta-analysis informs phase 3 head-to-head trial simulations of brodalumab and competitors in psoriasis.Journal of Pharmacokinetics and Pharmacodynamics (2013) **40:** SUPPL. 1 (S45-S46).

Rocha L.K.,Romitti R.,Shinjo S et al.Cutaneous manifestations and comorbidities in 60 cases of Takayasu arteritis.Journal of Rheumatology (2013) **40:**(734-738).

Inkeles M.,Modlin R.,Pellegrini M.Meta-analysis of skin disease microarray datasets: Diagnosis, classification and insight into pathogenesis.Journal of Investigative Dermatology (2013) 133 SUPPL. 1 (S141).

Dommasch E.D,Troxel A.B.,Gelfand J.M.Major cardiovascular events associated with anti-IL 12/23 agents: A tale of two meta-analyses.Journal of the American Academy of Dermatology (2013) **68:(**863-865).

Parkins G.,Burden A.D.What is the optimal topical treatment for limited plaque psoriasis?.British Journal of Dermatology (2013**) 168:** (925-926).

dos Santos F.K.,Oyafuso M.H.,Kiill C.P et al.Nanotechnology-based drug delivery systems for treatment of hyperproliferative skin diseases - a review.Current Nanoscience (2013) **9:** (159-167).

Casanova Estruch B.Safety profile and practical considerations of monoclonal antibody treatment.Neurologia (2013) **28:** (169-178).

Ayer J.,Young H.S.Pimecrolimus for psoriasis.Expert Opinion on Pharmacotherapy (2013) **14:** (767-774).

Kannan S.,Heller M.M.,Lee E.S.,Koo J.Y.The role of tumor necrosis factor-alpha and other cytokines in depression: What dermatologists should know.Journal of Dermatological Treatment (2013) **24:** (148-152).

Flint J.,Gayed M.,Schreiber et al.A systematic analysis of the safety of prescribing of anti-rheumatic, immunosuppressive and biologic drugs in men trying to conceive.Rheumatology (United Kingdom) (2013) 52 SUPPL. 1 (i149).

Furst D.E.,Fleischman R.,Kalden J et al. Documentation of off-label use of biologics in Rheumatoid Arthritis.Annals of the Rheumatic Diseases (2013) 72:SUPPL. 2 (ii35-ii51).

Herrinton L.J.,Harrold L.R.,Liu L et al .Association between anti-TNF-Î± therapy and interstitial lung disease.Pharmacoepidemiology and Drug Safety (2013) 22: (394-402).

SokoÅ‚owska-WojdyÅ‚o M.,Åugowska-Umer H.,MacIejewska-Radomska A.Oral retinoids and rexinoids in cutaneous T-cell lymphomas.Postepy Dermatologii i Alergologii (2013) **30:**(19-29).

CÃrdenas-RoldÃ¡n J.,Rojas-Villarraga A.,Anaya J.-M.How do autoimmune diseases cluster in families? A systematic review and meta-analysis.BMC Medicine (2013) 11:1 Article Number: 73.

Kaffenberger B.H.,Wong H.K.,Jarjour W et al .Remission of psoriasis after allogeneic, but not autologous, hematopoietic stem-cell transplantation.Journal of the American Academy of Dermatology (2013) **68:** (489-492).

Harsono D.,Finelt N.,Castano E.B.Curcumin myths or wonders? A systematic analysis of in vitro studies.Wound Repair and Regeneration (2013) 21:2 (335-336).

Flatz L,Conrad C.Role of T-cell-mediated inflammation in psoriasis: Pathogenesis and targeted therapy.Psoriasis: Targets and Therapy (2014) 4 (1-10).

Jani M.,Chinoy H,Lamb J.A et al.Investigation of idiopathic inflammatory myopathy for shared genetic risk factors with other autoimmune diseases: Results from the european myositis network.The Lancet (2013) 381

SUPPL. 1 (S56).

Quiu Z.-X.,Zhang K.,Qiu X.-S et al. CD226 Gly307Ser association with multiple autoimmune diseases: A meta-analysis.Human Immunology (2013) 74:2 (249-255).

Tablazon I.L.D.,Al-Dabagh A.,Davis S.A.,Feldman S.R. Risk of cardiovascular disorders in psoriasis patients: Current and future.

Enamandram M.,Kimball A.B. Psoriasis epidemiology: The interplay of genes and the environment.Journal of Investigative Dermatology (2013) **133:**(287-289).

Blok J.L.,Van Hattem S.,Jonkman M.F. et al. Systemic therapy with immunosuppressive agents and retinoids in hidradenitis suppurativa: A systematic review.British Journal of Dermatology (2013) **168:** (243-252).

Ladizinski B.,Heller M.M.,Bhutani T et al. Progressive multifocal leukoencephalopathy and reversible progressive leukoencephalopathy syndrome in dermatologic therapy.Journal of Drugs in Dermatology (2013) **12:(**e20-e24).

Kirino Y.,Bertsias G.,Ishigatsubo Y.et al. Genome-wide association analysis identifies new susceptibility loci for BehÃ§et's disease and epistasis between HLA-B*51 and ERAP1.Nature Genetics (2013) **45:** (202-207).

Jamnitski A.,Symmons D, Peters M.J.L et al. Cardiovascular comorbidities in patients with psoriatic arthritis: A systematic review.Annals of the Rheumatic Diseases (2013) **72**: (211-216).

H¶nigsmann H. Synergism between narrowband ultraviolet B phototherapy and etanercept for the treatment of plaque-type psoriasis.British Journal of Dermatology (2013) **169:**(4-5).

DÃvila-Fajardo C.L.,Swen J.J.,Cabeza Barrera J et al. Genetic risk factors for drug-induced liver injury in rheumatoid arthritis patients using low-dose methotrexate.Pharmacogenomics (2013) **14:** (63-73).

Wang X.-Q.,Zhang K.,Xiong D. Toll-like receptor 4 polymorphisms and susceptibility to multiple autoimmune diseases: Evidence based on pooled analysis.Central-European Journal of Immunology (2013) 38:(380-387).

Gisondi P.,Girolomoni G.Impact of TNF-Î± antagonists on the quality of life in selected skin diseases. Giornale Italiano di Dermatologia e Venereologia (2013) **148:** (243-248).

Wong H.K.Novel biomarkers, dysregulated epigenetics, and therapy in cutaneous t-cell lymphoma. Discovery Medicine (2013) **16:** (71-78).

Moon H.-S.,Mizara A.,McBride S.R.Psoriasis and psycho-dermatology. Dermatology and Therapy (2013) **3:** (117-130).

Takeuchi M. A systematic review of biologics for the treatment of noninfectious uveitis. Immunotherapy (2013) **5:** (91-102).

Mortazavi H,Aghazadeh N.,Ghiasi M et al.A review of three systemic retinoids in dermatology: Acitretin, isotretinoin and bexarotene. Iranian Journal of Dermatology (2013) **16:** (144-158).

Gysin C.Indications of pediatric tonsillectomy. ORL (2013) 75:3 (193-202).

Muftin Z.,Thompson A.R.A systematic review of self-help for disfigurement: Effectiveness, usability, and acceptability. Body Image (2013) **10:**(442-450).

Wong J.W., Nguyen T.V., Koo J.Y.M. Primary psychiatric conditions: Dermatitis artefacta, trichotillomania and

neurotic excoriations. Indian Journal of Dermatology (2013) 58: (44-48).

Petrof G., Almaani N., Archer C.B. et al. A systematic review of the literature on the treatment of pityriasis rubra pilaris type 1 with TNF-antagonists. Journal of the European Academy of Dermatology and Venereology (2013) **27:** (e131-e135).

Frendl D.M.,Strom M.,Ware Jr. J.E.Patient reported health outcomes from well-controlled trials of biologic therapies: A systematic review.Pharmacoepidemiology and Drug Safety (2013) 22 SUPPL. 1 (436-437).

Song G.G.,Kim J.-H.,Lee Y.H.Association between the LCE3C-LCE3B deletion polymorphism and susceptibility to psoriasis**:** A meta-analysis of published studies.Genetic Testing and Molecular Biomarkers (2013) **17:**(572-577).

Liu T.,Han Y.,Lu L.Angiotensin-converting enzyme gene polymorphisms and the risk of psoriasis: A meta-analysis. Clinical and Experimental Dermatology (2013) 38:(352-359).

Varying prevalence of depression in psoriasis according to assessment method: A systematic review and meta-analysis.Dowlatshahi E.A.,Wakkee M.,Arends L.,Nijsten T.Journal of Investigative Dermatology (2013) 133 SUPPL. 1 (S99).

Foulkes A.C.,Jorgensen A.,Pirmohamed M et al.Systematic review of pharmacogenomics in psoriasis.Journal of Investigative Dermatology (2013) 133 SUPPL. 1 (S88).

Armstrong A.W.,Harskamp C.T.,Armstrong E.J.Psoriasis and metabolic syndrome: A systematic review and meta-analysis of observational studies. Journal of the American Academy of Dermatology (2013) **68:**(654-662).

Effectiveness and safety of compound glycyrrhizin combined with acitretin for psoriasis: A systematic review.Zhang D.-Y,Ren W.-M.,Shi C.-R. et al. Chinese Journal of Evidence-Based Medicine (2013) **13:**(112-120).

2012

Kaur A., Kumar S.Plants and plant products with potential antipsoriatic activity - A review. Pharmaceutical Biology (2012) **50:**(1573-1591).

Sorin D., Pavlovsky L., David M. Psoriasis in Pregnancy. Current Dermatology Reports (2012) **1: (**209-213).

Vena G.A., Cassano N., Piaserico S. et al. Efficacy of etanercept for the treatment of psoriasis: An overview of the Italian clinical experience from the real-life setting and independent studies. Immunopharmacology and Immunotoxicology (2012) **34:** (901-906).

Silva F, Cisternas M., Specks U. TNF-Î± blocker therapy and solid malignancy risk in ANCA-associated vasculitis. Current Rheumatology Reports (2012) **14:** (501-508)

Lakatos P.L. Anti-TNFs and severe infections in autoimmune diseases: The other side of the coin.Inflammatory. Bowel Diseases (2012) **18:** (2414-2416).

Nordgaard-Lassen I.,Dahlerup J.F.,Belard E.Guidelines for screening, prophylaxis and critical information prior to initiating anti-TNF-alpha treatment.Danish Medical Journal (2012) **59**:7.

Tsoi L.C., Spain S.L., Knight J. et al. Identification of 15 new psoriasis susceptibility loci highlights the role of innate immunity. Nature Genetics (2012) **44:**(1341-1348).

Zheng J., Ibrahim S., Petersen F. Meta-analysis reveals an association of PTPN22 C1858T with autoimmune diseases, which depends on the localization of the affected tissue. Genes and Immunity (2012) **13:**(641-652).

Wong P.C.H.,Leung Y.-Y.,Li E.K. et al .Measuring disease activity in psoriatic arthritis. International Journal of Rheumatology (2012) 2012 Article Number: 839425.

Voulgari P.V., Kaltsonoudis E., Papagoras C et al. Adalimumab in the treatment of rheumatoid arthritis. Expert Opinion on Biological Therapy (2012) **12:** (1679-1686)

Thorlund K., Druyts E, AviÃ±a-Zubieta J.A et al. Anti-tumor necrosis factor (TNF) drugs for the treatment of psoriatic arthritis: An indireccomparison meta-analysis. Biologics: Targets and Therapy (2012) 6 (417-427).

Olyaeemanesh A., Doaee S., Nejati M et al. Effectiveness and safety of Etanercept in treatment of arthritis. HealthMED (2012) **6:** (2709-2716).

Lavda A.C., Webb T.L.,Thompson A.R. A meta-analysis of the effectiveness of psychological interventions for adults with skin conditions. British Journal of Dermatology (2012) **167:**(970-979).

Valencia-Mendoza A., HernÃ¡ndez-GarduÃ±o A., Puig A. Cost-effectiveness of ustekinumab in the management of moderate-to-severe plaque psoriasis in Mexico. Value in Health (2012) **15:** (A513).

Dao K.H., Herbert M., Habal N et al. Nonserious Infections. Should There Be Cause for Serious Concerns? Rheumatic Disease Clinics of North America (2012) **38:** (707-725).

Marcellusi A., Gitto L., Giannantoni P et al. Economic evaluation of biologic treatments for moderate to severe psoriasis in Italy. Value in Health (2012) **15:** (A570).

Baughman R.P., Meyer K.C., Nathanson I. et al. Monitoring of nonsteroidal immunosuppressive drugs in patients with lung disease and lung transplant recipients: American College of Chest Physicians evidence-based clinical practice guidelines. Chest (2012) **142:** (e1S-e111S).

Granados D, Depont F., Chevreul K. Effect of the anti-tumor necrosis factor adalimumab on work productivity in patients with chronic immune-mediated inflammatory diseases: Literature review. Value in Health (2012) 15:7 (A511).

Farrell J., Mills E., Sheppard O.,Thorlund K. Anti-tumour necrosis factor (TNF) drugs for the treatment of psoriatic arthritis (PSA). Value in Health (2012) **15:** (A440).

Betts K., Yan Y., Sundaram M et al. Cost-effectiveness of biologic therapies for the treatment of moderate to severe psoriasis in Germany. Value in Health (2012) **15:** (A570).

Woods M.S., Zimovetz E., Beard S et al. Systematic review of economic evaluations, utility estimates, resource utilisation, and costs in chronic idiopathic urticarial. Value in Health (2012) **15:** (A562).

Wang X., Bansback N., Anis A et al. Economic evaluation model of biologic therapies for moderate to severe psoriatic arthritis in Germany. Value in Health (2012) **15:**(A446).

Jostins L, Ripke S., Weersma R.K. et al. Host-microbe interactions have shaped the genetic architecture of inflammatory bowel disease. Nature (2012) 491:7422 (119-124).

Fischer A., Schmid B., Ellinghaus D. A novel sarcoidosis risk locus for europeans on chromosome 11q13.1. American Journal of Respiratory and Critical Care Medicine (2012) **186:**(877-885).

Tan X., Feldman S.R.,Chang J et al.Topical drug delivery systems in dermatology: A review of patient adherence

issues. Expert Opinion on Drug Delivery (2012) **9:** (1263-1271).

Pereira F.R.A.,Basra M.K.A.,Finlay A.Y et al.The role of the EQ-5D in the economic evaluation of dermatological conditions and therapies. Dermatology (2012) **225:** (45-53).

Katz U.,Shoenfeld Y.,Zakin V et al.Scientific Evidence of the Therapeutic Effects of Dead Sea Treatments: A Systematic Review.Seminars in Arthritis and Rheumatism (2012) **42:** (186-200).

Selvarajah V.,Montano-Loza A.J.,Czaja A.J.Systematic review: Managing suboptimal treatment responses in autoimmune hepatitis with conventional and nonstandard drugs.Alimentary Pharmacology and Therapeutics (2012) **36:**(691-707).

Remmers E.F.,Kirino Y.,Bertsias G.et al.Genome-wide analysis reveals a recessive association of ERAP1 variants with behcÂ¸et's disease and epistasis between ERAP1 and HLA-Bâˆ—51.Arthritis and Rheumatism (2012) 64 SUPPL. 10 (S428).

E.,Akhmetova V.,Latipov B.Analysis of genetic variants of class II cytokine and their receptor genes in psoriasis patients of two ethnic groups from the Volga-Ural region of Russia. Journal of Dermatological Science (2012) **68:** (9-18).

Stuart P.E.,HÃffmeier U.,Nair R.P et al.Association of Î²-defensin copy number and psoriasis in three cohorts of European origin.Journal of Investigative Dermatology (2012) **132:** (2407-2413).

Akkineni R.,Albert D.A.Is there an optimal treatment strategy for disease-modifying-antirheumatic-drug naÃ¯ve patients with rheumatoid arthritis?. Arthritis and Rheumatism (2012) 64 SUPPL. 10 (S403).

Trembath R.C.,Abecasis G.,Elder J.T. et al The identification of fifteen novel psoriasis susceptibility loci highlights the skin's role in innate immune defense. Journal of Investigative Dermatology (2012) 132 SUPPL. 2 (S95).

Liang Y.-L.,Wu H.,Shen X. et al.Association of STAT4 rs7574865 polymorphism with autoimmune diseases: A meta-analysis. Molecular Biology Reports (2012) **39:** (8873-8882).

Wood A.Genome wide association studies (GWAS) for height variations: Where are we now?. Hormone Research in Paediatrics (2012) 78 SUPPL. 1 (8).

Tang T.,Bieber T.,Williams H.Does âœautoreactivityâ play a role in atopic dermatitis?.Journal of Investigative Dermatology (2012) 132 SUPPL. 2 (S34).

Augustin M.,Langenbruch A.K.,Gutknecht M et al.Quality of Life Measures for Dermatology: Definition, Evaluation, and Interpretation.Current Dermatology Reports (2012) **1:** (148-159).

Marino A.,Pagnini I.,Savelli S. et al.Elbow monoarthritis: An atypical onset of juvenile idiopathic arthritis.Reumatismo (2012) **64:** (175-179).

DaÅ„czak-Pazdrowska A.Place of methotrexate in the treatment of psoriasis in the era of biologic agents.Postepy Dermatologii i Alergologii (2012) **29:** (182-188).

Kamangar F.,Neuhaus I.M.,Koo J.Y.M.An evidence-based review of skin cancer rates on biologic therapies. Journal of Dermatological Treatment (2012) 23:(305-315).

Perk J.,De Backer G.Gohlke H et alEuropean Guidelines on cardiovascular disease prevention in clinical practice (version 2012): The Fifth Joint Task Force of the European Society of Cardiology and Other Societies on Cardiovascular Disease Prevention in Clinical Practice (constituted by representatives of nine societies and

by invited experts). European Journal of Preventive Cardiology (2012) **19:** (585-667).

Bessissow T.,Renard M.,Hoffman I. et alReview article: Non-malignant haematological complications of anti-tumour necrosis factor alpha therapy.Alimentary Pharmacology and Therapeutics (2012) **36**:(312-323).

Garcia-Doval I.,Rustenbach S.,Norman Dam T. Heterogeneity of patients treated with biologics in Psonet countries.British Journal of Dermatology (2012) **167:** (e18-e19).

Famenini S.,Wu J.J.The safety of ustekinumab in psoriasis. Journal of Drugs in Dermatology (2012**) 11: (**907-910).

Spuls P.H.I.Critical appraisal of treatment guidelines for psoriasis. British Journal of Dermatology (2012) 167: (e5-e6).

Kim I.H.,West C.E.,Kwatra S.G et al.Comparative efficacy of biologics in psoriasis: A review. American Journal of Clinical Dermatology (2012) **13:**(365-374).

Zhu K.-J.,Zhu C.-Y.,Shi G. et al.Association of IL23R polymorphisms with psoriasis and psoriatic arthritis: A meta-analysis. Inflammation Research (2012) 61:10 (1149-1154).

Tian S.,Krueger J.G.,Li K et al.Meta-Analysis Derived (MAD) Transcriptome of Psoriasis Defines the Core Pathogenesis of Disease.PLoS ONE (2012) 7:9 Article Number: e44274.

Sawchuk M.,Spano F.,Loo W.J et al. The coexistence of psoriasis and vitiligo: A review. Journal of Cutaneous Medicine and Surgery (2012) **16:(**300-305).

Kwatra S.G.,Dabade T.S.,Gustafson C.J et al.JAK inhibitors in psoriasis: A promising new treatment modality. Journal of Drugs in Dermatology (2012) 11:(913-918).

Chen Y.-F.,Chang J.S. PTPN22 C1858T and the risk of psoriasis: A meta-analysis. Molecular Biology Reports (2012) 39:8 (7861-7870).

Associations between interleukin-10 polymorphisms and susceptibility to psoriasis**:** A meta-analysis.Lee Y.H.,Choi S.J.,Ji J.D. et al. Inflammation Research (2012) 61:(657-663).

Zhu K.,Yin X.,Tang X Meta-Analysis of NOD2/CARD15 polymorphisms with psoriasis and psoriatic arthritis. Rheumatology International (2012) 32:7 (1893-1900).

Lee Y.H.,Choi S.J.,Ji J.D et al. Vitamin D receptor ApaI, TaqI, BsmI, and FokI polymorphisms and psoriasis susceptibility**:** A meta-analysis. Molecular Biology Reports (2012) 39:(6471-6478).

Thyssen J.P., Johansen J.D., Carlsen B.C. et al. The filaggrin null genotypes R501X and 2282del4 seem not to be associated with psoriasis: Results from general population study and meta-analysis. Journal of the European Academy of Dermatology and Venereology (2012) 26:(782-784).

Capon F.,Barker J.N.W.N.The quest for psoriasis susceptibility genes in the postgenome-wide association studies era: Charting the road ahead.British Journal of Dermatology (2012) 166:(1173-1175).

Tian S., Krueger J.G., Jabbari A. et al. Meta-analysis reveals 'global' psoriasis transcriptome with links to cardiovascular, metabolic and other comorbidities. Journal of Investigative Dermatology (2012) 132 SUPPL. 1 (S8).

Mudigonda T.,Dabade T.S.,Feldman S.R.A review of targeted ultraviolet B phototherapy for psoriasis. Journal of the American Academy of Dermatology (2012) **66:(**664-672).

May B.H.,Zhang A.L.,Zhou W et al.Oral herbal medicines for psoriasis: A review of clinical studies. Chinese Journal of Integrative Medicine (2012) **18:** (172-178).

Cheng J.,Kuai D.,Zhang L et al.Psoriasis increased the risk of diabetes**:** A meta-analysis. Archives of Dermatological Research (2012) **304:** (119-125).

Mudigonda T.,Dabade T.S.,Feldman S.R. A Review of protocols for 308 nm excimer laser phototherapy in psoriasis. Journal of Drugs in Dermatology (2012) **11:** (92-97).

Heo J.,Sepah Y.J.,Yohannan J et al.The role of biologic agents in the management of non-infectious uveitis. Expert Opinion on Biological Therapy (2012) **12:** (995-1008).

Tang T.S.Bieber T.,Williams H.C.Does 'autoreactivity' play a role in atopic dermatitis?. British Journal of Dermatology (2012) 167: (e16).

Wain L.V.,Artigas M.S.,Tobin M.D.What can genetics tell us about the cause of fixed airflow obstruction? Clinical and Experimental Allergy (2012) **42:** (1176-1182).

Hayter S.M.,Cook M.C.Updated assessment of the prevalence, spectrum and case definition of autoimmune disease. Autoimmunity Reviews (2012) 11:10 (754-765).

Kydd A.,March L.Smoking and ankylosing spondylitis: A systematic review. Rheumatology (2012) **39:** (1710-1711).

Liu Y.,Wu E.Q.,Bensimon A.G et al Cost per responder associated with biologic therapies for crohn's disease, psoriasis, and rheumatoid arthritis"""

Biberoglu K.Prevention of endometriosis: Is it possible?.Journal of Endometriosis (2012) **4:** (129-130).

Tzellos T.,Kyrgidis A.,Trigoni A.,Zouboulis C.C.Association of ustekinumab and briakinumab with major adverse cardiovascular events: An appraisal of meta-analyses and industry sponsored pooled analyses to date.Dermato-Endocrinology (2012) **4:**3.

Calkin C.,Alda M.Beyond the guidelines for bipolar disorder: Practical issues in long-term treatment with lithiumCanadian Journal of Psychiatry (2012) **57:** (437-445).

Page E.K., Dar W.A., Knechtle S.J. Biologics in organ transplantation. Transplant International (2012) **25:** (707-719).

Lucka T.C,Pathirana D.,Sammain A.Erratum: Efficacy of systemic therapies for moderate-to-severe psoriasis: A systematic review and meta-analysis of long-term treatment. Journal of the European Academy of Dermatology and Venereology (2012) 26:7 (930).

Maes M.,Berk M.,Goehler LDepression and sickness behavior are Janus-faced responses to shared inflammatory pathways. BMC Medicine (2012) 10 Article Number: 66.

Singh S.,Mann B.K.Clinical utility of clocortolone pivalate for the treatment of corticosteroid-responsive skin disorders: A systematic review.Clinical, Cosmetic and Investigational Dermatology (2012**) 5** (61-68).

Micha R.,Imamura F.,Wyler Von Ballmoos M. Authors' replyAmerican Journal of Cardiology (2012) 109: (1823).

Nast A.,Boehncke W.-H.,Mrowietz U S3 - Guidelines on the treatment of psoriasis vulgaris (English version).

Update.DDG - Journal of the German Society of Dermatology (2012) 10:SUPPL.2 (S1-S95).

Bigby M.The use of anti-interleukin-12/23 agents and major adverse cardiovascular events.Archives of Dermatology (2012) 148: (753-754).

M.,Ellis L.,Bolge S. Ingham. Number needed to treat (NNT), cost per responder (CPR) and budget impact (BI) of tnf inhibitors (TNFI) in the treatment of psoriatic arthritis (PSA).Value in Health (2012) 15:4 (A35).

FerrÃndiz C., Garcia A., Blasco A. J et a.l Cost-efficacy of adalimumab, etanercept, infliximab and ustekinumab for moderate-to-severe plaque psoriasis.Journal of the European Academy of Dermatology and Venereology (2012) **26:** (768-777).

Armesto S., Coto-Segura P, Osuna C.G. Psoriasis and hypertension: A case-control study. Journal of the European Academy of Dermatology and Venereology (2012) 26:6 (785-788).

Jordan C.T.,Cao L.,Roberson E.D.O.Rare and common variants in CARD14, encoding an epidermal regulator of NF-kappaB, in psoriasisAmerican Journal of Human Genetics (2012) **90:** (796-808).

Almalag H.M., Mangoni A.A., Crilly M.A. Methotrexate and risk of cardiovascular disease.American Journal of Cardiology (2012) 109:9 (1383-1384).

Tang T.S., Bieber T., Williams H.C. Does autoreactivity play a role in atopic dermatitis?.Journal of Allergy and Clinical Immunology (2012) 129: (1209-1215).

Cho J.H. Immunochip-based analysis of a large ibd case-control cohort identifies 50 novel loci, refining definitions of disease pathways. Gastroenterology (2012) 142:5 SUPPL. 1 (S149-S150).

Banks T., Gada S. A comprehensive review of current treatments for granulomatous cheilitis. British Journal of Dermatology (2012) 166:5 (934-937).

Cifuentes R.A., Restrepo-Montoya D., Anaya J.-M. The autoimmune tautology: An in silico approach. Autoimmune Diseases (2012) 1:1 Article Number: 792106.

Denadai R.,Teixeira F.V.,Saad-Hossne R. The onset of psoriasis during the treatment of inflammatory bowel diseases with infliximab: Should biological therapy be suspended?. Arquivos de Gastroenterologia (2012) **49:** (172-176).

Holick M.F. Evidence-based D-bate on health benefits of vitamin D revisited. Dermato-Endocrinology (2012) 4:2 (183-190).

Basra M.K.A., Chowdhury M.M.U., Smith E.V.A. Review of the Use of the Dermatology Life Quality Index as a Criterion in Clinical Guidelines and Health Technology Assessments in Psoriasis and Chronic Hand Eczema. Dermatologic Clinics (2012) **30:**(237-244).

Ellinghaus E., Stuart P.E., Ellinghaus et al. Genome-wide meta-analysis of psoriatic arthritis identifies susceptibility locus at REL. Journal of Investigative Dermatology (2012) **132:**(1133-1140).

Yang H., Craig D., Epstein D et al. Golimumab for the treatment of psoriatic arthritis: A NICE single technology appraisal. PharmacoEconomics (2012) 30:4 (257-270).

Nijsten T., Stern R.S. How epidemiology has contributed to a better understanding of skin disease. Journal of Investigative Dermatology (2012) 132:3 PART 2 (994-1002).

Williams H.C., Dellavalle R.P. The growth of clinical trials and systematic reviews in informing dermatological patient care. Journal of Investigative Dermatology (2012) **132:** PART 2 (1008-1017).

Pham T., Bachelez H., Berthelot J.-M. Abatacept therapy and safety management. Joint Bone Spine (2012) **79:**SUPPL. 1 (3-84).

Tejasvi T., Stuart P.E., Chandran V et al. TNFAIP3 gene polymorphisms are associated with response to TNF blockade in psoriasis. Journal of Investigative Dermatology (2012) 132: PART 1 (593-600).

Atzeni F., Sarzi-Puttini P. Twelve years experience with etanercept in the treatment of rheumatoid arthritis: How it has changed clinical practice. Expert Review of Clinical Immunology (2012**) 8:(**213-222).

Bergboer J.G.M. ,UmiÄeviÄ-Mirkov M. ,Fransen J et al. A replication study of the association between rheumatoid arthritis and deletion of the Late Cornified Envelope genes LCE3B and LCE3C. PLoS ONE (2012) **7:**2 Article Number: e32045.

Croxtall J.D. Spotlight on Ustekinumab in moderate to severe plaque psoriasis. American Journal of Clinical Dermatology (2012) **13:**(135-137).

De Mozzi P., Johnston G.A., Alexandroff A.B. Psoriasis: An evidence-based update. Report of the 9th Evidenced Based Update Meeting, 12 May 2011, Loughborough, U.K. British Journal of Dermatology (2012) **166:** (252-260).

Li X., Ampleford E., Howard T et al.Genome-wide association studies of asthma indicate opposite immunopathogenesis direction from autoimmune diseases. World Allergy Organization Journal (2012) 5 SUPPL. 2 (S55-S56).

Graeber K.E., Olsen N.J. Th17 cell cytokine secretion profile in host defense and autoimmunity. Inflammation Research (2012) **61:**(87-96).

Tillett W., de-Vries C., McHugh N.J. Work disability in psoriatic arthritis: A systematic review. Rheumatology (2012) 51:2 (275-283) Article Number: ker216.

ZÃller B., Li X., Sundquist J et al. Risk of pulmonary embolism in patients with autoimmune disorders: A nationwide follow-up study from Sweden. The Lancet (2012) 379:9812 (244-249).

Guerini F.R., Cagliani R., Forni D. A functional variant in ERAP1 predisposes to multiple sclerosis. PLoS ONE (2012) 7:1 Article Number: e29931.

Balak D.M., Hengstman G.J., Ãakmak A. Cutaneous adverse events associated with disease-modifying treatment in multiple sclerosis: A systematic review.Multiple Sclerosis Journal (2012) 18:(1705-1717).

Gensicke H.,Leppert D.,Yaldizli O. Monoclonal antibodies and recombinant immunoglobulins for the treatment of multiple sclerosis. CNS Drugs (2012) **26:** (11-37).

Bogaczewicz A.,SobÃ³w T.,Bogaczewicz J. et al.(Meta-analysis of the usage of biologics for depressive symptoms in patients with psoriasis(2012) **14:(**53-57).

Dowlatshahi E.A.,Van Der Voort E.A.,Arends L et al.Markers of systemic inflammation in psoriasis: A systematic review and meta-analysis .Journal of Investigative Dermatology (2012) 132 SUPPL. 2 (S71).

Bae Y.-S.C.,Van Voorhees A.S.,Hsu S. et al.Review of treatment options for psoriasis in pregnant or lactating women: From the Medical Board of the National Psoriasis Foundation. Journal of the American Academy of Dermatology (2012) **67:(**459-477).

Jemec G.B.E.,Ibler K.S.Treatment of nail psoriasis with TNF-Î± or IL 12/23 inhibitors. Journal of Drugs in Dermatology (2012) **11:**(939-942).

Deng S.-Q.,Brian M.,Tony Z,. et al.Herbal medicines in the topical management of psoriasis: A systematic review of clinical evidence. Journal of Dermatology (2012) 39 SUPPL. 1 (226-227).

Paul C.,Ortonne J.-P.   treatment and phototherapy in psoriasis: Systematic review and expert opinion of a panel of dermatologists. Journal of the European Academy of Dermatology and Venereology (2012) 26:SUPPL. 3 (iii-iv).

Bailey E.E.,Ference E.H.,Alikhan A et al. Combination treatments for psoriasis: A systematic review and meta-analysis. Journal of Investigative Dermatology (2010) 130 SUPPL. 1 (S52)

Robinson A.,Kardos M.,Kimball A.BPhysician Global Assessment (PGA) and Psoriasis Area and Severity Index (PASI): Why do both? A systematic analysis of randomized controlled trials of biologic agents for moderate to severe plaque psoriasis. Journal of the American Academy of Dermatology (2012) **66:(**369-375).

Dauden E.,Castaneda S.,Suarez C et al.Integrated approach to comorbidity in patients with psoriasis.Actas Dermo-Sifiliograficas (2012) 103:SUPPL. 1 (1-64).

2011

Cullen G.,Kroshinsky D.,Cheifetz A.S et al. Psoriasis associated with anti-tumour necrosis factor therapy in inflammatory bowel disease: A new series and a review of 120 cases from the literature. Alimentary Pharmacology and Therapeutics (2011) **34:** (1318-1327).

Fan T., Bennett H., Smith NMixed treatment comparison of infliximab with ustekinumab in patients with moderate to severe psoriasis. British Journal of Dermatology (2011) **165:** (e38-e39).

Gisondi P.,Malara G.,Ardiga M.The psoriatic patient profile for infliximab. European Review for Medical and Pharmacological Sciences (2011) **15:** (1445-1451).

El Maghraoui A.Extra-articular manifestations of ankylosing spondylitis: Prevalence, characteristics and therapeutic implications. European Journal of Internal Medicine (2011) **22:**(554-560).

Afzali A.,Wheat C.,Lee S. The association of psoriasis with anti-tumor necrosis factor (anti-TNF)AQ1 therapy in inflammatory bowel disease: A single-center case series. Inflammatory Bowel Diseases (2011) 17 SUPPL. 2 (S17).

Tejasvi T., Stuart P.E., Chandran V. TNFAIP3 polymorphisms and age at onset are associated with response to tumour necrosis factor blockade in psoriasis. British Journal of Dermatology (2011) **165:(**e9-e10)

Gouldthorpe O.,Catto-Smith A.G.,Alex G.Biologics in paediatric Crohn's disease. Gastroenterology Research and Practice (2011) Article Number: 287574.

Otuki M.F.,Reis R.C.,Cabrini D.Patient-reported outcomes in psoriasis research and practice. British Journal of Dermatology (2011) **165:** (1361-1362).

McGovern D.,Cho J.,Weersma R et al.Shared risk genes between inflammatory bowel disease (IBD) and otherimmune-mediated disorders: First results from the immunochipproject of the international ibd genetics consortium (IIBDGC).Inflammatory Bowel Diseases (2011) 17 SUPPL. 2 (S7-S8).

Yakasai I.A.,Thomson A.J.,Fitzsimons C.Specific dermatoses of pregnancy: A review. West African Journal of Medicine (2011) **30:** (239-244).

Ramos P.S., Criswell L.A., Moser K.L et al. A comprehensive analysis of shared loci between systemic lupus erythematosus (SLE) and sixteen autoimmune diseases reveals limited genetic overlap. PLoS Genetics (2011**) 7:**12 Article Number: e1002406.

Shapiro S., Heremans A., Mays D.A et al. Use of topical tretinoin and the development of noncutaneous adverse events: Evidence from a systematic review of the literature. Journal of the American Academy of Dermatology (2011) **65:** (1194-1201).

Tzellos T., Kyrgidis A., Toulis K. Biologic therapies for chronic plaque psoriasis and cardiovascular events. JAMA - Journal of the American Medical Association (2011) 306:19 (2095).

Ryan C., Daoud Y., Menter A.Biologic therapies for chronic plaque psoriasis and cardiovascular events: In reply. JAMA - Journal of the American Medical Association (2011) **306:** (2095-2096).

Sivakumaran S., Agakov F., Theodoratou E et al. Abundant pleiotropy in human complex diseases and traits. American Journal of Human Genetics (2011) **89:(**607-618).

Micha R., Imamura F., Wyler Von Ballmoos M. Systematic review and meta-analysis of methotrexate use and risk of cardiovascular disease. American Journal of Cardiology (2011) **108:**(1362-1370).

Wu D.,Wu Y.,Liu J.L et al. Association between HLA-Cw*0602 polymorphism and psoriasis risk: A meta-analysis.Genetics and Molecular Research (2011) **10:** (3109-3120).

Huizinga T, Nigrovic P., Ruderman E et al.Association between biologic therapies for chronic plaque psoriasis and cardiovascular events: A meta-analysis of randomized controlled trials – Commentary. International Journal of Advances in Rheumatology (2011) 9:4 (141-142).

Chiu H.-Y.,Tsai T.-F.Topical use of systemic drugs in dermatology: A comprehensive review. Journal of the American Academy of Dermatology (2011) **65:(**1048.e1-1048.e22).

Fan T., Bennett H., Smith N et al. Comparison of infliximab and ustekimumab for treatment of moderate to severe psoriasis: A mixed treatment meta-analysis.Value in Health (2011) 14:7 (A410-A411).

Smoking and psoriasis: A meta-analysis of case-control studies. Zhu K.J.,He S.M.,Sun L.D. et al. Journal of Dermatological Science (2011) **63:**(126-128).

Riveira-Munoz E.,He S.-M.,EscaramÃs G et al Meta-analysis confirms the LCE3C-LCE3B deletion as a risk factor for psoriasis in several ethnic groups and finds interaction with HLA-Cw6.Journal of Investigative Dermatology (2011) 131:5 (1105-1109).

Albrecht L., Bourcier M., Ashkenas J. Topical psoriasis therapy in the age of biologics: Evidence-based treatment recommendations. Journal of Cutaneous Medicine and Surgery (2011) **15:** (309-321).

PÃrez-Alvarez R., DÃaz-Lagares C., GarcÃa-HernÃndez F et al. Hepatitis B virus (HBV) reactivation in patients receiving tumor necrosis factor (TNF)-targeted therapy: Analysis of 257 cases. Medicine (2011) **90:** (359-371).

David C.V., Nguyen H. ,Goldenberg G. Imiquimod: A review of off-label clinical applicationsJ ournal of Drugs in Dermatology (2011) **10:(**1300-1306).

Singal A., Khanna D .Onychomycosis: Diagnosis and management.Indian Journal of Dermatology, Venereology

and Leprology (2011) **77:** (659-672).

Perez-Alvarez R., Perez-de-Lis M., Diaz-Lagares C. Interstitial Lung Disease Induced or Exacerbated by TNF-Targeted Therapies: Analysis of 122 Cases. Seminars in Arthritis and Rheumatism (2011) **41:**(256-264).

Meggitt S.J, Anstey A.V, Mohd Mustapa M.F. British Association of Dermatologists' guidelines for the safe and effective prescribing of azathioprine 2011. British Journal of Dermatology (2011) 165:(711-734).

Muthalaly A., Ang D.C., Hugenberg S.T. Atypical persistent plaquelike skin rash of adult onset still's disease. Case report and review of literature.Arthritis and Rheumatism (2011) 63:10 SUPPL. 1.

Menezes M.D., McCarter R., Greene E.A. Status of propranolol for treatment of infantile hemangioma and description of a randomized clinical trial. Annals of Otology, Rhinology and Laryngology (2011) **120:**(686-695).

Cottone M., Criscuoli V. Infliximab to treat Crohn's disease: An update. Clinical and Experimental Gastroenterology (2011) **4:**(227-238).

Weiss S.T, Silverman E.K. Pro: Genome-wide association studies (GWAS) in asthma. American Journal of Respiratory and Critical Care Medicine (2011) **184:** (631-633).

Reino J.G.,Loza E.,Andreu J.L. et al.Consensus statement of the spanish society of rheumatology on risk management of biologic therapy in rheumatic patients.Reumatologia Clinica (2011) 7:5 (284-298).

Meyersburg D.,RÃ¶del R.M.W.,Meyersburg A.M et al.Nasal septum perforation under long-term therapy with low-dose methotrexate in a patient with psoriasis and psoriatic arthritis.DDG - Journal of the German Society of Dermatology (2011) 9 SUPPL. 5 (13).

Brunasso A.M.G.,Puntoni M.,Gulia A.Safety of anti-tumour necrosis factor agents in patients with chronic hepatitis C infection: A systematic review. Rheumatology (2011) **50:** (1700-1711) Article Number: ker190.

Foulkes A.C.,Grindlay D.J.C.,Griffiths C.E.M et al.What's new in psoriasis? An analysis of guidelines and systematic reviews published in 2009-2010. Clinical and Experimental Dermatology (2011) **36:** (585-588).

Cotsapas C.,Voight B.F.,Rossin E.Pervasive sharing of genetic effects in autoimmune disease. PLoS Genetics (2011) 7:8 Article Number: e1002254.

Fritz H., Kennedy D., Fergusson D. Vitamin A and retinoid derivatives for lung cancer: A systematic review and meta analysis. PLoS ONE (2011) 6:6 Article Number: e21107.

Amitay-Laish I., Stemmer S.M., Lacouture M.E. Adverse cutaneous reactions secondary to tyrosine kinase inhibitors including imatinib mesylate, nilotinib, and dasatinib. Dermatologic Therapy (2011) **24:** (386-395).

Georgiou I. Atlantis mystery and missing heritability. Gastric and Breast Cancer (2011) **10:** (139-145).

Docampo E., Giardina E., Riveira-Muñoz E. Deletion of LCE3C and LCE3B is a susceptibility factor for psoriatic arthritis: A study in Spanish and Italian populations and meta-analysis. Arthritis and Rheumatism (2011) **63: (**1860-1865).

Esparza Gordillo J., Schaarschmidt H., Weidinger S et al. Overlap among inflammatory disorders identifies new genetic risk factors for atopic dermatitisAllergy: European Journal of Allergy and Clinical Immunology (2011) 66 SUPPL. 94 (681).

Actis G.C., Rosina F, MacKay I.R. Inflammatory bowel disease: Beyond the boundaries of the bowel. Expert Review of Gastroenterology and Hepatology (2011) **5:** (401-410).

Kriegel M.A., Manson J.E., Costenbader K.H. Does Vitamin D Affect Risk of Developing Autoimmune Disease?: A Systematic Review.Seminars in Arthritis and Rheumatism (2011) **40:(**512-531.e8).

Smolen J.S., Emery P. Infliximab: 12 years of experience. Arthritis Research and Therapy (2011) 13:SUPPL. 1 Article Number: S2.

Alandete J.C. Effect of treatment switch on the cost-effectiveness of biologics in psoriasis in peru and Colombia. Value in Health (2011) **14:** (A58)

Tan J. Informed shared decision making in psoriasis management.Australasian Journal of Dermatology (2011) 52 SUPPL. 1 (11).

Li W., Han J., Hu F et al. Psoriasis and risk of type 2 diabetes among women and men in the United States. Journal of Investigative Dermatology (2011) 131 SUPPL. 1 (S41).

Schram M.E., Borgonjen R.J., Bik C.M.J.M. Off-label use of azathioprine in dermatology: A systematic review. Archives of Dermatology (2011) **147:** (474-488).

Kurzeja M., Rudnicka L., Olszewska M. New interleukin-23 pathway inhibitors in dermatology: Ustekinumab, briakinumab, and secukinumab. American Journal of Clinical Dermatology (2011) **12:** (113-125).

Capon F. Genetics of psoriasis.Medizinische Genetik (2011) **23:**(67).

Juanola Roura X., Zarco Montejo P., Sanz Sanz J et al. Consensus Statement of the Spanish Society of Rheumatology on the management of biologic therapies in Spondyloarthritis except for Psoriatic Arthritis. Reumatologia Clinica (2011) **7:** (113-123).

Mao Z.-F, Yang L.-X., Mo X.-A et al. Frequency of autoimmune diseases in myasthenia gravis: A systematic review. International Journal of Neuroscience (2011) **121:** (121-129).

Rodgers M., Epstein D., Bojke L et al. Etanercept, infliximab and adalimumab for the treatment of psoriatic arthritis: A systematic review and economic evaluation. Health Technology Assessment (2011) **15:**(134).

Raval K., Lofland J.H., Waters H.C et al. Disease and treatment burden of psoriasis: Examining the impact of biologics. Journal of Drugs in Dermatology (2011) **10:**(189-196).

Domm S., Mrowietz U. Combination therapy in the treatment of psoriasis.JDDG - Journal of the German Society of Dermatology (2011) 9: (94-98).

Dodds M.G., Dong H., Tsuji W et al. Clinical trial simulation to inform phase 2: Comparison of distributed versus concentrated phase 1 study designs. Clinical Pharmacology and Therapeutics (2011) 89 SUPPL. 1 (S84-S85).

Abdullah L., Abbas O. Common nail changes and disorders in older people: Diagnosis and management. Canadian Family Physician (2011) **57:** (173-181).

Byekova Y.A., Hughey L.C., Elewski B.E. Systemic drugs in patients with skin diseases.Giornale Italiano di Dermatologia e Venereologia (2011) **146:** (397-424).

Park H., del Rosso J.Q. The emergence of mycophenolate mofetil in dermatology: From its roots in the world of organ transplantation to its versatile role in the dermatology treatment room.Journal of Clinical and Aesthetic Dermatology (2011) 4:1.

Jensen J.D., Fujita M., Dellavalle R.P. Validation of psoriasis clinical severity and outcome measures: Searching

for a gold standard. Archives of Dermatology (2011) **147:**(95-98).

Van De Kerkhof P.,De Peuter R.,Ryttov J et al.Mixed treatment comparison of a two-compound formulation (TCF) product containing calcipotriol and betamethasone dipropionate with other topical treatments in psoriasis vulgaris.Current Medical Research and Opinion (2011) **27**:(225-238).

Freeman K.The two-compound formulation of calcipotriol and betamethasone dipropionate for treatment of moderately severe body and scalp psoriasis-an introduction. Current Medical Research and Opinion (2011) **27:** (197-203).

Pareek A.,Suthar M.,Rathore G.S et al. Feverfew (Tanacetum parthenium L.): A systematic review. Pharmacognosy Reviews (2011) 5:(103-110).

Marquis P., Caron M., Emery M.-P. The role of health-related quality of life data in the drug approval processes in the us and Europe: A review of guidance documents and authorizations of medicinal products from 2006 to 2010. Pharmaceutical Medicine (2011) **25:**(147-160).

Niazi A.K., Niazi S.K. Mindfulness-based stress reduction: A non-pharmacological approach for chronic illnesses. North American Journal of Medical Sciences (2011) 3: (20-23).

Peyrin-Biroulet L.,Loftus Jr. E.V.,Colombel J.-F et al .Long-term complications, extraintestinal manifestations, and mortality in adult Crohn's disease in population-based cohorts. Inflammatory Bowel Diseases (2011) 17:1 (471-478).

Szepietowski J.,Kaczor M.,Pawlik D et al. Systematic review and mixed treatment comparison of biologic therapies in psoriasis.Dermatologia Kliniczna (2011) 13:(215-228).

Parisi R.,Griffiths C.E.M.,Ashcroft D.M. Systematic review of the incidence and prevalence of psoriasis.British Journal of Dermatology (2011) 165:6 (e5).

Shackleton B.,Bundy C.,Griffiths C. Can stress reduction techniques improve the clinical severity of psoriasis? A systematic review.British Journal of Dermatology (2011) 165:6 (e20-e21).

Baker E.L.,Coleman C.I.,Reinhart K.M et al.Safety of biologic treatments for moderate to severe plaque psoriasis: A systematic review, basic meta-analysis, and Bayesian mixed treatment comparison.Pharmacotherapy (2011) 31:(327e-328e).

Szepietowski J.,Kaczor M.,Pawlik D. et al.Systematic review and mixed treatment comparison of biologic therapies in psoriasis.Journal of Investigative Dermatology (2011) 131 SUPPL. 2 (S38).

Maza A.,MontaudiÃ H.,Sbidian E et al.Oral cyclosporin in psoriasis: A systematic review on treatment modalities, risk of kidney toxicity and evidence for use in non-plaque psoriasis.Journal of the European Academy of Dermatology and Venereology (2011) 25:SUPPL. 2 (19-27).

Paul C.,Ortonne J.-P. Use of conventional systemic treatments in psoriasis: Systematic review and expert opinion of a panel of dermatologists.Journal of the European Academy of Dermatology and Venereology (2011) 25:SUPPL. 2 (1).

Ryan C.,Leonardi C.,Krueger J.,Kimball A.Antagonism of p40 cytokines in psoriasis patients may promote paradoxical cardiovascular risk.Journal of Investigative Dermatology (2011) 131 SUPPL. 1 (S83).

Dommasch E.,Troxel A.,Shin D et al.The safety of tumor necrosis factor antagonists in patients with psoriatic disease: A systematic review and metaanalysis of randomized controlled trials.Journal of the American Academy of Dermatology (2011) 64:2 SUPPL. 1 (AB8).

2010

RodrÃguez Lozano C.Safety of biological therapies: New data from BIOBADASER. Reumatologia Clinica (2010) 6:SUPPL. 3 (S1-S6).

Price S.,James C.,Deighton C.Methotrexate use and alcohol. Clinical and Experimental Rheumatology (2010) **28:** SUPPL. 61 (S114-S116).

Alandete J.C. Cost-effectiveness of biologics in psoriasis in two la countries-comparison with the European experience. Value in Health (2010) **13:** (A467-A468).

Ioannidis J.,Karassa F.The need to consider the wider agenda in systematic reviews and meta-analyses. BMJ (Online) (2010) 341:7776 (762-765) Article Number: c4875.

Schram M.E.,Spuls P.I.,Bos J.D.Off-label use of efalizumab in dermatology. Expert Review of Dermatology (2010) **5:** (535-547).

Papp K.Clinical development of onercept, a tumor necrosis factor binding protein, in psoriasis. Current Medical Research and Opinion (2010) **26:** (2287-2300).

Finckh A.Comparative effectiveness of rheumatoid arthritis therapies. Current Rheumatology Reports (2010) **12:** (348-354).

Cantini F.,Niccoli L.,Nannini C.Psoriatic arthritis: A systematic review. International Journal of Rheumatic Diseases (2010) **13:**(300-317).

Signorovitch J.E.,Wu E.Q.,Yu A.P et al.Comparative effectiveness without head-to-head trials: A method for matching-adjusted indirect comparisons applied to psoriasis treatment with adalimumab. PharmacoEconomics (2010) **28:**(935-945).

Garg A.D.,Nowis D.,Golab J.Photodynamic therapy: Illuminating the road from cell death towards anti-tumour immunity. Apoptosis (2010) **15:**(1050-1071).

Yang S.,Liu J.,Zhang C et al. Meta-GWAS of psoriasis reveals genetic heterogeneity between Chinese and European populations and identifies two new susceptibility loci. Journal of Dermatology (2010) 37 SUPPL. S1 (102).

Herfarth H.H., Osterman M.T., Isaacs K.L et al. Efficacy of methotrexate in ulcerative colitis: Failure or promise.Inflammatory Bowel Diseases (2010) **16:(**1421-1430).

Malhi G.S., Adams D., Berk M. The pharmacological treatment of bipolar disorder in primary care. Medical Journal of Australia (2010) **193:**SUPPL. 4 (S24-S30).

Pietrzik K., Bailey L., Shane B. Folic acid and l-5-methyltetrahydrofolate: Comparison of clinical pharmacokinetics and pharmacodynamics. Clinical Pharmacokinetics (2010) **49:(**535-548).

Garcia-Bustinduy M.C., Gonzalez-Hernandez S., Garcia C.R et al. Cutaneous reactions in biological treatments. Journal of the European Academy of Dermatology and Venereology (2010) 24 SUPPL. 4 (27-28).

Stuart P.E., Nair R.P., ELinghaus E. et al. Genome-wide asociation analysis identifies three psoriasis

susceptibility loci. Nature Genetics (2010) **42:** (1000-1004).

Piruzian E., Bruskin S., Ishkin A. et al. Integrated network analysis of transcriptomic and proteomic data in psoriasis. BMC systems biology (2010) 4 (41).

Kwok T.,Loo W.J.,Guenther L. Psoriasis and multiple sclerosis: Is there a link? Journal of Cutaneous Medicine and Surgery (2010) **14:** (151-155).

Tatar M.,Sarioz F.Cost efficacy of ustekinumab in treatment of moderate to severe plaque psoriasis in Turkey. Value in Health (2010) **13:** (A146-A147).

Rioux J.D. International inflammatory bowel disease genetics consortium identifies >50 genetic risk factors for ulcerative colitis. Gastroenterology (2010) 139:(e19).

Mars T.S.,Abbey H.Mindfulness meditation practise as a healthcare intervention: A systematic review. International Journal of Osteopathic Medicine (2010) 13:2 (56-66).

Ormerod A.D.,Campalani E.,Goodfield M.J.D.British Association of Dermatologists guidelines on the efficacy and use of acitretin in dermatology. British Journal of Dermatology (2010) 162:5 (952-963).

DÃaz-Lagares C.,Belenguer R.,Ramos-Casals M.Systematic review on the use of adalimumab in autoinmune. Efficacy and safety in 54 patients. Reumatologia Clinica (2010) **6:** (121-127).

Lazaro P.,Blasco A.J.,Ferrandiz C et al .Efficiency (cost/efficacy) of biologic agents in the treatment of moderate to severe psoriasis. Value in Health (2010) **13:** (A147).

Langham S.,Langham J.,Goertz H.PLarge-scale, prospective, observational studies in patients with psoriasis and psoriatic arthritis: A systematic and critical review. Value in Health (2010) **13:**(A217).

Vyas R.,Juruwan R.,Rademaker M.Use of transient elastography for non-invasive monitoring of methotrexate induced liver fibrosis. Australasian Journal of Dermatology (2010) 51 SUPPL. 1 (A46-A47).

Pedraz J., Dauden E. Psoriatic arthritis and etanercept. Actas Dermo-Sifiliograficas (2010) 101:SUPPL. 1 (26-34).

Ramos P.S., Gaffney P.M., Criswell L.A. Meta-analysis of autoimmune variants shared between systemic lupus erythematosus (SLE) and 16 other diseases identifies novel SLE loci. Arthritis and Rheumatism (2010) 62 SUPPL. 10 (500).

Gaujoux-Viala C., Giampietro C., Gaujoux T et al. Scleritis: A new paradoxical effect of etanercept? A series of 3 cases and a systematic literature review of etanercept-associated inflammatory eye disease. Arthritis and Rheumatism (2010) 62 SUPPL. 10 (413).

Kirino Y., Ombrello M.J., Gul A. Homozygous 3.2kb deletion in LEPREL1 (P3H2) intron 1 reduces cytokine production and protects from multiple inflammatory diseases. Arthritis and Rheumatism (2010) 62 SUPPL. 10 (2217).

Bailey J., Whitehair B. Topical treatments for chronic plaque psoriasis. American Family Physician (2010) **81: (**596-597).

Powers J., Martin R. Incidence of serious infectious events with methotrexate treatment: Metaanalysis of randomized controlled trials. Journal of the American Academy of Dermatology (2010) 62:3 SUPPL. 1 (AB4).

Raap U., Staonder S. Pruritus and skin: New facts on pruritus induction. Allergo Journal (2010) **19:**(58-65)

Jensen P.,Skov L.,Zachariae C. Systemic combination treatment for psoriasis: A review. Acta Dermato-Venereologica (2010) **90:**(341-349).

Beani J.-C.,Jeanmougin M. Narrow-band UVB therapy in psoriasis vulgaris: Good practice guideline and recommendations of the French Society of Photodermatology. Annales de Dermatologie et de Venereologie (2010) **137:**(21-31).

Nybaek H.,Jemec G.B.E.Skin problems in stoma patients. Journal of the European Academy of Dermatology and Venereology (2010) **24:**(249-257).

Schafer H.,Simonsen L.Fusidic acid in dermatology: An updated review. European Journal of Dermatology (2010) **20:**(6-15).

Shale M.J.,Seow C.H.,Coffin C.Set al.Review article: Chronic viral infection in the anti-tumour necrosis factor therapy era in inflammatory bowel disease. Alimentary Pharmacology and Therapeutics (2010) **31:**(20-34).

Naci H.,Green J.,Prasad M et al.Biological agents for the treatment of nail psoriasis: A systematic review of the literature.Value in Health (2010) 13:7 (A460).

Wiwanitkit V.LCE3C_LCE3B-del genotype and psoriasis: A summative meta-analysis.Acta Dermatovenerologica Croatica (2010) **18:(**130).

Sun Y.,Wu Y.,Chen L.-H et al.Efficacy and safety of adalimumab for plaque psoriasis: A systematic review.Chinese Journal of Evidence-Based Medicine (2010) **10:(**1085-1095).

Espinoza-Chavez V.E.Meta-analysis**:** Psoriasis and BMI.Journal of the European Academy of Dermatology and Venereology (2010) 24 SUPPL. 4 (54).

Dommasch E.D.,Abuabara K.,Nguyen J.The safety of tumor necrosis factor antagonists in psoriasis: A systematic review and meta-analysis of randomized controlled trials.Journal of Investigative Dermatology (2010) 130 SUPPL. 1 (S59).

Paul C.,Ortonne J.-P.Introduction: Psoriasis evaluation in clinical practice: Systematic review and expert opinion.Journal of the European Academy of Dermatology and Venereology (2010) 24:SUPPL. 2 (1).

Storage S.S.,Agrawal H.,Furst D.E.Description of the efficacy and safety of three new biologics in the treatment of rheumatoid arthritis.Korean Journal of Internal Medicine (2010) **25:**(1-17).

Mrowietz U.,GuÃrin A.,Mulani P et al.Comorbidity prevalence in psoriasis patients: A meta-analysis.Journal of the American Academy of Dermatology (2010) 62:3 SUPPL. 1 (AB123).

Feldman S.,Yentzer B.Topical clobetasol propionate in the treatment of psoriasis.Journal of the American Academy of Dermatology (2010) 62:3 SUPPL. 1 (AB140).

Menon K.,Van Voorhees A.S.,Bebo Jr. B.F et al.Psoriasis in patients with HIV infection: From the Medical Board of the National Psoriasis Foundation.Journal of the American Academy of Dermatology (2010) **62:(**291-299).

2009

Poulin Y.,Langley R.,Teixeira H.D et al.Biologics in the treatment of psoriasis: Clinical and economic overview. Journal of Cutaneous Medicine and Surgery (2009) 13:SUPPL. 2 (S49-S57).

Casanova J.M.,SanmartÃn V.,Soria X et al. Topical treatment of psoriasis. Piel (2009) 24:10 (556-567).

Haslund P., Lee R.A., Jemec G.B.E. Treatment of hidradenitis suppurativa with tumour necrosis factor-Î± inhibitors. Acta Dermato-Venereologica (2009) **89:**(595-600).

Schuh A. Evidence of the efficacy of climatotherapy and thalassotherapy - A review. Schweizerische Zeitschrift fur GanzheitsMedizin (2009) **21:** (96-104).

Godse K.V. Chronic urticaria and treatment options. Indian Journal of Dermatology (2009) **54:** (310-312).

Cantini F., Nannini C., Niccoli L. Bioboosters in the treatment of rheumatic diseases: A comprehensive review of currently available biologics in patients with rheumatoid arthritis, ankylosing spondylitis and psoriatic arthritis. Open Access Rheumatology: Research and Reviews (2009) **1:**(163-178).

Atzeni F., Sarzi-Puttini P. Anti-cytokine antibodies for rheumatic diseases. Current Opinion in Investigational Drugs (2009) **10:**(1204-1211).

Blasco A.J., Lazaro P., Ferrandiz C et al. Efficiency of biologic agents in the treatment of moderate to severe psoriasis. Actas Dermo-Sifiliograficas (2009) **100:**(792-803).

Ma C., Panaccione R., Heitman S.J. Systematic review: The short-term and long-term efficacy of adalimumab following discontinuation of infliximab. Alimentary Pharmacology and Therapeutics (2009) 30:10 (977-986).

Yu X., Wieczorek S., Franke A et al. Association of UCP2 -866 G/A polymorphism with chronic inflammatory diseases.Genes and Immunity (2009) 10:6 (601-605).

Dommasch E.,Gelfand J.MIs there truly a risk of lymphoma from biologic therapies?.Dermatologic Therapy (2009) **22:** (418-430).

Ritchlin C.T.,Kavanaugh A.,Gladman D.D. et al. Treatment recommendations for psoriatic arthritis.Annals of the Rheumatic Diseases (2009) **68:** (1387-1394).

Turner D.,Picot J.,Cooper K et al.Adalimumab for the treatment of psoriasis. Health technology assessment (Winchester, England) (2009) 13 Suppl 2 (49-54).

Braun J.,Baraliakos X.Treatment of ankylosing spondylitis and other spondyloarthritides. Current Opinion in Rheumatology (2009) **21:** (324-334).

Chiang N.Y.Z., Panting K.,Parslew R.A.G.Clinical applicability of T-SPOT. TB screening prior to initiation of antitumour necrosis factor-Î± therapy. British Journal of Dermatology (2009) 161 SUPPL. 1 (43).

Bray A., Barnova I., Przemioslo R et al. Could transient elastography reduce the need for Liver biopsy?. British Journal of Dermatology (2009) 161 SUPPL. 1 (10-11).

Andronis L., Barton P., Bryan S. Sensitivity analysis in economic evaluation: An audit of NICE current practice and a review of its use and value in decision-making. Health Technology Assessment (2009) 13: (ix-84).

Zivkovich A.H., Feldman S.R. Are ointments better than other vehicles for corticosteroid treatment of psoriasis?. Journal of Drugs in Dermatology (2009) **8:**(570-572

Sizto S., Bansback N., Feldman S.R. Economic evaluation of systemic therapies for moderate to severe

psoriasis. British Journal of Dermatology (2009) **160:** (1264-1272).Abstracts Presented at the 12th Joint Meeting of the International Society of Dermatopathology. American Journal of Dermatopathology (2009) 31:4.

Qureshi S.U.,Pyne J.M.,Magruder K.M et al. The link between post-traumatic stress disorder and physical comorbidities: A systematic review. Psychiatric Quarterly (2009) 80: (87-97)

Karaderi T., Harvey D., Farrar C et al. Association between the interleukin 23 receptor and ankylosing spondylitis is confirmed by a new UK case-control study and meta-analysis of published series.Rheumatology (2009) 48:4 (386-389).

Ko J.M., Gottlieb A.B., Kerbleski J.F. Induction and exacerbation of psoriasis with TNF-blockade therapy: A review and analysis of 127 cases.Journal of Dermatological Treatment (2009) 20:2 (100-108).

E.,Raychaudhuri S.,Chen R.Meta-analysis of six genome-wide association studies in >25,000 case-control samples identifies new rheumatoid arthritis risk loci. Arthritis and Rheumatism (2009) 60 SUPPL. 10 (1894).

Schmajuk G, Krishnan E. Real or perceived conflicts of interest in comparative effectiveness research. Arthritis and Rheumatism (2009) 60 SUPPL. 10 (1015).

Diener H.C, Hartung H.P., Bien C.G. et al. Clinical trials in neurology in Germany 2008. Aktuelle Neurologie (2009) **36:**(8-18).

Souza R.C., Lima H.C., Bottmann P. Cost analysis of system therapy for psoriasis in Brazil. Journal of the American Academy of Dermatology (2009) 60:3 SUPPL. 1 (AB166).

Albrecht J., Werth V.P, Bigby M. The role of case reports in evidence-based practice, with suggestions for improving their reporting. Journal of the American Academy of Dermatology (2009) **60:**(412-418).

Forabosco P., Bouzigon E., Ng M.Y. Meta-analysis of genome-wide linkage studies across autoimmune diseases. European Journal of Human Genetics (2009) **17:**(236-243).

Zhang Z., Schmitt J., Wozel G et al. Treatment of plaque psoriasis with biologics. A meta-analysis of randomized controlled trial. Medizinische Klinik (2009) **104**:(125-136).

Olivieri I., D'Angelo S., Palazzi C. Treatment strategies for early psoriatic arthritis. Expert Opinion on Pharmacotherapy (2009) **10:** (271-282).

Lima X.T., Seidler E.M., Lima H.C et al.Long-term safety of biologics in dermatology. Dermatologic Therapy (2009) **22:**(2-21).

Gladman D.D.Psoriatic arthritis. Dermatologic Therapy (2009) **22:** (40-55).

Falagas M.E., Zarkadoulia E., Rafailidis P.I. The therapeutic effect of balneotherapy: Evaluation of the evidence from randomised controlled trials. International Journal of Clinical Practice (2009) **63:** (1068-1084).

Feily A., Namazi M.R. Aloe vera in dermatology: A brief review. Giornale Italiano di Dermatologia e Venereologia (2009) **144:**(85-91).

Frankel A.J.,Van Voorhees A.S.,Hsu S.et al Treatment of psoriasis in patients with hepatitis C: From the Medical Board of the National Psoriasis Foundation.Journal of the American Academy of Dermatology (2009) **61:** (1044-1055).

Kircik L.H.,Weinberg J.M.Critical reviews of clinical data: Focus on T cell agents for the treatment of

psoriasis.Journal of Drugs in Dermatology (2009) **8:** (6-15).

Maringer B.,Zietemann V.,Ratzinger G. et al.Effectiveness of omega-3-fatty acids in psoriasis: A systematic review.Aktuelle Ernahrungsmedizin (2009) **34:**(195-200).

Brown B.C.,Warren R.B.,Grindlay D.J.C et al.What's new in psoriasis? Analysis of the clinical significance of systematic reviews on psoriasis published in 2007 and 2008.Clinical and Experimental Dermatology (2009) 34:6 (664-667).

Hawkins N.S., Huntley A., Eaton J. **Meta-analysis o**f biologic therapies for the treatment of moderate to severe psoriasis.Value in Health (2009) 12:(A74-A75).

Levy-Roy A.,Porcher R.,De Fonclare A.-L et al.EfficacitÃ des anti-TNF-Î± dans le psoriasis: Revue systÃmatique et reprentation graphique.Annales de Dermatologie et de Venereologie (2009) 136: (315-322).

De Peuter R.,Beriot-Mathiot A.,Eijgelshoven I et al.Efficacy of psoriasis treatments: Systematic literature review and Bayesian metaanalysis.Journal of the American Academy of Dermatology (2009) 60:3 SUPPL. 1 (AB177).

2008

Morton C.A.,McKenna K.E.,Rhodes L.E.Guidelines for topical photodynamic therapy: Update. British Journal of Dermatology (2008) **159:**(1245-1266).

Ortonne J.-P.What's new in dermatological treatments? Annales de Dermatologie et de Venereologie (2008) 135:SUPPL. 7 (S360-S370).

Li Y.,Chang M., Schrodi S.J et al. The 5q31 variants associated with psoriasis and Crohn's disease are distinct. Human Molecular Genetics (2008) 17:19 (2978-2985).

Davis Jr. J.C., Mease P.J.Insights Into the Pathology and Treatment of Spondyloarthritis: From the Bench to the Clinic. Seminars in Arthritis and Rheumatism (2008) **38:**(83-100).

Buell C.,Koo J. Long-term safety of mycophenolate mofetil and cyclosporine: a review. Journal of drugs in dermatology : JDD (2008) **7:**(741-748).

Alamanos Y., Voulgari P.V., Drosos A.A. Incidence and prevalence of psoriatic arthritis: A systematic review. Journal of Rheumatology (2008) **35:** (1354-1358).

Stewart L.A., Rydzewska L.H.M., Keogh G.F et al. Systematic review of therapeutic interventions in human prion disease. Neurology (2008) **70:** (1272-1281).

Thiers B.H.What's new in dermatologic therapy. Dermatologic Therapy (2008) **21**:(142-149).

Rich P., Griffiths C.E.M., Reich K. Baseline nail disease in patients with moderate to severe psoriasis and response to treatment with infliximab during 1 year. Journal of the American Academy of Dermatology (2008) **58**:(224-231).

Haider A.S., Lowes M.A., Suarez-Fariñas M. Identification of cellular pathways of type 1, Th17 T cells, and TNF- and inducible nitric oxide synthase-producing dendritic cells in autoimmune inflammation through pharmacogenomic study of cyclosporine A in psoriasis. Journal of Immunology (2008) **180:** (1913-1920).

Falagas M.E., Vouloumanou E.K.,Matthaiou D.K. Effectiveness and safety of short-course vs long-course

antibiotic therapy for group A Î²-hemolytic streptococcal tonsillopharyngitis: A meta-analysis of randomized trials. Mayo Clinic Proceedings (2008) **83:** (880-889).

Reich K.,Sinclair R.,Roberts G.Comparative effects of biological therapies on the severity of skin symptoms and health-related quality of life in patients with plaque-type psoriasis: A meta-analysis.Current Medical Research and Opinion (2008) **24:**(1237-1254).

2007

Ulbricht C., Armstrong J., Basch E. Journal of Herbal Pharmacotherapy (2007) 7:3-4 (279-323).

Furst D.E., Breedveld F.C., Kalden J.R et al.Updated consensus statement on biological agents for the treatment of rheumatic diseases, 2007. Annals of the Rheumatic Diseases (2007) 66:SUPPL. 3 (iii2-iii22).

Reich A.,Szepietowski J.Etanercept significantly improves quality of life of patients with psoriasis: Current literature review.

Diamond G.A.,Bax L.,Kaul S.Uncertain effects of rosiglitazone on the risk for myocardial infarction and cardiovascular death.Annals of Internal Medicine (2007) **147:** (578-581).

Mariette X.Anticytokines (anti-TNF) and infection risk.Revue du Praticien (2007) **57:(**1681-1682).

Steptoe A.,Hamer M.,Chida Y.The effects of acute psychological stress on circulating inflammatory factors in humans: A review and meta-analysis.Brain, Behavior, and Immunity (2007) **21:**(901-912)

Eggebeen A.T.Gout: An update.American Family Physician (2007)76:(801-808+811-812).

Bessell A.,Moss T.P. Evaluating the effectiveness of psychosocial interventions for individuals with visible differences: A systematic review of the empirical literature.
Body Image (2007) **4:**(227-238).

Canter P.H.,Wider B.,Ernst E.The antioxidant vitamins A, C, E and selenium in the treatment of arthritis: A systematic review of randomized clinical trials.Rheumatology (2007) **46:**(1223-1233).

Tilg H.,Feichtenschlager T.,Knoflach P.Use of infliximab in ulcerative colitis.Zeitschrift fur Gastroenterologie (2007) **45:**(907-911).

Nast A.,Kopp I.B.,Augustin M.Evidence-based (S3) guidelines for the treatment of psoriasis vulgaris.JDDG - Journal of the German Society of Dermatology (2007) 5:SUPPL 3 (1-119).

Vena G.A.,Cassano N.Anti-tumor necrosis factor therapies for psoriasis.Expert Review of Dermatology (2007) 2:3 (335-349).

Ruxton C.H.S.,Reed S.C.,Simpson J.A et al.The health benefits of omega-3 polyunsaturated fatty acids: A review of the evidence.Journal of Human Nutrition and Dietetics (2007) **20:** (275-285).

Chan K.H.N.Optimising the management of psoriasis with biologics. Hong Kong Journal of Dermatology and Venereology (2007) **15:**(88).

Lichtenstein G.R.General safety concerns associated with biologic therapies.Gastroenterology and Hepatology (2007) **3:**(4-6).

D'Haens G.Risk and benefits of biologic therapy for inflammatory bowel diseases.Gut (2007) 56:5 (725-732).

Manriquez J.J,Villouta M.F.,Williams H.C.Evidence-based dermatology: Number needed to treat and its relation to other risk measures.Journal of the American Academy of Dermatology (2007) 56:4 (664-671).

Thompson Coon J.,Pittler M.H.,Ernst E.Trifolium pratense isoflavones in the treatment of menopausal hot flushes: A systematic review and meta-analysis.Phytomedicine (2007) **14:**(153-159).

Khan S., Justice E., Jobanputra P. Adverse reactions and safety of newer disease-modifying antirheumatic drugs (DMARDs) for rheumatoid arthritis.Adverse Drug Reaction Bulletin (2007) :**242** (927-930).

De Argila D., RodrÃguez-Nevado I., Chaves A. Cost-effectiveness analysis comparing methotrexate with puva therapy for moderate-severe psoriasis in the sanitary area of Badajoz. Actas Dermo-Sifiliograficas (2007) **98**:1 (35-41).

Xu L.-M.,Ru X.-Y.,Wu T.-X.Meta-analysis of the relationship between IFN-Î³ and psoriasis.Chinese Journal of Evidence-Based Medicine (2007) 7:(516-522).

Leon A.,Nguyen A.,Letsinger J et al.An attempt to formulate an evidence-based strategy in the management of moderate-to-severe psoriasis: A review of the efficacy and safety of biologics and prebiologic options.Expert Opinion on Pharmacotherapy (2007) 8:(617-632).

Philipp S.,Morcinietz C.S.,Wallace L.,Sterry W.   Biologics for the treatment of psoriasis: A systematic review.Giornale Italiano di Dermatologia e Venereologia (2007) 142: (567-591).

Li C.,Wang G.,Gao Y et al.TNF-Î± gene promoter -238G>A and -308G>A polymorphisms alter risk of psoriasis vulgaris: A meta-analysis. Journal of Investigative Dermatology (2007) **127:** (1886-1892).

Yan K.-L.,Huang W.,Zhang X.-J et al.Follow-up analysis of PSORS9 in 151 Chinese families confirmed the linkage to 4q31-32 and refined the evidence to the families of early-onset psoriasis. Journal of Investigative Dermatology (2007) **127:**(312-318).

Hankin C.S.,Feldman S.R,Szczotka A et al. A cost comparison of treatments of moderate to severe psoriasis. Drug Benefit Trends (2005) **17**: (200-214).

2006

Wolkenstein P.Living with psoriasis. Journal of the European Academy of Dermatology and Venereology (2006) 20:SUPPL. 2 (28-32).

Stern R.S.Lymphoma risk in psoriasis: Results of the PUVA follow-up study. Archives of Dermatology (2006) **142:**(1132-1135).

Chen S.-L.,Yan J.,Liu Y et al.Efalizumab for psoriasis: A systematic review. Chinese Journal of Evidence-Based Medicine (2006) 6:27-272.

Aziz R., Lorberg B., Tampi R.R. Treatments for late-life bipolar disorder. American Journal Geriatric Pharmacotherapy (2006) 4:4 (347-364).

Haase I.Response to Clark and Kupper Journal of Clinical Investigation (2006)116:(3088).

Ritchlin C.,Tausk F.A medical conundrum: Onset of psoriasis in patients receiving anti-tumour necrosis factor agents.Annals of the Rheumatic Diseases (2006)**65:**(1541-1544).

Kerns M.Jo.J.,Graves J.E.,Smith D.I.Off-Label Uses of Biologic Agents in Dermatology: A 2006 Update.Seminars in Cutaneous Medicine and Surgery (2006) **25:(**226-240).

Aubin F.,Laurent R.Human papillomavirus-associated cutaneous lesions.Revue du Praticien (2006) **56:**(1905-1911).

Tilg H.,Kaser A.,Moschen A.R.How to modulate inflammatory cytokines in liver diseases.Liver International (2006) **26:**(1029-1039).

Bayos M.,Rabasseda X.,Prous J.R.Gateways to clinical trials: November 2006.Methods and Findings in Experimental and Clinical Pharmacology (2006) 28:9 (657-678).

Furst D.E.,Breedveld F.C.,Kalden J.R .Updated consensus statement on biological agents for the treatment of rheumatic diseases, 2006.Annals of the Rheumatic Diseases (2006) 65:SUPPL. 3 (iii2-iii15).

Wollina U.,Hansel G., Koch A Topical pimecrolimus for skin disease other than atopic dermatitis.Expert Opinion on Pharmacotherapy (2006) **7:**(1967-1975).

Staunton D.E,Lupher M.L.,Liddington R et al Targeting Integrin Structure and Function in Disease.Advances in Immunology (2006) 91 (111-157).

Ruderman E.Adalimumab in psoriatic arthritis: A viewpoint by Eric Ruderma.Drugs (2006) **66:** (1497).

Shigaki C.L.,Glass B.,Schopp L.H.Mindfulness-based stress reduction in medical settings. Journal of Clinical Psychology in Medical Settings (2006) 13:3 (209-216).

Woolacott N.,Vergel Y.B.,Hawkins N et al. Etanercept and infliximab for the treatment of psoriatic arthritis: A systematic review and economic evaluation.Health Technology Assessment (2006) **10:**(iii-78).

Woolacott N.F.,Khadjesari Z.C.S.,Bruce I.N et al Etanercept and infliximab for the treatment of psoriatic arthritis: A systematic review .Clinical and Experimental Rheumatology (2006) **24:** (587-593).

A betamethasone-calcipotriene combination for psoriasis.Medical Letter on Drugs and Therapeutics (2006) **48:** (55-56).

Kavanaugh A.F.,Ritchlin C.T.,Boehncke W.-H.Systematic review of treatments for psoriatic arthritis: An evidence based approach and basis for treatment guidelinesJournal of Rheumatology (2006) **33:** (1417-1421).

Helliwell P.S.Therapies for dactylitis in psoriatic arthritis. A systematic review. Journal of Rheumatology (2006) **33:** (1439-1441).

Nash P.Therapies for axial disease in psoriatic arthritis. A systematic review.Journal of Rheumatology (2006) 33: (1431-1434).

Soriano E.R., McHugh N.J. Therapies for peripheral joint disease in psoriatic arthritis. A systematic review. Journal of Rheumatology (2006) **33:** (1422-1430).

Adams A.K.,Warshaw E.M.Allergic contact dermatitis from mercapto compounds. Dermatitis (2006) **17:** (56-70).

Kemany L.,Brodszky V,KÃrpÃti K et al.The role of biological drugs in the treatment of psoriasis, results from 9

randomized placebo-controlled trials. Orvosi Hetilap (2006) **147:(**981-990).

Lim L., Suhler E.B., Smith J.R. Biologic therapies for inflammatory eye disease. Clinical and Experimental Ophthalmology (2006) **34:** (365-374).

Carey W.,Glazer S.,Gottlieb A.B et al. Relapse, rebound, and psoriasis adverse events: An advisory group report. Journal of the American Academy of Dermatology (2006) 54:4 SUPPL. (S171-S181).

Menter A.,Leonardi C.L.,Sterry W et al. Long-term management of plaque psoriasis with continuous efalizumab therapy. Journal of the American Academy of Dermatology (2006) 54:4 SUPPL. (S182-S188).

Shear N.H.Fulfilling an unmet need in psoriasis: Do biologicals hold the key to improved tolerability?.Drug Safety (2006) **29:** (49-66).

Young A.H.,Newham J.I.Lithium in maintenance therapy for bipolar disorder.Journal of Psychopharmacology (2006) **20:** SUPPL. (17-22).

Bayas M.,Rabasseda X.,Prous J.R.Gateways to Clinical Trials.Methods and Findings in Experimental and Clinical Pharmacology (2006) **28:**(31-63).

Hawkins T.Appearance-related side effects of HIV-1 treatment.AIDS Patient Care and STDs (2006) **20:** (6-18).

Hankey G.J.Is plasma homocysteine a modifiable risk factor for stroke?.Nature Clinical Practice Neurology (2006) **2:** (26-33).

2005

Fisher V.S.Clinical monograph for drug formulary review: systemic agents for psoriasis/psoriatic arthritis. Journal of managed care pharmacy : JMCP (2005) **11:**(33-55).

Ulbricht C.,Basch E.,Barrette E.-P et al.Shark cartilage: An evidence-based systematic review for the natural standard research collaboration. Journal of Cancer Integrative Medicine (2005) **3:** (99-111).

Zhou X.,Schmidtke P.,Zepp F et al.Boosting interleukin-10 production: Therapeutic effects and mechanisms. Current Drug Targets: Immune, Endocrine and Metabolic Disorders (2005) **5:** (465-475).

Mariwalla K.,Rohrer T.E. Use of lasers and light-based therapies for treatment of acne vulgaris. Lasers in Surgery and Medicine (2005) **37:** (333-342).

Gordon K.B.,Bonish B.K.,Patel T et al.The tumour necrosis factor-Î± inhibitor adalimumab rapidly reverses the decrease in epidermal Langerhans cell density in psoriatic plaques.British Journal of Dermatology (2005) **153:** (945-953).

Parchamazad P.,Ghazvini P.,Honeywell M et al.Abatacept (CTLA4-Ig, Orencia): An investigational biological compound for the treatment of rheumatoid arthritis.P and T (2005) 30:11 (633-638+643+669).

Cianci R.,Cammarota G.,Raducci F et al.The impact of biological agents interfering with receptor/ligand binding in the immune system.European Review for Medical and Pharmacological Sciences (2005) 9:6 (305-314).

LodÃn M.The clinical benefit of moisturizers .Journal of the European Academy of Dermatology and Venereology (2005) 19:6 (672-688).

Ponticelli C.Cyclosporine: From renal transplantation to autoimmune diseases.Annals of the New York Academy of Sciences (2005) 1051 (551-558).

Herbal medicine.Focus on Alternative and Complementary Therapies (2005) 10:3 (222-241).

Dagenais N.J.,Jamali F.Protective effects of angiotensin II interruption: Evidence for antiinflammatory actions.Pharmacotherapy (2005) **25:** (1213-1229).

Follmann M.,Sterry W.,Rzany B.Development of the evidence-based guidelines for psoriasis - A project of the German dermatological society (DDG).JDDG - Journal of the German Society of Dermatology (2005) **3:** (678-689).

Harries M.J.,Chalmers R.J.G.,Griffiths C.E.M.Fumaric acid esters for severe psoriasis: A retrospective review of 58 cases.British Journal of Dermatology (2005) **153:** (549-551).

Van De Kerkhof P.C.M.,Wasel N.,Kragballe K et al.A two-compound product containing calcipotriol and betamethasone dipropionate provides rapid, effective treatment of psoriasis vulgaris regardless of baseline disease severity.Dermatology (2005) **210:**(294-299).

Lee C.S.,Koo J.A review of acitretin, a systemic retinoid for the treatment of psoriasis.Expert Opinion on Pharmacotherapy (2005) **6:** (1725-1734).

Malhotra S.,Bansal D.,Shafiq N.Potential therapeutic role of peroxisome proliferator activated receptor-Î³ agonists in psoriasis.Expert Opinion on Pharmacotherapy (2005) **6:** (1455-1461).

Mealy N.E.,BayÃs M.Infliximab.Drugs of the Future (2005) 30:8 (845-846).

Fischer U.,Schulze-Osthoff K.Pharmacological modulation of caspase activation.
Current Medicinal Chemistry: Anti-Inflammatory and Anti-Allergy Agents (2005) 4:4 (407-419).

Dominguez C.,Tamayo N.,Zhang D.p38 inhibitors: Beyond pyridinylimidazoles"""
Expert Opinion on Therapeutic Patents (2005) **15:**(801-816).

Nasermoaddeli A.,Kagamimori S.Balneotherapy in medicine: A review.Environmental Health and Preventive Medicine (2005) **10:**(171-179).

Huntley A.L.,Coon J.T.,Ernst E.The safety of herbal medicinal products derived from Echinacea species: A systematic review.Drug Safety (2005) **28:**(387-400).

Kostovic K.,Pasic A.New treatment modalities for vitiligo: Focus on topical immunomodulators.Drugs (2005) **65:** (447-459).

Davidson A.,Diamond B.,Wofsy D et al. Block and tackle: CTLA4Ig takes on lupus.Lupus (2005) **14:** (197-203).

SchÃn M.P.,Ludwig R.J. Lymphocyte trafficking to inflamed skin - Molecular mechanisms and implications for therapeutic target molecules.Expert Opinion on Therapeutic Targets (2005) **9:** (225-243).

Arnaud C.,Veillard N.R.,Mach F.Cholesterol-independent effects of statins in inflammation, immunomodulation and atherosclerosis.Current Drug Targets - Cardiovascular and Haematological Disorders (2005) **5:**(127-134).

Braddock M.Anti-arthritis therapies 2005. 23-24 February 2005, London, UK.IDrugs (2005) **8:** (288-289).

Labro M.-T.Biotechnology - Fourth Croatian International Scientific Conference. Biotechnology and immunomodulatory drugs. 20-23 February 2005, Zagreb, Croatia.

IDrugs (2005) **8:**(290-293).

Wijdenes J.The new wave in mAbs therapeutics: Putting high expectations in perspective.EBR - European Biopharmaceutical Review (2005) :SPRING (50-55).

Buka R.L.,Cunningham B.B.Connective tissue disease in children.Pediatric Annals (2005) **34:** (225-238).

Hrastinger A.,Dietz B.,Bauer R et al.Is there clinical evidence supporting the use of botanical dietary supplements in children?.Journal of Pediatrics (2005) **146:** (311-317).

Strieter R.M.,Belperio J.A.,Burdick M.D et al CXC chemokines in angiogenesis relevant to chronic fibroproliferation.Current Drug Targets: Inflammation and Allergy (2005) **4:** (23-26).

Eklund J.W.,Kuzel T.M.Denileukin diftitox: A concise clinical review.Expert Review of Anticancer Therapy (2005) **5:** (33-38).

Ranieri G.,Ria R.,Roccaro A.M. et al.Development of vasculature targeting strategies for the treatment of chronic inflammatory diseases.Current Drug Targets: Inflammation and Allergy (2005) **4:**(13-22).

Jais P.H.How frequent is altered gene expression among susceptibility genes to human complex disorders?.Genetics in Medicine (2005) **7:**(83-96).

Gottlieb A.B.Psoriasis: Emerging therapeutic strategies.Nature Reviews Drug Discovery (2005) **4:** (19-34).

Sains K.Clinical nutrition - Using nutraceuticals in critical care.Hospital Pharmacist (2005) **12:**(14-16).

Green C.,Colquitt J.L.,Kirby J et al.Topical corticosteroids for atopic eczema: Clinical and cost effectiveness of once-daily vs. more frequent use.British Journal of Dermatology (2005) **152:(**130-141).

Cassano N.,De Meo M.,Scoppio B.MDoes oxidative stress play a role in the pathogenesis of urticarias?.European Journal of Inflammation (2005) **3:** (5-10).

Mease P.,Goffe B.S.Diagnosis and treatment of psoriatic arthritis.Journal of the American Academy of Dermatology (2005) **52:** (1-19).

Brown L.R.Commercial challenges of protein drug delivery.Expert Opinion on Drug Delivery (2005) **2:**(29-42).

2004

Fenton C.,Plosker G.L.Calcipotriol/betamethasone dipropionate: A review of its use in the treatment of psoriasis vulgaris.American Journal of Clinical Dermatology (2004) 5:6 (463-478).

Russo P.A.J.,Ilchef R.,Cooper A.Psychiatric morbidity in psoriasis: A review.Australasian Journal of Dermatology (2004) **45:**(155-160).

Sagoo G.S.,Tazi-Ahnini R.,Barker J.W.N. et al.Meta-analysis of genome-wide studies of psoriasis susceptibility reveals linkage to chromosomes 6p21 and 4q28-q31 in Caucasian and Chinese Hans population.Journal of Investigative Dermatology (2004) **122:** (1401-1405).

Ho V.C. The use of ciclosporin in psoriasis: A clinical review. British Journal of Dermatology, Supplement (2004) 150: (1-10).

Tamibarotene: AM 80, Retinobenzoic acid, Tamibaro.Drugs in R and D (2004) 5:6 (359-362).

Sculpher M.J.,Pang F.S.,Manca A. et al.Generalisability in economic evaluation studies in healthcare: A review and case studies.Health Technology Assessment (2004) **8:**(iii-117).

Ohmori K.,Hasegawa K.,Tamura T.Properties of olopatadine hydrochloride, a new antiallergic/antihistaminic drug.Arzneimittel-Forschung/Drug Research (2004) **54:**(809-829).

Shiina T.,Inoko H.,Kulski J.K.An update of the HLA genomic region, locus information and disease associations: 2004.Tissue Antigens (2004) **64:**(631-649).

Mease P.J.Targeting therapy in psoriatic arthritis.Drug Discovery Today: Therapeutic Strategies (2004) **1:** (389-396).

Stebbins W.G.,Lebwohl M.G.Biologics in combination with nonbiologics: Efficacy and safety.Dermatologic Therapy (2004) 17:5 (432-440).

Bayos M,Rabasseda X.,Prous J.R.Gateways to Clinical Trials.Methods and Findings in Experimental and Clinical Pharmacology (2004) **26:**(723-753).

Cohen M.D.Raising expectations for arthritis treatment: Biologic response modifiers are making remission possible.Postgraduate Medicine(2004)**116:**(41-42+48-50).

Spuls Ph.**I**,Tuut M.K.,Van Everdingen J.J.E. et al The practice guideline 'photo(chemo)therapy and systemic therapy in severe chronic plaque-psoriasis.Nederlands Tijdschrift voor Geneeskunde (2004) **148:** (2121-2125).

Dawson J.K.,Clewes A.R.,Hendry J.Pulmonary effects of low-dose methotrexate therapy.Clinical Pulmonary Medicine (2004) **11:(**307-317).

Ibbotson S.H.,Bilsland D.,Cox N.H et al.An update and guidance on narrowband ultraviolet B phototherapy: A British Photodermatology Group Workshop Report. British Journal of Dermatology (2004) **151:(**283-297).

Mitchell P.B. Australian and New Zealand clinical practice guidelines for the treatment of bipolar disorder. Australian and New Zealand Journal of Psychiatry (2004) **38:(**280-305).

Gambichler T.,Boms S.,Freitag M.Contact dermatitis and other skin conditions in instrumental musicians.BMC Dermatology (2004) 4 Article Number: 3.

Gupta A.K.,Skinner A.R.A review of the use of infliximab to manage cutaneous dermatoses.Journal of Cutaneous Medicine and Surgery (2004) **8** (77-89).

Whysner J.,Reddy M.V.,Ross P.M et al.Genotoxicity of benzene and its metabolitesMutation Research - Reviews in Mutation Research (2004) **566:** (99-130).

Menter A.,Kosinski M.,Bresnahan B.W et al ,Impact of efalizumab on psoriasis-specific patient-reported outcomes. Results from three randomized, placebo-controlled clinical trials of moderate to severe plaque psoriasis.Journal of drugs in dermatology : JDD (2004) **3:**(27-38).

2003

Gupta A.K.,Ryder J.E.Photodynamic Therapy and Topical Aminolevulinic Acid: An Overview. American Journal

of Clinical Dermatology (2003) **4:**(699-708).

Drug and Non-Drug Treatment Strategies to Assist Smoking Cessation.Therapie (2003) **58:** (479-497).

Dawe R.S.A quantitative review of studies comparing the efficacy of narrow-band and broad-band ultraviolet B for psoriasis. British Journal of Dermatology (2003) **149:**(669-672).

Ruderman E.M.Evaluation and management of psoriatic arthritis: The role of biologic therapy.Journal of the American Academy of Dermatology (2003) **49:**2 A (S125-S132).

Goustas P.,Cork M.J.,Higson D.Eumovateâ„¢ (clobetasone butyrate 0.05%) cream: A review of clinical efficacy and safety.Journal of Dermatological Treatment (2003) **14:**(71-85).

Yaqoob P.Lipids and the immune response: From molecular mechanisms to clinical applications.Current Opinion in Clinical Nutrition and Metabolic Care (2003) **6:**(133-150).

Pearson M.M.,Rogers P.D.,Cleary J.D et al.Voriconazole: A new triazole antifungal agent.Annals of Pharmacotherapy (2003) 37:3 (420-432).

2002

Brockbank J.,Gladman D.Diagnosis and management of psoriatic arthritis.Drugs (2002) **62: (**2447-2457).

Goetz C.G.,Koller W.C.,Poewe W et al.MAO-B inhibitors for the treatment of Parkinson's disease.Movement Disorders (2002) 17:SUPPL. 4 (S38-S44).

Krueger G.G.Clinical features of psoriatic arthritis.American Journal of Managed Care (2002) 8:6 SUPPL. (S160-S170).

Sorbera L.A.,Leeson P.A.,Revel L et al.Siplizumab: Antipsoriatic treatment of transplant rejection.Drugs of the Future (2002) **27:(**558-562).

Levin C.,Maibach H.Exploration of alternative and natural drugs in dermatology.
Archives of Dermatology (2002) **138:(**207-211).

2001

Rodewald E.J.,Housman T.S.,Mellen B.G et al.The efficacy of 308nm laser treatment of psoriasis compared to historical controls.Dermatology online journal (2001) **7:(**4).

Darvay A.Phototherapy for psoriasis.CME Bulletin Dermatology (2001) **3:(**9-14).

Vander Zanden J.A.,Valuck R.J.,Bunch C.L et al.Systemic adverse effects of ophthalmic Î²-blockers.Annals of Pharmacotherapy (2001) **35:** (1633-1637).

Kuwano M.,Fukushi J.-I.,Okamoto M et al.A ngiogenesis factors.Internal Medicine (2001) **40:** (565-572).

Faerber L.,Braeutigam M.,Weidinger G et al.Cyclosporine in severe psoriasis: Results of a meta-analysis in 579 patients. American Journal of Clinical Dermatology (2001) **2:**(41-47).

Paleolog E.Anti-TNFÎ± in the treatment of inflammatory diseases. Central-European Journal of Immunology

(2001) **26:(**140-148).

Gambichler T.,Kreuter J.A.,Altmeyer P et al.Meta-analysis of the efficacy of Balneophototherapy.Aktuelle Dermatologie (2000) 26:12 (402-406).

Menter A.Pharmacokinetics and safety of tazarotene. Journal of the American Academy of Dermatology (2000) **43:** III (S31-S35).

Guenther L.Tazarotene combination treatments in psoriasis.Journal of the American Academy of Dermatology (2000) **43:** III (S36-S42).

Lebwohl M.Strategies to optimize efficacy, duration of remission, and safety in the treatment of plaque psoriasis by using tazarotene in combination with a corticosteroid.Journal of the American Academy of Dermatology (2000) **43:** III (S43-S46).

Calcipotriol (DaivonexÂ®) - New administration form.Geneesmiddelenbulletin (2000) **34:(**99-100).

Alarcon G.S.Methotrexate use in rheumatoid arthritis. A clinician's perspective Immunopharmacology (2000) **47:** (259-271).

1999

Asadullah K.,Sabat R.,Wiese A et al.Interleukin-10 in cutaneous disorders: Implications for its pathophysiological importance and therapeutic use.Archives of Dermatological Research (1999) **291:(**628-636).

Rice K.,Spencer J.Inhibitors of human mast cell serine proteases and potential therapeutic applications.Expert Opinion on Therapeutic Patents (1999) **9:(**1537-1555).

Vogler B.K.,Ernst E.Aloe vera: A systematic review of its clinical effectiveness.
British Journal of General Practice (1999) **49:**(823-828).

Shanahan W.R. Jr.ISIS 2302, an antisense inhibitor of intercellular adhesion molecule 1.Expert Opinion on Investigational Drugs (1999) **8:(**1417-1429).

Chuang T.-Y.,Brashear R.,Lewis C. Porphyria cutanea tarda and hepatitis C virus: A case-control study and meta-analysis of the literature. Journal of the American Academy of Dermatology (1999) **41:(**31-36).

Hannuksela-Svahn A.,Pukkala E.,Koulu L et al. Cancer incidence among Finnish psoriasis patients treated with 8- methoxypsoralen bath PUVA. Journal of the American Academy of Dermatology (1999) **40:(**694-696).

Mallon E.,Newson R.,Bunker C.B HLA-Cw6 and the genetic predisposition to psoriasis: A meta-analysis of published serologic studies .Journal of Investigative Dermatology (1999) **113:** (693-695).

Fish oil supplements. Geneesmiddelenbulletin (1999) 33:4 (37-42).

1998

Spuls P.I.,Bossuyt P.M.M.,Van Everdingen J.J.E. et al.The development of practice guidelines for the treatment of severe plaque form psoriasis. Archives of Dermatology (1998) **134:(**1591-1596).

Williams H.C.,Po A.L.W.,Murrel D et al.A systematic review of five systemic treatments for severe psoriasis [15] (multiple letters). British Journal of Dermatology (1998) **139:**(757)

Stern R.S.,Lunder E.J. Risk of squamous cell carcinoma and methoxsalen (psoralen) and UV-A radiation (PUVA): A meta-analysis. Archives of Dermatology (1998) 134:12 (1582-1585).

McNeely W.,Goa K.L.5-methoxypsoralen. A review of its effects in psoriasis and vitiligo. Drugs (1998) 56:4 (667-690).

Koo J.,Arain S. Traditional chinese medicine for the treatment of dermatologic disorders. Archives of Dermatology (1998) **134:(**1388-1393).

Antiseptic/emollient combinations. Drug and Therapeutics Bulletin (1998) **36:(**84-86).

Marchetti A.,LaPensee K.,An P.A pharmacoeconomic analysis of topical therapies for patients with mild- to-moderate stable plaque psoriasis: A US study. Clinical Therapeutics (1998) **20:(**851-869).

Vercauteren S.B.,Bosmans J.-L.,Elseviers M.M et al.A meta-analysis and morphological review of cyclosporine-induced nephrotoxicity in auto-immune diseases. Kidney International (1998) **54:**(536-545).

1997

Peters D.C.,Balfour J.A.Tacalcitol. Drugs (1997) 54:2 (265-272).

Methotrexate in rheumatoid arthritis and psoriasis. Prescrire International (1997) **6:(**96-100).

Mahrle G.Dithranol. Clinics in Dermatology (1997) **15:(**723-737).

Oh P.I.,Gupta A.K.,Einarson T.R et al.Calcipotriol in the treatment of psoriasis of limited severity: Pharmacoeconomic evaluation. Journal of Cutaneous Medicine and Surgery (1997**) 2:(**7-15).

Rosenthal D.,Guenther L.,Kelly J.Current thoughts on the use of methotrexate in patients with psoriasis. Journal of Cutaneous Medicine and Surgery (1997) **2:**(41-46).

Orfanos C.E.,Zouboulis C.C.,Almond-Roesler B et al.Current use and future potential role of retinoids in dermatology. Drugs (1997) **53:(**358-388).

Maha A.,Bobin P.,Coulibaly S et al. Skin diseases revealing human immunodeficiency virus infection in Mali. Annales de Dermatologie et de Venereologie (1997) **124:(**144-150).

1996

Bryson H.M.,Wagstaff A.J. Liarozole. Drugs and Aging (1996) **9:(**478-485).

Lipsky J.J. Mycophenolate mofetil. Lancet (1996) **348:** (1357-1359).

Marks R.Early clinical development of tazarotene. British Journal of Dermatology, Supplement (1996) **135:(**26-31).

1995

Endres S, De Caterina R., Schmidt E.B et al. n-3 Polyunsaturated fatty acids: Update 1995. European Journal of Clinical Investigation (1995) 25:9 (629-638).

Once-a-day topical corticosteroids. Drug and Therapeutics Bulletin(1995)33:(21-22).

Kruger K.Cyclosporin A in the treatment of rheumatoid arthritis and other rheumatic diseases. Zeitschrift fur Rheumatologie (1995) **54:(**89-95).

Chu A.C.,Munn S. Fluticasone propionate in the treatment of inflammatory dermatoses. British Journal of Clinical Practice (1995) **49:(**131-133).

Zachariae H, Steen Olsen T. Efficacy of cyclosporin A (CyA) in psoriasis: An overview of dose/response, indications, contraindications and side-effects. Clinical Nephrology (1995) **43:(**154-158).

1994

Zhang W.Y.,Po A.L.W.The effectiveness of topically applied capsaicin. A meta-analysis. European Journal of Clinical Pharmacology (1994) **46:(**517-522).

Geijer R.M.M.,Meulenberg F.A.E.M.,Ek J.W.T he effectivity of topical drug therapy in psoriasis.Huisarts en Wetenschap (1994) **37:(**243-247+275).

Arikian S.R.,Einarson T.R.,Sander H.M et al.The annual cost of psoriasis. Journal of the American Academy of Dermatology (1994) **30:(**1047-1048).

Einarson T.R.,Arikian S.R.,Shear N.Oral treatments for severe psoriasis: Government payor analysis for Canada. Journal of Dermatological Treatment (1994) 5:SUPPL. 1 (S23-S27).

1991

Whiting-O'Keefe Q.E.,Fye K.H.,Sack K.D.Methotrexate and histologic hepatic abnormalities: A meta-analysis. American Journal of Medicine (1991) **90:**(711-716).

Whiting-O'Keefe Q.E.,Fye K.H.,Sack K.D et al.Methotrexate and histologic hepatic abnormalities: A meta-analysis. Annals of Internal Medicine (1991) 115:6 SUPPL. 2 (61).

1975

Mali J.W.H.,Kuiper J.P.,Van De Staak W.J.B.M et al.Treatment of psoriasis (Dutch). Nederlands Tijdschrift voor Geneeskunde (1975) **119:(**89-93).

Table D. AMSTAR checklist. The tool contains 11 questions (Q1-Q11) with regard to the quality of the review. Every question should be assigned a score 0 or 1. The sum of all scores is the overall quality score of the systematic review.

Item	Question	Responses	Code values
Q1	Was an "a priori" design provided?	Yes[1]/no/Can't answer/NA	1/0/0/0
Q2	Was there duplicate study selection and data extraction?	Yes[2]/no/Can't answer/NA	1/0/0/0
Q3	Was a comprehensive literature search performed?	Yes[3]/no/Can't answer/NA	1/0/0/0
Q4	Was the status of publication (i.e. grey literature) used as an inclusion criterion?	Yes[4]/no/Can't answer/NA	1/0/0/0
Q5	Was a list of studies (included and excluded) provided?	Yes/no/Can't answer/NA	1/0/0/0
Q6	Were the characteristics of the included studies provided?	Yes[5]/no/Can't answer/NA	1/0/0/0
Q7	Was the scientific quality of the included studies assessed and documented?	Yes[6]/no/Can't answer/NA	1/0/0/0
Q8	Was the scientific quality of the included studies used appropriately in formulating conclusions?	Yes[7]/no/Can't answer/NA	1/0/0/0
Q9	Were the methods used to combine the findings of studies appropriate?	Yes[8]/no/Can't answer/NA	1/0/0/0
Q10	Was the likelihood of publication bias assessed?	Yes[9]/no/Can't answer/NA	1/0/0/0
Q11	Was the conflict of interest included?	Yes[10]/no/Can't answer/NA	1/0/0/0

1 The research question and inclusion criteria were established before conducting the review.

2 At least two people working independently extracted the data and the method was reported for reaching consensus if disagreements arose.

3 At least two electronic sources were searched; details of the databases, years searched and search strategy were provided; the search was supplemented by searching of reference lists of included studies, and specialised registers, and by contacting experts.

4 The authors stated that they excluded studies from the review based on publication status. No–authors searched for reports irrespective of publication type. They did not exclude reports based on publication from the systematic review.

5 Data on participants, interventions and outcomes were provided, and the range of relevant characteristics reported.

6 Predetermined methods of assessing quality were reported.

7 The quality (and limitations) of included studies was used in the analysis, conclusions and recommendations of the review

8 If results were pooled statistically, heterogeneity was assessed and used to inform the decision of statistical model to be used. If heterogeneity was present, the appropriateness of combining studies was considered by review authors

9 Publication bias was explicitly considered and assessed.

10 Sources of support were clearly acknowledged.

PROSPERO International prospective register of systematic reviews

Review title and timescale

1 Review title

Give the working title of the review. This must be in English. Ideally it should state succinctly the interventions or exposures being reviewed and the associated health or social problem being addressed in the review.

Quality assessment of pharmacological and non-pharmacological interventions for the management of psoriasis: a systematic review of reviews and network meta-analysis

2 Original language title

For reviews in languages other than English, this field should be used to enter the title in the language of the review. This will be displayed together with the English language title.

3 Anticipated or actual start date

Give the date when the systematic review commenced, or is expected to commence.

20/06/2016

4 Anticipated completion date

Give the date by which the review is expected to be completed.

16/12/2016

5 Stage of review at time of this submission

Indicate the stage of progress of the review by ticking the relevant boxes. Reviews that have progressed beyond the point of completing data extraction at the time of initial registration are not eligible for inclusion in PROSPERO. This field should be updated when any amendments are made to a published record.

The review has not yet started ×

Review stage	Started	Completed
Preliminary searches	Yes	Yes
Piloting of the study selection process	Yes	Yes
Formal screening of search results against eligibility criteria	Yes	Yes
Data extraction	Yes	Yes
Risk of bias (quality) assessment	Yes	Yes
Data analysis	Yes	Yes

Provide any other relevant information about the stage of the review here.

Review team details

6 Named contact

The named contact acts as the guarantor for the accuracy of the information presented in the register record.

Juan Ruano

7 Named contact email

Enter the electronic mail address of the named contact.

juanruanoruiz@mac.com

8 Named contact address

Enter the full postal address for the named contact.

Hospital Universitario Reina Sofia Edificio IMIBIC Avda Menendez Pidal s/n 14004 Cordoba, Spain

9 Named contact phone number

Enter the telephone number for the named contact, including international dialing code.

34606887057

10 Organisational affiliation of the review

Full title of the organisational affiliations for this review, and website address if available. This field may be completed as 'None' if the review is not affiliated to any organisation.

Instituto Maimonides de Investigacion Biomedica de Cordoba (IMIBIC)

Website address:
www.imibic.org

11 Review team members and their organisational affiliations

Give the title, first name and last name of all members of the team working directly on the review. Give the organisational affiliations of each member of the review team.

Title	First name	Last name	Affiliation
Dr	Juan	Ruano	Department of Dermatology, IMIBIC/Reina Sofia University Hospital/University of Cordoba
Mr	Francisco	Gomez-Garcia	Department of Dermatology, IMIBIC/Reina Sofia University Hospital/University of Cordoba
Miss	Beatriz	Maestre-López	School of Medicine, IMIBIC/Reina Sofia University Hospital/University of Cordoba
Dr	Beatriz	Isla-Tejera	Department of Pharmacy, IMIBIC/Reina Sofia University Hospital/University of Cordoba
Dr	Marcelino	González-Padilla	Department of Dermatology, IMIBIC/Reina Sofia University Hospital/University of Cordoba
Mr	Jesus	Gay-Mimbrera	IMIBIC/Reina Sofia University Hospital/University of Cordoba
Miss	Macarena	Aguilar-Luque	IMIBIC/Reina Sofia University Hospital/University of Cordoba
Mr	Pedro	Carmona	IMIBIC/Reina Sofia University Hospital/University of Cordoba
Dr	Antonio	Vélez García-Nieto	Department of Dermatology, IMIBIC/Reina Sofia University Hospital/University of Cordoba
Mr	Juan Luis	Sanz-Cabanillas	Department of Dermatology, IMIBIC/Reina Sofia University Hospital/University of Cordoba

12 Funding sources/sponsors

Give details of the individuals, organizations, groups or other legal entities who take responsibility for initiating, managing, sponsoring and/or financing the review. Any unique identification numbers assigned to the review by the individuals or bodies listed should be included.

Financing: Instituto de Salud Carlos III-Plan de Accion Estrategica en Salud del Programa Estatal de Fomento de la Investigacion Cientifica y Tecnica de Excelencia del Ministerio de Economia y Competitividad (ICI1400136 to JR) and Plan Propio de movilidad para investigadores del Instituto Maimonides de Investigacion Biomedica de Cordoba (IMIBIC) (PP13/009 to JR). There is no funding from any pharmaceutical companies.

13 Conflicts of interest

List any conditions that could lead to actual or perceived undue influence on judgements concerning the main topic investigated in the review.

Are there any actual or potential conflicts of interest?

Yes

JR has received honoraria for lecturing and grants for research from Pfizer, honoraria for lecturing from Janssen-Cilag and Novartis, and other financial benefits from AbbVie and Novartis; AVG-N has received honoraria for lecturing from Pfizer, Novartis, AbbVie, and Janssen- Cilag, and other financial benefits from AbbVie, Novartis, and Janssen-Cilag; FG has received honoraria for research from Pfizer, and for lecturing from AbbVie and Janssen- Cilag; ALL has received honoraria for lecturing from AbbVie and other financial benefit from Novartis; BM-L, BI-T, MG-P, JG-M, MA-L, JLS-C, and PC have no disclosures.

14 Collaborators

Give the name, affiliation and role of any individuals or organisations who are working on the review but who are not listed as review team members.

Title	First name	Last name	Organisation details

Review methods

15 Review question(s)

State the question(s) to be addressed / review objectives. Please complete a separate box for each question.

To investigate the methodological quality of systematic reviews addressing the efficacy and/or safety of pharmacological interventions (biologics, systemics, topical) for chronic plaque psoriasis in adult patients.

To investigate the methodological quality of systematic reviews addressing the efficacy and/or safety of non-pharmacological interventions (phototherapy, skin moistures, diet) for chronic plaque psoriasis in adult patients.

To investigate the quality of reporting of systematic reviews addressing the efficacy and/or safety of pharmacological interventions (biologics, systemics, topical) for chronic plaque psoriasis in pediatric patients.

To investigate the quality of reporting of systematic reviews addressing the efficacy and/or safety of non-pharmacological interventions (phototherapy, skin moistures, diet) for chronic plaque psoriasis in pediatric patients.

To compare the quality of reporting of systematic reviews addressing the efficacy and/or safety of interventional (pharmacological and non-pharmacological) vs observational studies (pharmacogenetics, co-morbidities, etc) for chronic plaque psoriasis in adult or pediatric patients.

To explore if bibliometric factors (journal and researchers) can explain differences in the quality of reporting of systematic reviews in chronic plaque psoriasis.

To explore if the nature of research funding may influence differences in the quality of reporting of systematic reviews in chronic plaque psoriasis.

16 Searches

Give details of the sources to be searched, and any restrictions (e.g. language or publication period). The full search strategy is not required, but may be supplied as a link or attachment.

Search strategies will use a combination of controlled vocabulary, using medical subject headings (MeSH), and text words (e.g., meta-analysis, network meta-analysis, skin psoriasis, human, intervention, biologics, systemic drugs, topics, corticosteroids, clobetasol, calcipotriol, acitretin, metorexate, cyclosporin, phototherapy, infliximab, etanercept, adalimumab, ustekinumab, secukinumab, and ixekinumab). A comprehensive literature search for systematic reviews will be preformed on the electronic databases: MEDLINE (via PubMed and OVID interface, 2000 onwards), EMBASE (OVID interface, 2000 onwards); the Cochrane Library on Wiley [including the Cochrane Database of Systematic Reviews, the Database of Abstract of Reviews of Effects (DARE), and Health Technology Assessment (HTA)]; the Centre for Review and Dissemination (CRD); EBSCOhost; Health Technology Assessment Database; and Web of Science. A grey literature search will be carried using CADTH's Grey Matters tool, and Open-SIGLE (http://www.opengrey.eu/). The PROSPERO database will be searched for ongoing systematic reviews fulfilling our inclusion criteria. Vocabulary and syntax will be adjusted across databases. There will be no language restrictions. Systematic reviews will be included if published between 2000 and 2016 (up until one week before submission).

17 URL to search strategy

If you have one, give the link to your search strategy here. Alternatively you can e-mail this to PROSPERO and we will store and link to it.

I give permission for this file to be made publicly available

Yes

18 Condition or domain being studied

Give a short description of the disease, condition or healthcare domain being studied. This could include health and wellbeing outcomes.

Chronic plaque psoriasis is a chronic inflammatory skin disease mediated by the cells and molecules of both the innate and adaptive immune systems, and affecting 1-3% of the general population. Biologic agents, monoclonal

antibodies or their derivatives, targeting specific immune mediators, have emerged as an alternative treatment option for patients with moderate-to-severe plaque psoriasis who are unresponsive to, or intolerant of, non-biologic systemic agents. During the last decade, these therapies have been widely used in clinical practice setting to treat moderate-severe plaque psoriasis. There are three tumor necrosis factor alpha (TNF-a) antagonists currently available: adalimumab, a fully human monoclonal antibody; etanercept, a soluble receptor construct; and infiximab, a chimeric monoclonal antibody. The remarkable efficacy of these agents in psoriasis placed TNF-a in the center of psoriasis treatment and many other immune-mediated inflammatory diseases for many years. However, our understanding of this disease has progressed greatly and new agents that target the cytokines involved in the IL-23/Th17 pathway have been developed. Several studies have shown that a new generation of monoclonal antibodies which blockade IL-12/23p40 (ustekinumab, briakinumab), IL-23p19 (guselkumab, tildrakizumab), IL-17A (ixekizumab, secukinumab) or IL-17RA (brodalumab) can reverse clinical, histologic, and molecular features of psoriasis in approximately 60-80% of psoriasis patient, as compared with 45-50% in the case of TNF-a antagonists.

19 Participants/population

Give summary criteria for the participants or populations being studied by the review. The preferred format includes details of both inclusion and exclusion criteria.

We will include studies examining the pediatric (less than 18 yrs) or adult patients (18 yrs to 75 yrs) with moderate to severe plaque psoriasis. We will also include studies on people who are overweight or obese, or who have psoriatic arthritis (PsA) but will otherwise exclude studies of populations restricted to specifc diseases, conditions, or metabolic disorders. We will include studies addressing both adults and children if data provided for adults are reported separately.

20 Intervention(s), exposure(s)

Give full and clear descriptions of the nature of the interventions or the exposures to be reviewed

Interventions We are interested in studies addressing efficacy and safety of approved non-pharmacological interventions (e.g, phototherapy, diet-interventions, skin moistures) or licensed drugs for the treatment of moderate-severe plaque psoriasis. Treatments may be placebo, biologics (infiximab, etanercept, adalimumab, ustekinumab, secukinumab, ixekinumab), systemics (acitretin, metotrexato, cyclosporin, apremilast, tofacitinib). Timing Studies will be selected for inclusion based on the length of follow-up of outcomes (induction phase o short-term period; maintenance phase or long-term period).

21 Comparator(s)/control

Where relevant, give details of the alternatives against which the main subject/topic of the review will be compared (e.g. another intervention or a non-exposed control group).

Placebo or standard clinical procedure or other drug or intervention (in head-to-head studies).

22 Types of study to be included

Give details of the study designs to be included in the review. If there are no restrictions on the types of study design eligible for inclusion, this should be stated.

Cochrane or non-Cochrane systematic reviews of primary studies (clinical trials) with and without meta-analysis or network meta-analysis, reporting efficacy and/or safety outcomes. Literature reviews or any primary research report (e.g., clinical trials, observational study) will be excluded.

23 Context

Give summary details of the setting and other relevant characteristics which help define the inclusion or exclusion criteria.

24 Primary outcome(s)

Give the most important outcomes.

Descriptive analysis of methodological quality and quality of reporting of systematic reviews using Assessment of Multiple Systematic Reviews (AMSTAR) instrument and Preferred Reporting Items for Systematic reviews and Meta-Analyses (PRISMA) statement respectively. AMSTAR is a reliable, valid, and feasible measurement tool to assess the methodological quality of systematic reviews. PRISMA is an evidence-based minimum set of items for reporting in systematic reviews and meta-analyses.

Give information on timing and effect measures, as appropriate.

25 Secondary outcomes

List any additional outcomes that will be addressed. If there are no secondary outcomes enter None.

Sub-analysis of AMSTAR scores and PRISMA adherence comparing: a.-Adult vs. pediatric population. b.-Efficacy vs. safety studies. c.-Short-term vs. long-term effects. d.-Pharmacological vs. non-pharmacological interventions. e.-Year of publication. f.-Number of authors. g.-Number of author's disclosures. h.-Country. i.-Language j.-Public vs. non-public institutions. k.-Industry sponsoring. l.-Public funding.

Give information on timing and effect measures, as appropriate.

26 Data extraction (selection and coding)

Give the procedure for selecting studies for the review and extracting data, including the number of researchers involved and how discrepancies will be resolved. List the data to be extracted.

From each eligible study we will extract: the name of the journal, the impact factor (IF) of the journal, the rank of the journal (rank, D1, Q1), the number of authors, the country of the principal investigator (PI), the presence of supporting source (public, private or pharmaceutical industry), the number of author's disclosures, year of publication, characteristics of the study (age of population, type of outcome, type of intervention, language). This task will be performed in duplicate by three researchers separately.

27 Risk of bias (quality) assessment

State whether and how risk of bias will be assessed, how the quality of individual studies will be assessed, and whether and how this will influence the planned synthesis.

This is a systematic review of existing reviews. AMSTAR will be used to assess the methodological quality of systematic reviews. The risk of bias of underlying studies will only be assessed to the extent possible using the information reported in the systematic reviews.

28 Strategy for data synthesis

Give the planned general approach to be used, for example whether the data to be used will be aggregate or at the level of individual participants, and whether a quantitative or narrative (descriptive) synthesis is planned. Where appropriate a brief outline of analytic approach should be given.

Evaluation of methodological quality will be performed using the AMSTAR 11-item instrument. Evaluation of the quality of reporting will be performed by assessing the adherence of each study with the 27-items of the PRISMA statement. These tasks will be performed in duplicate by three researchers separately (JG-M, MA-L, AVG-N) and blinded to the name of the journal, the name of the authors or the affiliations. In case of disagreement an independent fourth researcher (JR) will be consulted. The results will be reported as % of adherence to the PRISMA checklist, overall as well as for each item separately. Comparison of percentages between groups (sub-analysis) will be appraised by the Z test for differences between two proportions or the Fisher test as appropriate, and continuous variables will be evaluated by the non-parametric Wilcoxon rank-sum test. Concordance between each pair of evaluators will be assessed by the Cohen's measure. Statistical analysis and data visualization will be performed using the R programming language.

29 Analysis of subgroups or subsets

Give any planned exploration of subgroups or subsets within the review. ‘None planned’ is a valid response if no subgroup analyses are planned.

Sub-analysis of AMSTAR scores will be performed comparing: a.-Adult vs. pediatric population. b.-Efficacy vs. safety studies. c.-Short-term vs. long-term effects. d.-Pharmacological vs. non-pharmacological interventions. e.-Year of publication. f.-Number of authors. g.-Number of author's disclosures. h.-Country. i.-Language j.-Public vs. non-public institutions. k.-Industry sponsoring. l.-Public funding.

Review general information

30 Type and method of review

Select the type of review and the review method from the drop down list.

Intervention, Network meta-analysis, Review of reviews, Systematic review

31 Language

Select the language(s) in which the review is being written and will be made available, from the drop down list. Use the control key to select more than one language.

English

Will a summary/abstract be made available in English?

Yes

32 Country

Select the country in which the review is being carried out from the drop down list. For multi-national collaborations select all the countries involved. Use the control key to select more than one country.
Spain

33 Other registration details
Give the name of any organisation where the systematic review title or protocol is registered together with any unique identification number assigned. If extracted data will be stored and made available through a repository such as the Systematic Review Data Repository (SRDR), details and a link should be included here.
Extracted data will be stored and made available through the Systematic Review Data Repository (SRDR).

34 Reference and/or URL for published protocol
Give the citation for the published protocol, if there is one.
Give the link to the published protocol, if there is one. This may be to an external site or to a protocol deposited with CRD in pdf format.

I give permission for this file to be made publicly available
Yes

35 Dissemination plans
Give brief details of plans for communicating essential messages from the review to the appropriate audiences.
Do you intend to publish the review on completion?
Yes

36 Keywords
Give words or phrases that best describe the review. (One word per box, create a new box for each term)
Chronic plaque psoriasis

Review of reviews

Methodological quality

Quality of reporting

Biologics

37 Details of any existing review of the same topic by the same authors
Give details of earlier versions of the systematic review if an update of an existing review is being registered, including full bibliographic reference if possible.

38 Current review status
Review status should be updated when the review is completed and when it is published.
Completed but not published

17/02/2017

39 Any additional information
Provide any further information the review team consider relevant to the registration of the review.

40 Details of final report/publication(s)
This field should be left empty until details of the completed review are available.
Give the full citation for the final report or publication of the systematic review.
Give the URL where available.

Table F. Giant components in co-authorship subnetworks.

Subnetwork	Author	H-index	Productivity (total	Co-authors (total	Productivity (SRs about psoriasis)	Institution	City	Country
Low quality<br>Moderate quality	Aractingi, Sélim	39	271	150	25	Hopital Cochin AP-HP, Department of Dermatology	Paris	France
Low quality<br>Moderate quality	Archier, Elodie	8	11	28	8	Aix Marseille Universite, Dermatology and Skin Cancers Department	Marseille	France
Low quality<br>Moderate quality	Aubin, François J.	39	340	>150	22	Centre Hospitalier Universitaire de Besancon, Department of Dermatology	Besancon	France
Low quality<br>Moderate quality	Bachelez, Hervé	36	178	>150	11	Universite Paris 7- Denis Diderot	Paris	France
Low quality	Baddley, John W.	31	129	>150	1	University of Alabama at Birmingham	Birmingham	USA
Low quality<br>Moderate quality	Bagot, Martine	51	474	>150	4	Universite Paris, Laboratoire Immunologie Dermatologie & Oncologie	Paris	France
Moderate quality	Baker, Anna	NA	2	15	2	Atlantis Healthcare	London	UK
Low quality<br>Moderate quality	Barnetche, Thomas	18	54	>150	15	CHU Hopitaux de Bordeaux	Bordeaux	France
Low quality<br>Moderate quality	Beylot-Barry, Marie	32	249	>150	15	Universite de Bordeaux	Bordeaux	France
Low quality<br>Moderate quality	Brénaut, Émilie	7	33	116	15	Centre Hospitalier Universitaire de Brest, Department of Dermatology	Brest	France
Low quality<br>Moderate quality	Bronsard, Valérie	5	57	21	4	Universite Paul Sabatier Toulouse III, Department of Dermatology	Toulouse	France
Moderate quality	Bucknor, Delaney	1	2	11	1	London Metropolitan	London	UK

Subnetwork	Author	H-index	Productivity (total	Co-authors (total	Productivity (SRs about psoriasis)	Institution	City	Country
						University		
Low quality	Calabrese, Leonard H.	60	280	>150	1	Cleveland Clinic Foundation	Cleveland	USA
Low quality<br>Moderate quality	Cantagrel, Alain	41	265	>150	5	Hôpital Pierre-Paul-Riquet, Service of Rheumatology	Toulouse	France
Low quality	Castela, A.	2	2	14	1	Universite Nice Sophia Antipolis, Department of Dermatology	Nice	France
Low quality<br>Moderate quality	Castela, Emeline	8	15	56	7	CHU de Nice, Department of Dermatology	Nice	France
Low quality	Chiller, Tom M.	32	126	>150	1	National Center for Emerging and Zoonotic Infectious Diseases, and Environmental Diseases	Atlanta	USA
Low quality	Collamer, Angelique N.	3	5	8	1	Uniformed Services University of the Health Sciences, San Antonio Military Medical Center	Bethesda	USA
Low quality<br>Moderate quality	Cribier, Bernard J.	42	390	>150	19	CHU Strasbourg, Department of Dermatology	Strasbourg	France
Moderate quality	Demengeot, J.	29	56	>150	1	Instituto Gulbenkian de Ciencia	Oeiras	Portugal
Moderate quality	Depont, Fanny	10	18	65	1	***SC Partners***	Paris	France
Low quality<br>Moderate quality	Devaux, Suzanne	8	11	25	8	Universite Paul Sabatier Toulouse III, Department of Dermatology	Toulouse	France
Moderate quality	Filippi, Jérôme Ôme	17	47	>150	1	Hopital l'Archet, Hepato-gastroenterology Unit	Nice	France
Moderate quality	Fisher, Abigail	NA	1	9	1	***UCL***	London	UK
Low quality<br>Moderate quality	Gallini, Adeline	14	35	86	11	CHU de Toulouse, Faculty of Medicine	Toulouse	France
Low quality	Galván-Banqueri, Mercedes	5	17	29	1	Andalusian Agency for Health Technology Assessment	Sevilla	Spain

Subnetwork	Author	H-index	Productivity (total	Co-authors (total	Productivity (SRs about psoriasis)	Institution	City	Country
Moderate quality	Garcês, Sandra	7	11	>150	1	Hospital Garcia de Orta, Department of Rheumatology	Almada	Portugal
Low quality<br>Moderate quality	Gourraud, Pierre Antoine F D	27	90	>150	4	UCSF Multiple Sclerosis Center	San Francisco	USA
Low quality<br>Moderate quality	Horreau, C.	4	5	19	4	Hopital Nord AP-HM, Service of Dermatology	Marseille	France
Low quality<br>Moderate quality	Joly, Pascal	48	402	>150	24	CHU Hopitaux de Rouen, Department of Dermatology	Rouen	France
Low quality<br>Moderate quality	Jullien, Denis	36	144	>150	24	Hopital Edouard Herriot, Department of Dermatology	Lyon	France
Moderate quality	Lacerda, Ana P.	NA	1	9	1	***AbbVie, North Chicago***	Chicago	USA
Low quality<br>Moderate quality	Le Maître, Michel	18	106	>150	25	Dermatologist	Caen	France
Moderate quality	Levstik, Mark A.	15	26	>150	1	London Health Sciences Centre	London	UK
Low quality	Lipman, Marc CI I	36	198	>150	1	National Health Service	London	UK
Low quality	Lortholary, O.	1	2	17	1	Universite Paris Descartes	Paris	France
Low quality	Mariette, Xavier	67	426	>150	1	Centre de Recherche en Immunologie des Infections virales et des maladies autoimmunes	Le Kremlin-Bicetre	France
Low quality	Marín Gil, Roberto	3	11	37	1	Hospital Universitario Virgen del Rocio, Unidad de Gestión Clínica de Farmacia	Sevilla	Spain
Moderate quality	Maza, Aude	8	26	109	3	CHU de Toulouse, Department of Dermatology	Nice	France
Low quality<br>Moderate quality	Misery, Laurent	36	394	150	21	Centre Hospitalier Universitaire de Brest, Department of Dermatology	Brest	France
Moderate quality	Montaudié, Henri	6	25	81	3	CHU de Nice, Department of Dermatology	Nice	France

Subnetwork	Author	H-index	Productivity (total	Co-authors (total	Productivity (SRs about psoriasis)	Institution	City	Country
Moderate quality	Mrowietz, Ulrich	50	258	>150	1	Universitatsklinikum Schleswig-Holstein Campus Kiel, Psoriasis-Center at the Department of Dermatology	Kiel	Germany
Moderate quality	Nataf, Henri	5	14	67	1	Cabinet de Rheumatologie	Mantes-la-Jolie	France
Low quality	Novosad, Shannon A.	3	10	67	1	National Center for Emerging and Zoonotic Infectious Diseases, Division of Healthcare Quality Promotion	Atlanta	USA
Low quality<br>Moderate quality	Ortonne, Jean Paul	36	180	>150	24	Hopital l'Archet, Department of Dermatology	Nice	France
Moderate quality	Östör, Andrew Jk K	22	84	>150	1	University of Cambridge, Rheumatology Research Unit	Cambridge	UK
Low quality<br>Moderate quality	Paul, Carle F.	39	238	>150	26	Universite Paul Sabatier Toulouse III, Department of Maxillofacial Surgery	Toulouse	France
Moderate quality	Peyrin-Biroulet, Laurent	NA	2	16	2	Universite de Lorrain, Department of Gastroenterology	Nancy	France
Low quality	Polgreen, Philip M.	23	140	>150	1	University of Iowa Caver College of Medicine, College of Public Health	Iowa City	USA
Low quality<br>Moderate quality	Pouplard, C	5	6	36	4	Universite Paul Sabatier Toulouse III, Department of Dermatology	Toulouse	France
Low quality<br>Moderate quality	Prey, Sorilla	35	14	>150	5	Hopital des Enfants, Service de Dermatologie Pediatrique et Centre de Reference des Maladies Rares de la Peau	Toulouse	France
Low quality<br>Moderate quality	Puzenat, Ève	15	57	>150	4	Centre Hospitalier Universitaire de Besancon, Department of Dermatology	Besancon	France
Low quality	Richard-Lallemand, M	8	16	50	4	Universite Paul Sabatier	Toulouse	France

Subnetwork	Author	H-index	Productivity (total	Co-authors (total	Productivity (SRs about psoriasis)	Institution	City	Country
Moderate quality	arie Aleth					Toulouse III, Department of Dermatology		
Low quality<br>Moderate quality	Richard, Marie Aleth	28	163	>150	20	Hopital La Timone	Marseille	France
Low quality<br>Moderate quality	Rouzaud, Marie	5	6	14	5	Universite de Bordeaux, Department of Dermatology	Bordeaux	France
Low quality	Saag, Michael S.	2	4	>150	1	University of Alabama at Birmingham, Division of Infectious Diseases	Birmingham	USA
Low quality	Santos Ramos, Bernardo	8	56	114	1	Unidad de Gestión Clínica de Genética	Sevilla	Spain
Moderate quality	Sbidian, Émilie	10	57	>150	3	Universite Paris-Est	Marne-la-Vallee	France
Low quality<br>Moderate quality	Sevrain, Morgane	5	7	16	5	Centre Hospitalier Universitaire de Brest, Department of Dermatology	Brest	France
Low quality	Smolen, Josef Sebastian	103	819	>150	1	Medizinische Universitat Wien, Department of Medicine	Vienna	Austria
Moderate quality	Thibout, Emmanuel	2	6	16	1	***AbbVie, North Chicago***	Chicago	USA
Moderate quality	Vangeli, Eleni	9	13	40	1	London South Bank University, Department of Psychology	London	UK
Low quality<br>Moderate quality	Villani, Axel Patrice Atrice	6	27	54	5	Hopital Edouard Herriot, Department of Dermatology	Lyon	France
Low quality	Weinblatt, Michael E.	67	291	>150	1	Brigham and Women's Hospital, Division of Rheumatology, Immunology and Allergy	Boston	USA
Moderate quality	Weinman, John A.	57	234	>150	1	King's College London, Institute of Pharmaceutical Science		

Subnetwork	Author	H-index	Productivity (total	Co-authors (total	Productivity (SRs about psoriasis)	Institution	City	Country
Low quality	Winthrop, Kevin L.	41	147	>150	1	University of Alabama at Birmingham, Department of Medicine	Birmingham	USA
High quality	Bessette, Louis	23	73	>150	1	Universite Laval, Department of Medicine	Quebec	Canada
High quality	Blankenship, Derek	1	2	13	2	Baylor University Medical Center at Dallas, Department of Research and Improvement Education	Dallas	USA
High quality	Daoud, Yahya A	6	9	49	1	Baylor Research Institute, Department of Dermatology	Dallas	USA
High quality	De Lemos, James A.	68	391	>150	1	UT Southwestern Medical School, Department of Internal Medicine	Dallas	USA
High quality	Dutz, Jan Peter	31	114	>150	1	The University of British Columbia, Department of Dermatology and Skin Science	Vancouver	Canada
High quality	Fleming, Patrick James	6	20	37	1	University of Toronto, Division of Dermatology	Toronto	Canada
High quality	Friedewald, Vincent E	11	63	139	1	American Journal of Cardiology	-	USA
High quality	Gordon, Kenneth B	48	127	>150	1	Northwestern University Feinberg School of Medicine, Department of Dermatology	Chicago	USA
High quality	Gulliver, Wayne P F	25	86	>150	1	Memorial University of Newfoundland, Faculty of Medicine, Department of Medicine	St John's	Canada
High quality	Haraoui, Boulos Paul	36	150	>150	1	Universite de Montreal	Montreal	Canada
High quality	Kaplan, Daniel H.	26	60	>150	1	University of Pittsburgh, Department of Immunology	Pittsburgh	USA
High quality	Kazi, Salahuddin	11	17	67	1	UT Southwestern Medical Center, Rheumatic Diseases	Dallas	USA

Subnetwork	Author	H-index	Productivity (total	Co-authors (total	Productivity (SRs about psoriasis)	Institution	City	Country
						Division		
High quality	Keeling, Stephanie O.	8	19	111	1	Memorial University of Newfoundland, Faculty of Medicine	St John's	Canada
High quality	Kimball, Alexa Boer Oer	39	223	>150	1	Massachusetts General Hospital, Department of Dermatology	Boston	USA
High quality	Kraft, John N.	8	19	30	2	Lynde Centre for Dermatology	Markham	Canada
High quality	Krueger, James G.	79	270	>150	1	Rockefeller University, Laboratory for Investigative Dermatology	New York	USA
High quality	Leonardi, Craig L.	41	126	>150	1	St. Louis University School of Medicine, Department of Dermatology	St Louis	USA
High quality	Lynde, Charles W.	31	144	>150		University of Toronto, Division of Dermatology	Toronto	Canada
High quality	McCourt, Collette	3	7	19	1	The University of British Columbia, Department of Dermatology and Skin Science	Vancouver	Canada
High quality	McFarlane, Alexandra	3	8	20	1	University of Alberta, Division of Rheumatology	Edmonton	Canada
High quality	Menter, Alan M	60	278	>150	1	Baylor University Medical Center at Dallas, Department of Dermatology	Dallas	USA
High quality	Pope, Janet Elizabeth	2	4	21	1	Centre for Rheumatology, St Joseph's Health Centre	London	UK
High quality	Richer, Vincent	4	14	32	1	St-Luc Hospital, Department of Medicine	Quebec	Canada
High quality	Roubille, Camille	9	35	106	1	Universite de Montreal	Montreal	Canada
High quality	Ryan, Caitriona	14	32	97	1	Texas A and M Health Science Center, Department of Medical Education, College Station	Texas	USA
High quality	Siu, Stephanie	3	6	17	1	University of the West,	London	UK

Subnetwork	Author	H-index	Productivity (total	Co-authors (total	Productivity (SRs about psoriasis)	Institution	City	Country
						Division of Rheumatology		
High quality	Starnino, Tara	3	7	19	1	Universite de Montreal, Sacré-Coeur Hospital	Montreal	Canada
High quality	Strober, Bruce E.	31	119	>150	1	University of Connecticut School of Medicine, Department of Dermatology	Farmington	USA

2. Supplementary Figures.

Figure A. PRISMA Flowchart for Overview of Systematic Reviews and Meta- analysis on psoriasis included and excluded in the study.

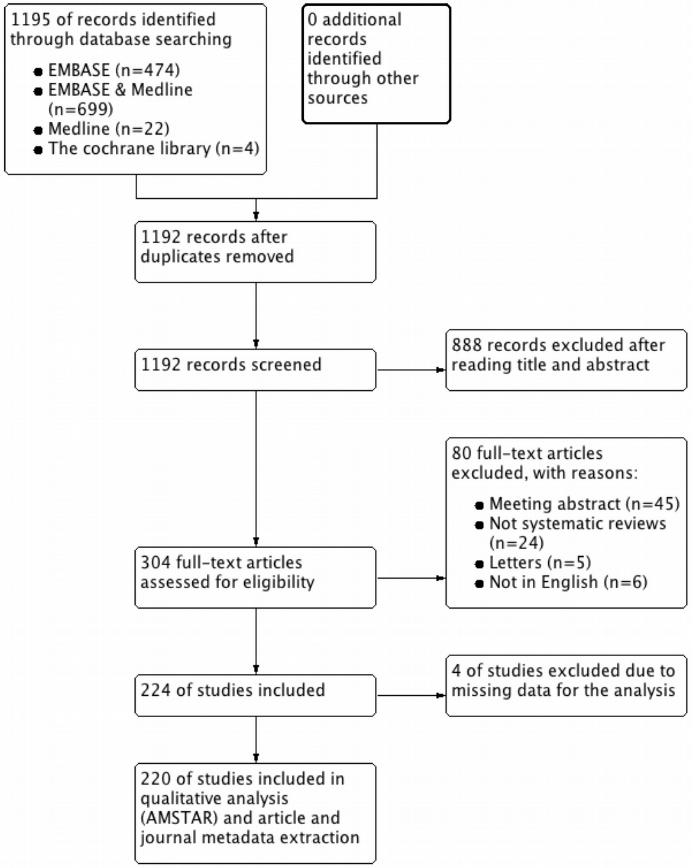

Figure B. Co-authorship networks modularity.

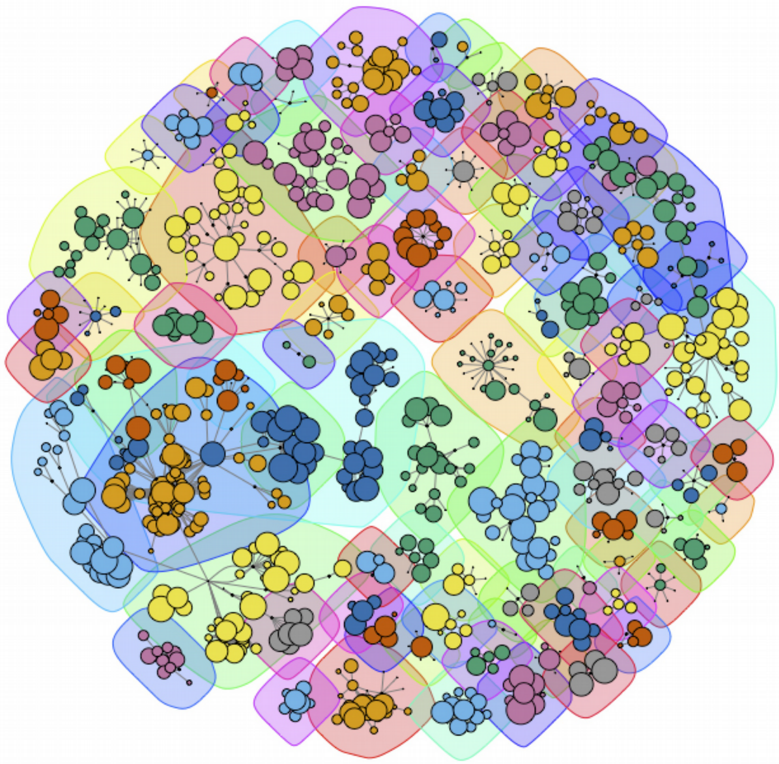

Figure C. Community analysis of co-authorship of low, moderate, and high methodological quality reviews derived subnetworks.

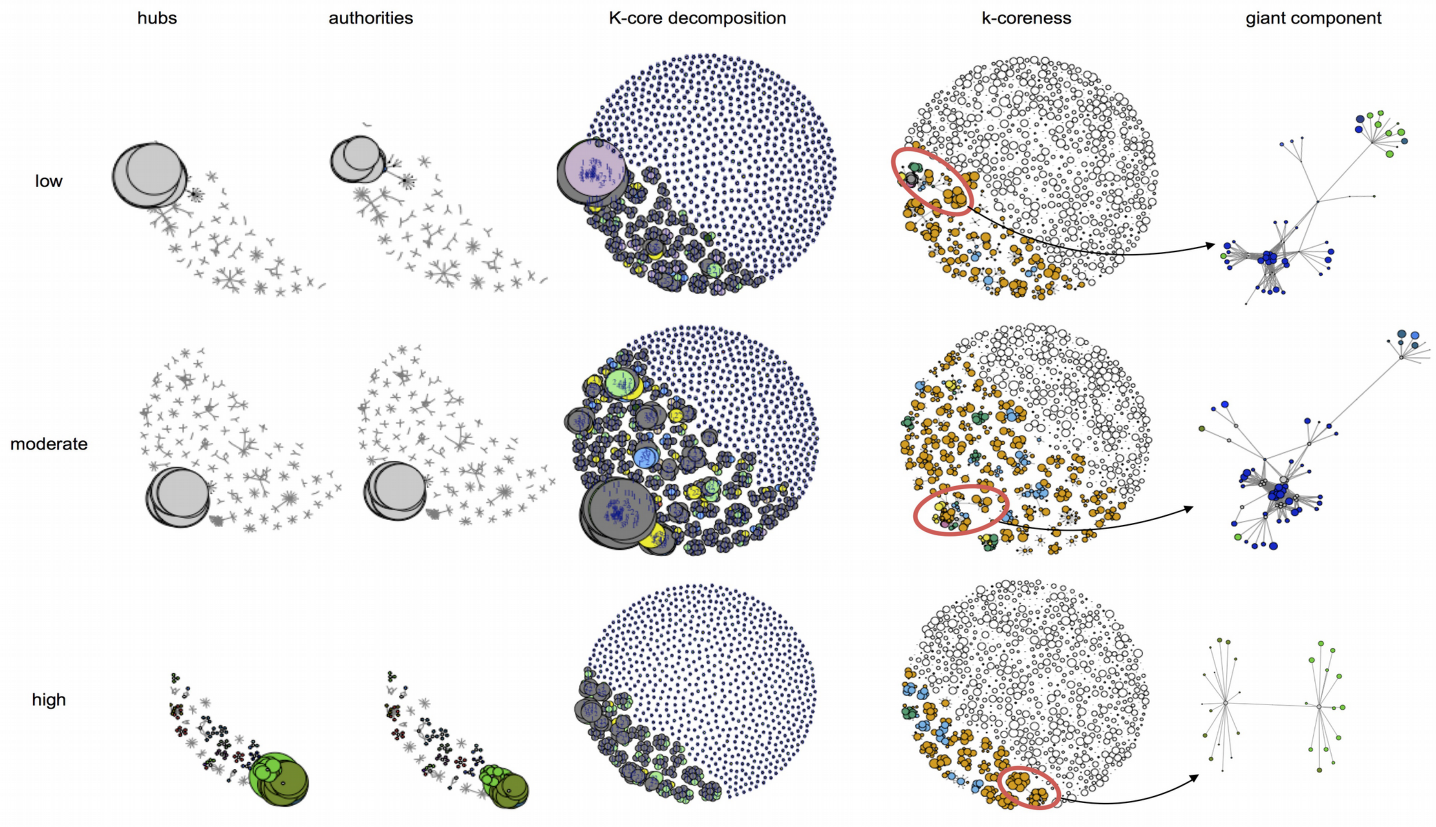

Figure D. Visualization of unique and intersection analysis of authors who have published systematic reviews classified by methodological quality and author productivity. Productivity is the number of published papers. Low vs high productivity subsets have been defined using the median of productivity as a threshold.

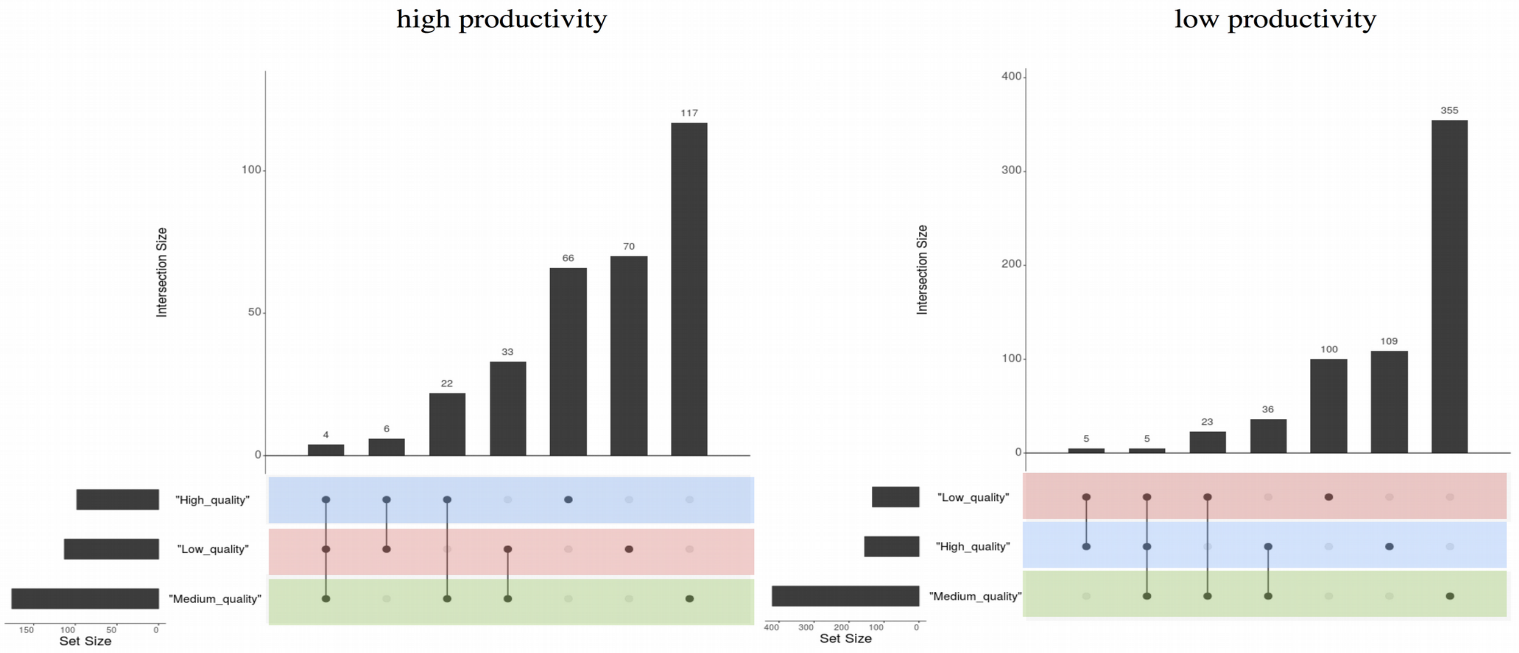

Supplement: S1 File — Search strategy for identifying systematic reviews and meta-analyses in EMBASE (Table A), list of included studies (Table B), list of non included studies (Table C), AMSTAR checklist (Table D), PROSPERO register file (Table E), giant components in co-authorship subnetworks (Table F), PRISMA Flowchart for Overview of Systematic Reviews and Meta-analysis on psoriasis included and excluded in the study (Fig A), co-authorship networks modularity (Fig B), community analysis of co-authorship of low, moderate, and high methodological quality reviews derived subnetworks (Fig C), visualization of unique and intersection analysis of authors who have published systematic reviews classified by methodological quality and author productivity (Fig D). (PDF) [file pone.0175419.s001.pdf]
